# Impaired IL-23–dependent induction of IFN-γ underlies mycobacterial disease in patients with inherited TYK2 deficiency

**DOI:** 10.1084/jem.20220094

**Published:** 2022-09-12

**Authors:** Masato Ogishi, Andrés Augusto Arias, Rui Yang, Ji Eun Han, Peng Zhang, Darawan Rinchai, Joshua Halpern, Jeanette Mulwa, Narelle Keating, Maya Chrabieh, Candice Lainé, Yoann Seeleuthner, Noé Ramírez-Alejo, Nioosha Nekooie-Marnany, Andrea Guennoun, Ingrid Muller-Fleckenstein, Bernhard Fleckenstein, Sara S. Kilic, Yoshiyuki Minegishi, Stephan Ehl, Petra Kaiser-Labusch, Yasemin Kendir-Demirkol, Flore Rozenberg, Abderrahmane Errami, Shen-Ying Zhang, Qian Zhang, Jonathan Bohlen, Quentin Philippot, Anne Puel, Emmanuelle Jouanguy, Zahra Pourmoghaddas, Shahrzad Bakhtiar, Andre M. Willasch, Gerd Horneff, Genevieve Llanora, Lynette P. Shek, Louis Y.A. Chai, Sen Hee Tay, Hamid H. Rahimi, Seyed Alireza Mahdaviani, Serdar Nepesov, Aziz A. Bousfiha, Emine Hafize Erdeniz, Adem Karbuz, Nico Marr, Carmen Navarrete, Mehdi Adeli, Lennart Hammarstrom, Hassan Abolhassani, Nima Parvaneh, Saleh Al Muhsen, Mohammed F. Alosaimi, Fahad Alsohime, Maryam Nourizadeh, Mostafa Moin, Rand Arnaout, Saad Alshareef, Jamila El-Baghdadi, Ferah Genel, Roya Sherkat, Ayça Kiykim, Esra Yücel, Sevgi Keles, Jacinta Bustamante, Laurent Abel, Jean-Laurent Casanova, Stéphanie Boisson-Dupuis

**Affiliations:** 1 St Giles Laboratory of Human Genetics of Infectious Diseases, Rockefeller Branch, The Rockefeller University, New York, NY; 2 Primary Immunodeficiencies Group, University of Antioquia, Medellin, Colombia; 3 School of Microbiology, University of Antioquia, Medellin, Colombia; 4 Walter and Eliza Hall Institute of Medical Research, Department of Medical Biology, University of Melbourne, Melbourne, Australia; 5 Laboratory of Human Genetics of Infectious Diseases, Necker Branch, INSERM U1163, Paris, France; 6 Paris Cité University, Imagine Institute, Paris, France; 7 Acquired Immunodeficiency Research Center, Isfahan University of Medical Sciences, Isfahan, Iran; 8 Sidra Medicine, Doha, Qatar; 9 Institute of Clinical and Molecular Virology, University of Erlangen-Nuremberg, Erlangen, Germany; 10 Department of Pediatric Immunology and Rheumatology, Faculty of Medicine, Uludag University, Bursa, Turkey; 11 Division of Molecular Medicine, Institute of Advanced Medical Sciences, Tokushima University, Tokushima, Japan; 12 Institute for Immunodeficiency, Center for Chronic Immunodeficiency, Medical Center, Faculty of Medicine, University of Freiburg, Freiburg, Germany; 13 Eltern- Kind -Zentrum Prof. Hess, Klinikum Bremen–Mitte, Bremen, Germany; 14 Department of Pediatric Genetics, Umraniye Training and Research Hospital, University of Health Sciences, Istanbul, Turkey; 15 Laboratory of Virology, Assistance Publique-Hôpitaux de Paris, Cochin Hospital, Paris, France; 16 Laboratory of Clinical Immunology, Inflammation and Allergy, Faculty of Medicine and Pharmacy, Hassan II University, Casablanca, Morocco; 17 Department of Pediatric Infectious Disease, Isfahan University of Medical Sciences, Isfahan, Iran; 18 Division for Stem Cell Transplantation, Immunology and Intensive Care Medicine, Department for Child and Adolescent Medicine, University Hospital Frankfurt, Frankfurt, Germany; 19 Center for Pediatric Rheumatology, Department of Pediatrics, Asklepios Clinic Sankt Augustin, Sankt Augustin, Germany; 20 Medical Faculty, University of Cologne, Cologne, Germany; 21 Division of Allergy and Immunology, Department of Paediatrics, Khoo Teck Puat - National University Children’s Medical Institute, National University Health System, Singapore; 22 Department of Pediatrics, National University of Singapore, Singapore; 23 Division of Infectious Diseases, Department of Medicine, National University Health System, Singapore; 24 Synthetic Biology for Clinical and Technological Innovation, Life Sciences Institute; Synthetic Biology Translational Research Program, National University of Singapore; 25 Department of Medicine, Yong Loo Lin School of Medicine, National University of Singapore, Singapore; 26 Division of Rheumatology, Department of Medicine, National University Hospital, Singapore; 27 Department of Pediatrics, Isfahan University of Medical Sciences, Isfahan, Iran; 28 Pediatric Respiratory Diseases Research Center, National Research Institute of Tuberculosis and Lung Diseases, Shahid Beheshti University of Medical Sciences, Tehran, Iran; 29 Department of Pediatric Allergy and Immunology, Istanbul Medipol University, Istanbul, Turkey; 30 Clinical Immunology Unit, Department of Pediatrics, King Hassan II University, Ibn-Rochd Hospital, Casablanca, Morocco; 31 Division of Pediatric Infectious Diseases, Ondokuz Mayıs University, Samsun, Turkey; 32 Division of Pediatric Infectious Diseases, Okmeydani Training and Research Hospital, University of Health Sciences, Istanbul, Turkey; 33 Department of Immunology, Hospital de Niños Roberto del Río, Santiago de Chile, Chile; 34 Division of Allergy and Immunology, Sidra Medicine/Hamad Medical Corp., Doha, Qatar; 35 Department of Biosciences and Nutrition, Karolinska Institute, Stockholm, Sweden; 36 Beijing Genomics Institute, Shenzhen, China; 37 Research Center for Immunodeficiencies, Pediatrics Center of Excellence, Children’s Medical Center, Tehran University of Medical Sciences, Tehran, Iran; 38 Immunology Research Laboratory, Department of Pediatrics, College of Medicine, King Saud University, Riyadh, Saudi Arabia; 39 Pediatric Department, College of Medicine, King Saud University, Riyadh, Saudi Arabia; 40 Pediatric Intensive Care Unit, King Saud University Medical City, Riyadh, Saudi Arabia; 41 Immunology, Asthma and Allergy Research Institute, Tehran University of Medical Sciences, Tehran, Iran; 42 Children's Medical Center, Pediatrics Center of Excellence, Tehran University of Medical Sciences, Tehran, Iran; 43 Section of Allergy & Immunology, Department of Medicine, King Faisal Specialist Hospital and Research Center, Riyadh, Saudi Arabia; 44 Al Faisal University, Riyadh, Saudi Arabia; 45 Genetics Unit, Mohamed V Military Hospital, Hay Riad, Rabat, Morocco; 46 University of Health Sciences, Dr Behçet Uz Children’s Hospital, Division of Pediatric Immunology, Izmir, Turkey; 47 Pediatric Allergy and Immunology, Cerrahpasa Medical Faculty, Istanbul University-Cerrahpasa, Istanbul, Turkey; 48 Division of Pediatric Allergy and Immunology, Istanbul Faculty of Medicine, Istanbul University, Istanbul, Turkey; 49 Division of Pediatric Allergy and Immunology, Meram Medical Faculty, Necmettin Erbakan University, Konya, Turkey; 50 Center for the Study of Primary Immunodeficiencies, Assistance Publique-Hôpitaux de Paris, Necker Hospital for Sick Children, Paris, France; 51 Howard Hughes Medical Institute, New York, NY; 52 Deparment of Pediatrics, Necker Hospital for Sick Children, Paris, France

## Abstract

Human cells homozygous for rare loss-of-expression (LOE) *TYK2* alleles have impaired, but not abolished, cellular responses to IFN-α/β (underlying viral diseases in the patients) and to IL-12 and IL-23 (underlying mycobacterial diseases). Cells homozygous for the common P1104A *TYK2* allele have selectively impaired responses to IL-23 (underlying isolated mycobacterial disease). We report three new forms of TYK2 deficiency in six patients from five families homozygous for rare *TYK2* alleles (R864C, G996R, G634E, or G1010D) or compound heterozygous for P1104A and a rare allele (A928V). All these missense alleles encode detectable proteins. The R864C and G1010D alleles are hypomorphic and loss-of-function (LOF), respectively, across signaling pathways. By contrast, hypomorphic G996R, G634E, and A928V mutations selectively impair responses to IL-23, like P1104A. Impairment of the IL-23–dependent induction of IFN-γ is the only mechanism of mycobacterial disease common to patients with complete TYK2 deficiency with or without TYK2 expression, partial TYK2 deficiency across signaling pathways, or rare or common partial TYK2 deficiency specific for IL-23 signaling.

## Teaser

This study characterizes three novel forms of autosomal recessive human TYK2 deficiency. Impairment of IL-23–dependent induction of IFN-γ is the only mechanism of mycobacterial disease common to patients with any of the five known forms of autosomal recessive TYK2 deficiency.

## Introduction

Mendelian susceptibility to mycobacterial disease (MSMD) is characterized by severe diseases caused by weakly virulent mycobacteria, such as bacillus Calmette-Guérin (BCG) vaccines and environmental mycobacteria (EM) in otherwise healthy patients, normally resistant to other microorganisms and without overt immunodeficiency (Casanova and Abel, 2002, 2018, 2022; Bustamante et al., 2014; Bustamante, 2020; Boisson-Dupuis and Bustamante, 2021). Patients with “isolated MSMD” have the canonical MSMD phenotype, whereas patients with “syndromic MSMD” also typically display other clinical phenotypes, infectious or otherwise. Germline mutations of 19 genes underlie 34 forms of MSMD due to allelic heterogeneity (Bustamante, 2020; Kerner et al., 2020; Le Voyer et al., 2021; Martin-Fernandez et al., 2022; Yang et al., 2020). Most known genetic etiologies of MSMD affect the production of or cellular response to IFN-γ, highlighting the indispensable role of this cytokine in the control of mycobacteria (Boisson-Dupuis and Bustamante, 2021; Boisson-Dupuis et al., 2018; Bustamante, 2020; Kerner et al., 2020; Le Voyer et al., 2021; Martinez-Barricarte et al., 2018; Yang et al., 2020). One possible exception is ZNFX1 deficiency, for which the pathogenic mechanism remains unknown (Le Voyer et al., 2021). Human IFN-γ has been shown to function more as a macrophage-activating factor than as an antiviral interferon (Nathan et al., 1983). The susceptibility of IFN-γ–deficient mice to weakly virulent mycobacteria is consistent with these findings (Dalton et al., 1993; Doherty and Sher, 1997; Kamijo et al., 1993). Inborn errors of IFN-γ immunity can also underlie infections caused by *Mycobacterium tuberculosis* (*M.tb*), which is ≥1,000 times more virulent than BCG (Boisson-Dupuis, 2020; Boisson-Dupuis and Bustamante, 2021; Ogishi et al., 2021; Casanova and Abel, 2022), and a few other intramacrophagic pathogens, including bacteria (e.g., *Salmonella*), parasites (e.g., *Leishmania*), and fungi (e.g., *Histoplasma*; Arias et al., 2017; Bustamante, 2020; Bustamante et al., 2014; de Beaucoudrey et al., 2010; Parvaneh et al., 2017; Tan et al., 2016; van de Vosse et al., 2013). Patients with syndromic MSMD display associated phenotypes: patients with ISG15 deficiency have features of type I interferonopathy (Bogunovic et al., 2012; Martin-Fernandez et al., 2020; Zhang et al., 2015), patients with RORγ/RORγT deficiency have chronic mucocutaneous candidiasis (Okada et al., 2015), patients with ZNFX1 deficiency have monocytosis (Le Voyer et al., 2021), the only patient with T-bet deficiency reported to date has airway hyperresponsiveness (Yang et al., 2021), and patients with JAK1 or TYK2 deficiencies have viral diseases (Eletto et al., 2016; Kreins et al., 2015).

Autosomal recessive (AR) complete TYK2 deficiency is characterized by mycobacterial and/or viral diseases (Table 1; Fuchs et al., 2016; Guo et al., 2020; Kreins et al., 2015; Minegishi et al., 2006; Sarrafzadeh et al., 2020; Wu et al., 2020; Zhang et al., 2022). Only 15 patients with AR TYK2 deficiency from 13 families have been reported (including one for whom functional characterization is incomplete; Table 1; Fuchs et al., 2016; Guo et al., 2020; Kilic et al., 2012; Kreins et al., 2015; Minegishi et al., 2006; Sarrafzadeh et al., 2020; Wu et al., 2020; Zhang et al., 2022). Nine of these patients had mycobacterial diseases, including BCG disease (*n* = 6), EM disease (*n* = 1), and tuberculosis (TB; *n* = 3), and five had unusually severe viral illnesses, including mucocutaneous herpes simplex virus 1 (HSV-1) infections (*n* = 3), HSV-1 encephalitis (HSE; *n* = 1), cutaneous varicella-zoster virus (VZV; *n* = 2) or *Molluscum contagiosum* (*n* = 1) infections, human parainfluenza type 3 virus (PIV3) pneumonia (*n* = 1), COVID-19 pneumonia (*n* = 4), influenza A pneumonia (*n* = 1), and measles-mumps-rubella (MMR) vaccine disease (*n* = 1; Fuchs et al., 2016; Guo et al., 2020; Kilic et al., 2012; Kreins et al., 2015; Minegishi et al., 2006; Sarrafzadeh et al., 2020; Wu et al., 2020; Zhang et al., 2022). AR TYK2 deficiency impairs, but does not abolish, cellular responses to IL-10, IL-12, IL-23, and IFN-α/β (Kreins et al., 2015; Minegishi et al., 2006). Poor responses to IFN-α/β in most if not all cell types underlie viral diseases, whereas poor IFN-γ induction in lymphocytes stimulated with IL-12 or IL-23 underlies mycobacterial diseases. Patient P-Jap (Minegishi et al., 2006) was the only TYK2-deficient patient reported to suffer from chronic mucocutaneous candidiasis, which was attributed to impaired IL-12 and IL-23 responses and defective Th17 immunity, as seen in patients with IL-12Rβ1 deficiency (de Beaucoudrey et al., 2008). The poor response to IL-10 of the patients’ leukocytes does not appear to be associated with the early-onset colitis seen in patients with AR IL-10, IL-10RA, or IL-10RB deficiencies (Engelhardt and Grimbacher, 2014; Engelhardt et al., 2013; Glocker et al., 2009; Glocker et al., 2011), possibly because of residual TYK2-independent responses to IL-10. Intriguingly, one patient (P-Ger) also had high serum IgE levels (Fuchs et al., 2016), whereas another (P-Jap) also had eczema and staphylococcal skin infections, as seen in patients with hyper-IgE syndrome (HIES; Fuchs et al., 2016; Minegishi et al., 2006). The HIES phenotype of P-Jap may be due to impaired fibroblastic responses to IL-6 (Minegishi et al., 2006), a typical feature of the four genetic etiologies of HIES: STAT3, ZNFX341, IL6ST, and IL6R deficiencies (Minegishi et al., 2007; Béziat et al., 2018, 2020; Puel and Casanova, 2019). However, the IL-6 defect in this patient was not rescued by WT TYK2 (Kreins et al., 2015). Moreover, P-Ger had an apparently normal cellular response to IL-6 (Fuchs et al., 2016). HIES and high serum IgE levels in these two patients may, therefore, have been driven by genetic variants at loci other than *TYK2*, either alone or together with TYK2 deficiency.

**Table 1.** Genotype, phenotype, and molecular characterization of patients with TYK2 deficiency

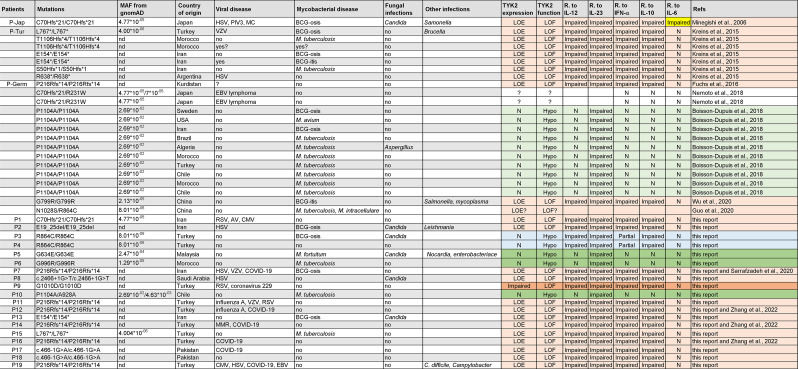

**Figure 1.**
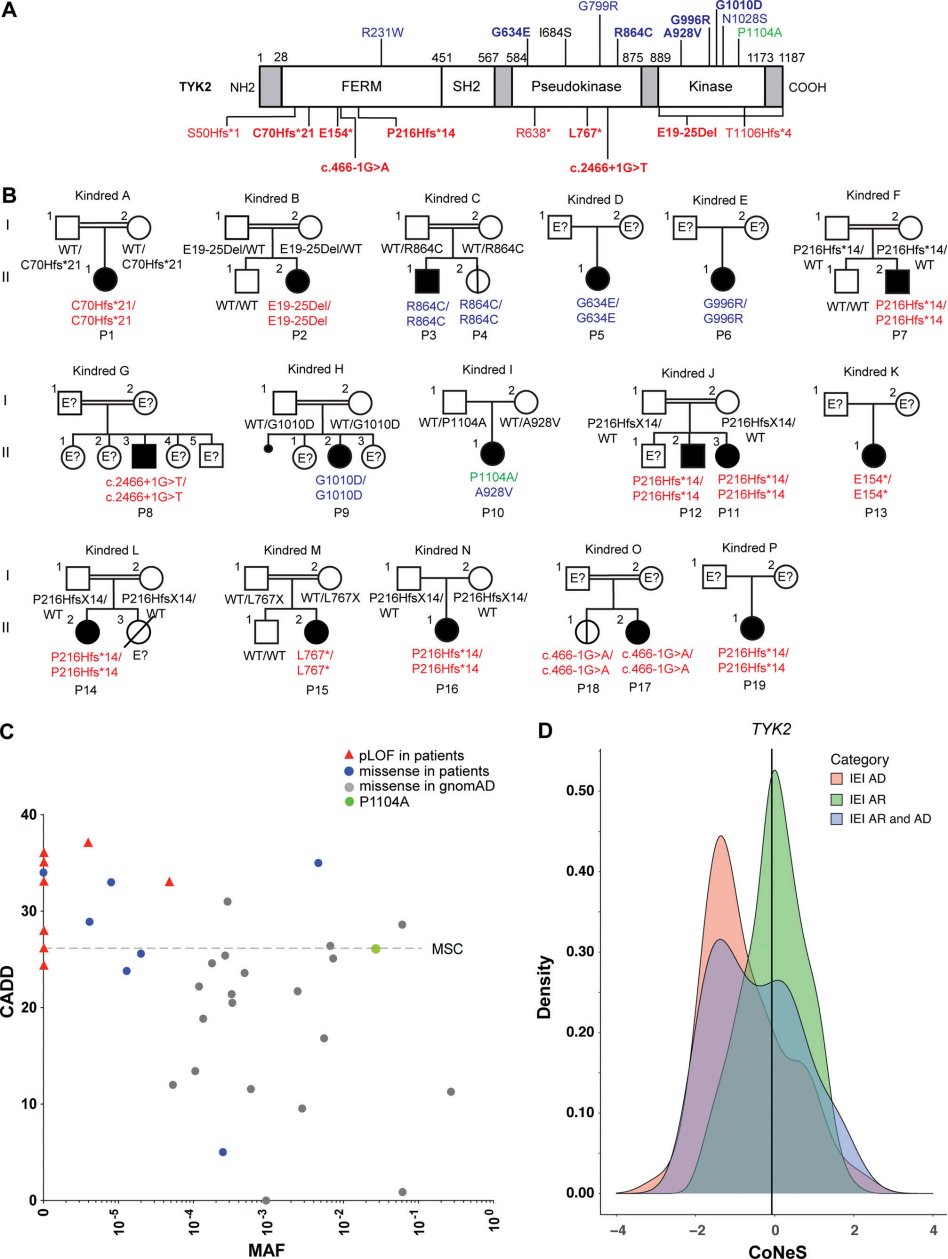
**Identification of AR TYK2 deficiency and familial segregation. (A)** Schematic representation of the *TYK2* coding sequence and protein domains. The locations of the mutations identified previously and in this report (bold) are indicated. Red indicates a predicted LOF variant, blue a rare missense variant, and green a common missense variant associated with susceptibility to TB. FERM, 4.1 protein, ezrin, radixin, and moesin. **(B)** Familial segregation of the mutations. Black indicates disease status. E?, genotype not available. **(C)** CADD minor allele frequency (MAF) graph displaying the frequency of the variants found in the homozygous state in gnomAD and in our patients relative to their deleteriousness. Already published variants are also shown. **(D)** Graphical representation of the CoNeS value for *TYK2*, in comparison with those for autosomal dominant (AD), recessive (AR), or both types of inborn errors of immunity (IEI).

Genetic and clinical characteristics of TYK2-deficient patients (previously reported and new patients). AV, adenovirus; MAF, minor allele frequency; MC, molluscum contagiosum. Patient number and mutations are as in Fig. 1. The frequency of the variant is annotated according to gnomAD data. TYK2 expression is indicated. The functional effect of the mutation is indicated: LOF or hypomorphic (hypo). The defect observed for each signaling pathway is annotated. N means normal. The defect of the IL-6 response observed only in P-Jap is highlighted in yellow. There are, therefore, five forms of AR TYK2 deficiency. In orange, AR complete TYK2 deficiency without TYK2 expression. In dark orange, AR complete TYK2 deficiency with residual TYK2 expression. In blue, AR partial TYK2 deficiency with normal TYK2 expression, affecting all signaling pathways. In green, AR partial TYK2 deficiency, selective for IL-23 due to a common variant. In dark green, AR partial TYK2 deficiency, selective for IL-23 due to a rare variant.

Homozygosity for the common *TYK2* missense variant P1104A has recently been described as a rare genetic etiology of MSMD and a common genetic etiology of primary TB (Boisson-Dupuis et al., 2018; Casanova and Abel, 2022. It has low penetrance for MSMD (probably <1%) and high penetrance for TB in endemic areas (probably >80%; Boisson-Dupuis et al., 2018; Kerner et al., 2021; Kerner et al., 2019). Odds ratios of ∼40 for MSMD and ∼90 for TB have been reported for patients living in endemic areas outside Europe, in which the frequency of P1104A homozygosity is between 1/10,000 and 1/1,000. Homozygosity for P1104A reaches a frequency of about 1/600 in individuals of European descent, in whom the prevalence of TB is currently low (Kerner et al., 2019). Nevertheless, it accounts for ∼1% of the cases of TB since World War II in Britons enrolled in the UK Biobank cohort (Kerner et al., 2019). The P1104A variant first appeared in the ancestors of West Eurasians ∼30,000 yr ago (Kerner et al., 2021). Its frequency has substantially decreased over the last 2,000 yr in Europe due to strong negative selection, probably due to TB endemicity (Kerner et al., 2021). The P1104A variant affects the enzymatic activity of TYK2 but has no impact on its scaffolding function or capacity to be phosphorylated as a substrate. Homozygosity for P1104A underlies mycobacterial disease by selectively disrupting cellular response to IL-23. Similarly, AR complete IL-23R deficiency underlies MSMD with incomplete penetrance (Martinez-Barricarte et al., 2018). Penetrance is probably higher in patients with AR TYK2 deficiency and impaired cellular responses to both IL-12 and IL-23, and even higher in patients with IL-12Rβ1 deficiency with abolished responses to both cytokines (Fieschi et al., 2003; de Beaucoudrey et al., 2010; Casanova and Abel, 2022). Two types of inherited TYK2 deficiencies are known: AR complete deficiency underlying MSMD (and more rarely TB) and/or viral diseases, and homozygosity for P1104A deficiency underlying TB (and more rarely MSMD) without viral diseases. We set out to discover new genetic and immunological forms of AR TYK2 deficiency by searching for biallelic *TYK2* variants in patients with mycobacterial or viral diseases.

## Results

### Rare *TYK2* variants identified by whole-exome sequencing (WES)

**Figure S1.**
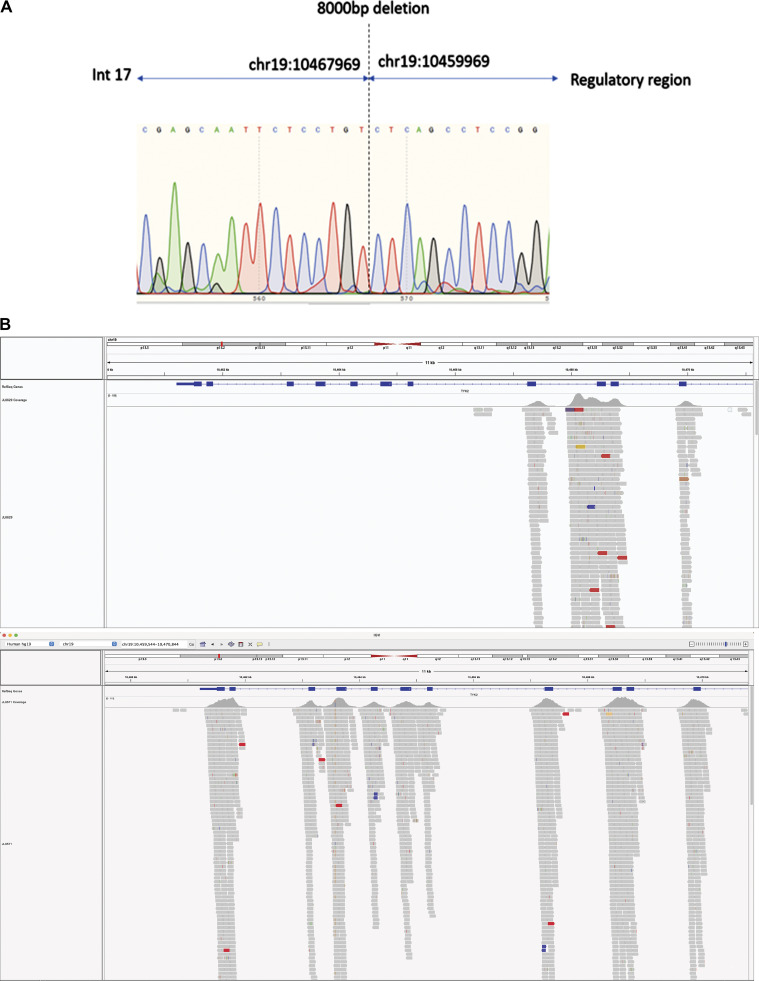
**Large deletion in P2. (A)** Sanger sequencing of the junction on chromosome 19 of P2. **(B)** Visualization in IGV of the large deletion in P2 (top) relative to a healthy individual (bottom).

We searched for biallelic variants, including at least one very rare or rare (frequencies <0.01 and <1%, respectively, in the general population) nonsynonymous or essential splice site variant of *TYK2* (NM_003331) by WES in patients with unexplained mycobacterial or viral diseases. We identified and characterized 19 patients (15 of whom were new patients, the remaining patients having been described clinically; Sarrafzadeh et al., 2020; Zhang et al., 2022) from 16 families (Fig. 1 and Table 1): P1 is homozygous for a previously reported 4-bp deletion in exon 4 (c.208_211:GCTTdel; p.C70Hfs*21; Minegishi et al., 2006); P2 is homozygous for a copy number variant consisting of a large deletion spanning exons 19–25 (g.10467969_10459969del; E19_25del; Fig. S1); P3 and his sister (P4) are homozygous for a substitution in exon 18 (c.2590 C>T; predicted p.R864C) previously reported in a patient compound heterozygous for this allele and p.N1028S (Guo et al., 2020); P5 is homozygous for a missense substitution in exon 13 (c.1901 G>A; p.G634E); P6 is homozygous for a missense substitution in exon 21 (c. 2986 G>C; p.G996R); P7, P11, P12, P14, P16, and P19 are homozygous for a single base-pair deletion in exon 7 (c.647delC; p.P216Hfs*14) already described elsewhere (Fuchs et al., 2016; Sarrafzadeh et al., 2020; Zhang et al., 2022); P8 is homozygous for an essential splice-site mutation at the end of exon 17 (c.2466+1G>T); P9 is homozygous for a missense variant in exon 22 (c.3029G>A; p.G1010D); P10 is compound heterozygous for two missense variants, one in exon 20 (g.2783C>T; p.A928V) and the other in exon 23, the common allele P1104A (Boisson-Dupuis et al., 2018); P13 is homozygous for a nonsense variant in exon 5 (c.460G>T; p.E154*; Kreins et al., 2015); P15 is homozygous for a 9-bp deletion in exon 16 (c.2303_2311del; p.L767*; Kreins et al., 2015); and P17 and P18 are homozygous for an essential splice-site mutation at the end of exon 5 (c.466-1G>A).

### Biallelic *TYK2* variants in 19 patients from 16 families

Homozygosity or compound heterozygosity in the patients was confirmed by Sanger sequencing. Familial segregation of the alleles was also consistent with an AR trait with high penetrance for at least one infectious phenotype per individual carrying a biallelic genotype. Only two young patients, P4 (p.R864C/p.R864C) and P18 (c.466-1G>A/c.466-1G>A), are currently asymptomatic. However, neither was vaccinated with the BCG vaccine. P4 (p.R864C/p.R864C) was not vaccinated due to the history of BCG disease in her brother, and P18 (c.466-1G>A/c.466-1G>A) is living in a country where BCG vaccination is not mandatory. These alleles are private (i.e., not found in public databases; Genome Aggregation Database [gnomAD] v2.1.1; e.g.E19_25del; p.P216Hfs*14; c.2466+1G>T; p.E154*; p.G1010D; and c.466-1G>A), rare (p.A928V: 4.63 × 10^−3^), very rare (p.C70Hfs*21: 4.77 × 10^−5^; p.R864C: 8.01 × 10^−6^, p.G634E: 2.47 × 10^−4^, p.G996R: 1.29 × 10^−5^, and p.L767*: 4.004 × 10^−6^), or common (p.P1104A: 2.69 × 10^−2^). The combined annotation-dependent depletion (CADD) scores of most of these variants were higher than the mutation significance cutoff (MSC) of 20.7 (Fig. 1 C; Itan et al., 2015, 2016; Zhang et al., 2018). The *TYK2* locus is subject to negative selection at an intensity consistent with AR inborn errors of immunity, according to the consensus-based measure of negative selection (CoNeS), a sequence-based metric for quantifying gene-level selection (Rapaport et al., 2021; Fig. 1 D). Homozygosity in more than one individual was found for 10 variants, including six rare and four common variants (including P1104A), in public databases. The patients included in our laboratory database of >15,000 exomes who are homozygous for any of these variants (including an update on P1104A) will be studied in another project. These findings suggest that these 19 patients from 16 families have known or novel forms of AR TYK2 deficiency.

### Clinical phenotype of 13 patients homozygous for predicted loss-of-function (pLOF) variants

The clinical phenotype of each of the 19 patients is summarized in Table 1 and detailed in Materials and methods. Nine of the 13 patients carried pLOF (including essential splice site, deletion/insertion leading to frameshift, and nonsense variants) variants that had been characterized before (Fuchs et al., 2016; Kreins et al., 2015; Minegishi et al., 2006). P1 (C70Hfs*21/C70Hfs*21) carries the same mutation as the first TYK2-deficient patient described (P-Jap; Minegishi et al., 2006) but suffered from viral diseases only, with no mycobacterial disease, despite having been vaccinated with BCG. He did not share the HIES phenotype of P-Jap (Minegishi et al., 2006). P2 (E19-25Del/E19-25Del) is homozygous for a newly identified large deletion that removes the last six exons of *TYK2.* She suffered from atopy, cellulitis, viral (HSV), mycobacterial (BCG-osis), and parasitic (*Leishmania major*) infections, with one episode of oral thrush due to *Candida albicans*. P7, P11, P12, P14, P16, and P19 carried the same *TYK2* genotype as another previously described patient (P-Ger; P216Hfs*14/P216Hfs*14; Fuchs et al., 2016). These six patients presented with mycobacterial or viral diseases or both, but did not have the high serum IgE levels of P-Ger. P8 (c.2466+1G>T/c.2466+1G>T), with a previously unknown essential splice-site mutation in *TYK2,* had respiratory infections for which the causal microbe was not identified, and no signs of HIES. P13 (E154*/E154*) and P15 (L767*/L767*) carried the same mutation as other previously described patients (Kreins et al., 2015). The previously reported patient, who was homozygous for L767*, will be referred to hereafter as P-Tur (Kreins et al., 2015). P13 (E154*/E154*) and P15 (L767*/L767*) display the same clinical phenotype, consisting of mycobacterial disease, including disseminated BCG disease (BCG-osis) and TB, with chronic mucocutaneous disease (due to *C. albicans*) for P13, but no severe viral disease in either patient. P17 (c.466-1G>A/c.466-1G>A) suffered from COVID-19 pneumonia, requiring hospitalization, whereas her older sister P18 is asymptomatic. P18 is 7 yr old and was not vaccinated with BCG. We herein described 13 patients with pLOF variants, including nine new patients. Among the latter, 33% suffered from mycobacterial diseases, 66% from viral diseases, and 33% from fungal diseases, whereas the percentages were 60, 53, and 6%, respectively, for the previously reported 15 patients (Fuchs et al., 2016; Guo et al., 2020; Kreins et al., 2015; Minegishi et al., 2006; Sarrafzadeh et al., 2020; Wu et al., 2020; Zhang et al., 2022). Interestingly, six of these 13 patients suffered from COVID-19 before vaccination (P7, P12, P14, P16, P17, and P19), including four with hypoxemic pneumonia (P7, P12, P16, and P19; Table 1).

### Clinical phenotype of six patients homozygous for in-frame variants

One of the four rare missense variants has already been reported but is not yet functionally characterized (Guo et al., 2020), whereas the common P1104A variant has been thoroughly studied (Boisson-Dupuis et al., 2018). Among the patients bearing one or two of these missense variants, P3 (R864C/R864C) had mycobacterial (BCG) infections and had one episode of oral thrush due to *C. albicans,* whereas his sister, P4 (R864C/R864C), who was not vaccinated with BCG, remains asymptomatic. P5 (G634E/G634E), P6 (G996R/G996R), and P10 (P1104A/A928V) did not develop viral diseases but suffered from infections with *Mycobacterium fortuitum*, *Nocardia*, and Enterobacteriaceae (P5: G634E/G634E), or *M.tb* (P6: G996R/G996R and P10: P1104A/A928V). Susceptibility to these pathogens is well characterized in MSMD patients, particularly those with IL-12Rβ1 or IL-12p40 deficiencies (Bustamante et al., 2014). P9 (G1010D/G1010D) suffered from meningeal and respiratory disease caused by unidentified pathogens. None of the patients with in-frame or pLOF *TYK2* variants had high serum levels of IgE. Four of these 19 patients with biallelic *TYK2* variants presented only intramacrophagic infections, six had viral diseases only, seven had combinations of viral, mycobacterial, and fungal diseases, and two were asymptomatic. These phenotypes were not tightly correlated with the two types of *TYK2* genotype (pLOF versus in-frame), suggesting that the penetrance for each infectious phenotype may be incomplete, or that these two categories of genotype may not entirely reflect the biochemical impact of the *TYK2* variants.

### Expression, auto- and transphosphorylation, and scaffolding of mutant TYK2 proteins

**Figure S2.**
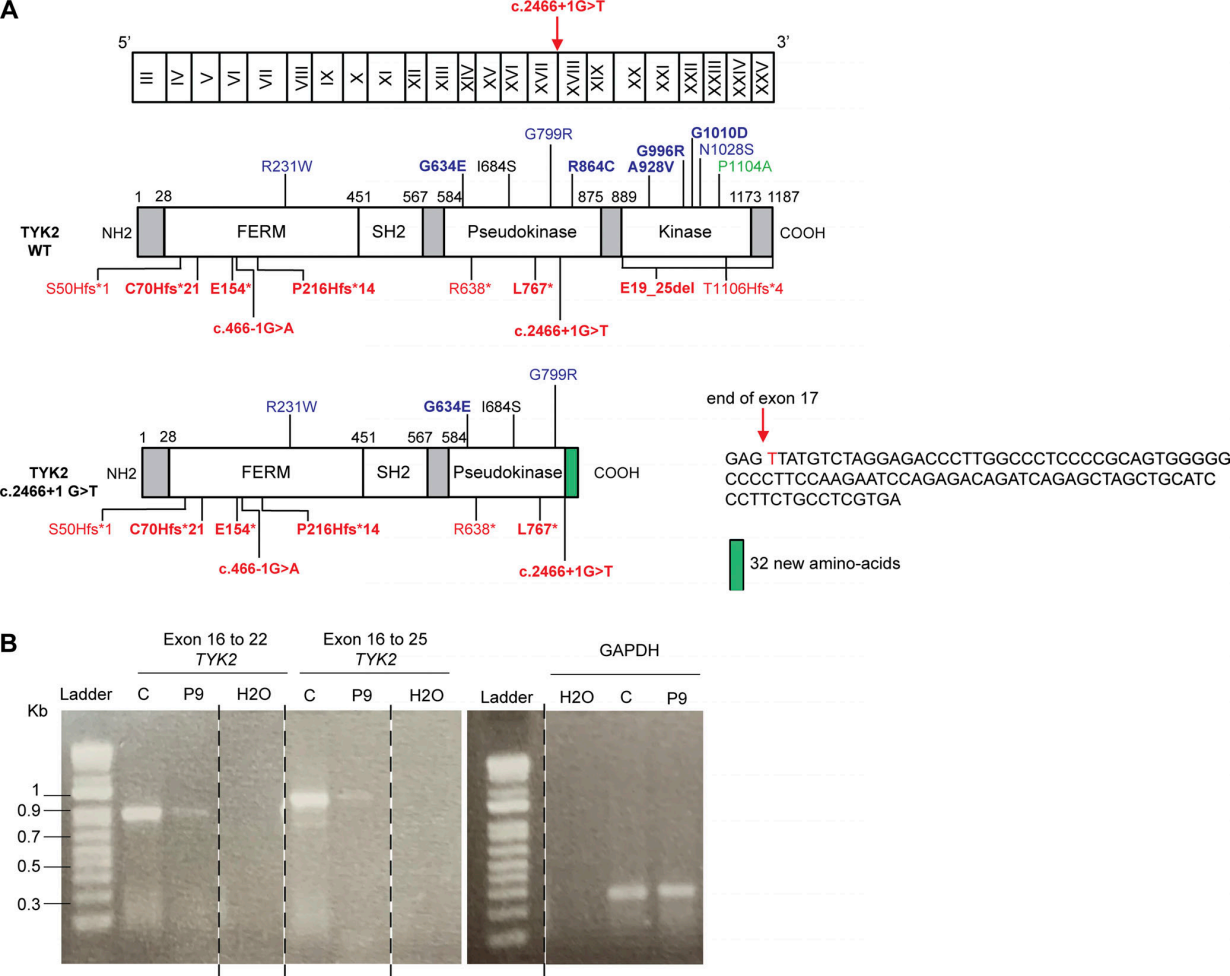
**Characterization of the mutations of P8 and P9. (A)** Schematic representation of the *TYK2* cDNA with exons annotated (top), the TYK2 protein with all the mutated residues identified (middle), and a representation of the protein of P8 (bottom left) and the nucleotide sequence found in the cells of the patient (bottom right). **(B)** Amplification of the *TYK2* cDNA from P9, revealing the presence of a single band of the same size as the WT protein, but of lower abundance, probably due to mRNA decay. Source data are available for this figure: SourceData FS2.

**Figure 2.**
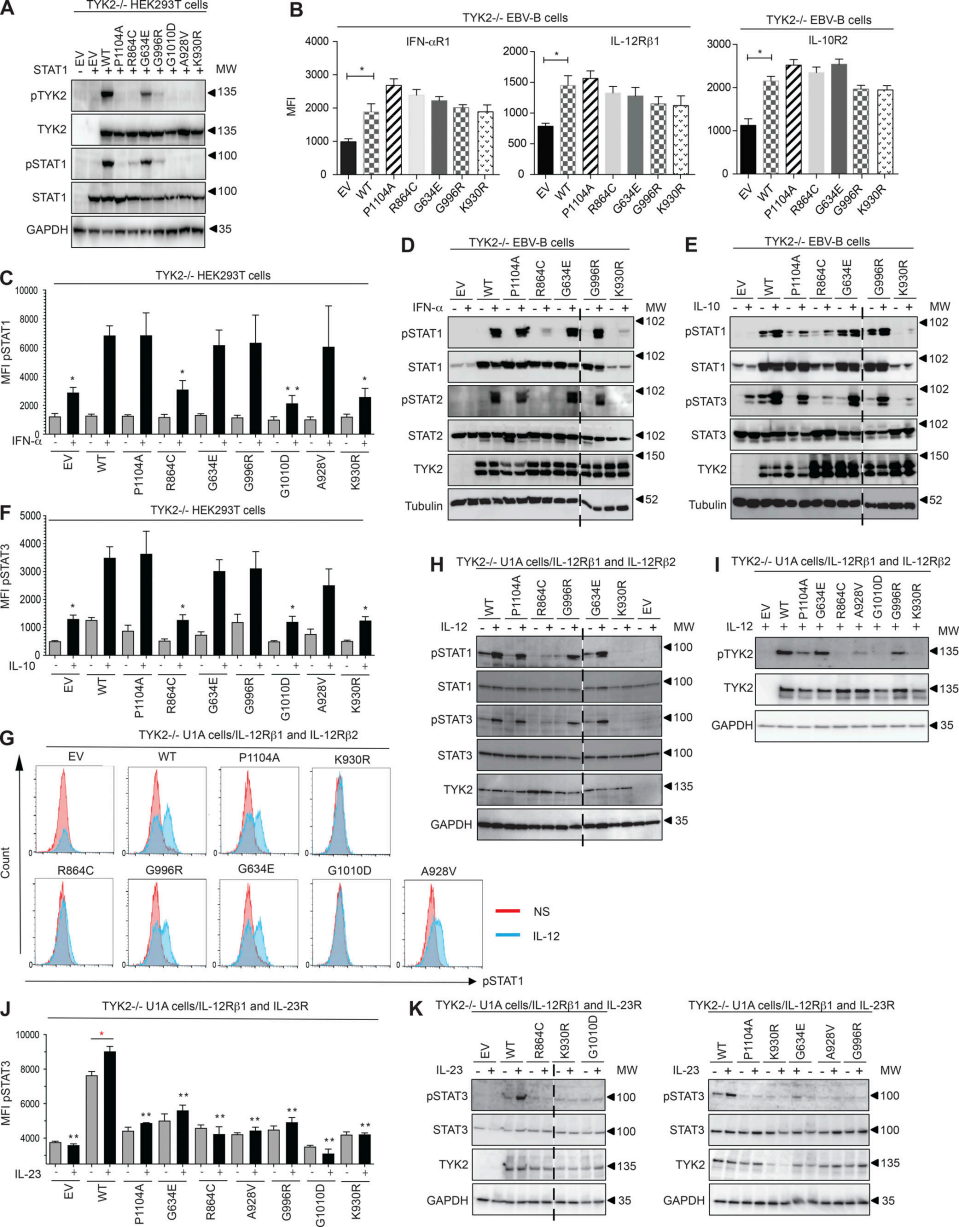
**Functional characterization of the mutant *TYK2* alleles in overexpression conditions. (A)** Western blot analysis of the expression capacity of the *TYK2* alleles and of the capacity of the resulting proteins for auto- and trans-phosphorylation in TYK2-deficient HEK293T cells. Where indicated, the cells were transiently transfected with *STAT1*. The experiment shown is representative of at least two independent experiments performed. **(B)** IFN-αR1, IL-12Rβ1, and IL-10R2 expression in reconstituted EBV-B cells with the *TYK2* alleles indicated, as determined by flow cytometry. *, P < 0.05, two-tailed Student’s *t* tests. Nonsignificant values are not indicated. Two (IFN-αR1, IL-12Rβ1) and three (IL-10R2) independent experiments were performed. MFI, mean fluorescence intensity. **(C and D)** Response to IFN-α of transfected TYK2-deficient HEK293T (C) and reconstituted EBV-B cells (D) with the *TYK2* alleles indicated, as determined by flow cytometry and Western blotting, respectively. The data shown are representative of at least two independent experiments. *, P < 0.05; **, P < 0.01, two-tailed Student’s *t* tests between the stimulated condition of the *TYK2* WT allele and the other stimulated conditions. Nonsignificant values are not indicated. **(E and F)** Response to IL-10 of reconstituted EBV-B cells (E) and TYK2-deficient HEK293T cells (F) with the *TYK2* alleles indicated, as determined by Western blotting and flow cytometry, respectively. The data shown are representative of at least two independent experiments. *, P < 0.05, two-tailed Student’s *t* tests between the stimulated condition of the *TYK2* WT allele and the other stimulated conditions. Nonsignificant values are not indicated. **(G and H)** Response to IL-12 of TYK2-deficient U1A cells stably transfected with IL-12Rβ1 and IL-12Rβ2 and with the *TYK2* alleles indicated, as determined by flow cytometry (G) and Western blotting (H). The data shown are representative of at least two independent experiments. **(I)** Phosphorylation of TYK2, as a substrate, in TYK2-deficient U1A cells stably transfected with IL-12Rβ1 and IL-12Rβ2, and transfected with the *TYK2* alleles indicated, as determined by Western blotting with a specific anti-phosphoTYK2 antibody after stimulation with IL-12. The data shown are representative of at least two independent experiments. **(J and K)** Response to IL-23 of TYK2-deficient U1A cells stably transfected with IL-12Rβ1 and IL-23R transfected with the *TYK2* alleles indicated, as determined by flow cytometry (J) and Western blotting (K) for pSTAT3 and pSTAT1. The data shown are representative of at least two independent experiments. *, P < 0.05; **, P < 0.01, two-tailed Student’s *t* tests between the stimulated condition of the *TYK2* WT allele and the other stimulated conditions (in black) and between the nonstimulated and stimulated condition (in red). Nonsignificant values are not indicated. MW, molecular weight in kD. Source data are available for this figure: SourceData F2.

We evaluated the impact of each variant on TYK2 protein levels. Four alleles had already been shown to be loss-of-expression (LOE) and LOF (L767*, C70Hfs*21, P216Hfs*14, and E154*; Fuchs et al., 2016; Kreins et al., 2015; Minegishi et al., 2006; Sarrafzadeh et al., 2020). Three other pLOF variants, the large deletion (g.10467969_10459969del; E19_25del) and the essential splice-site mutants (c.2466+1G->T and c.466-1G>A), were investigated directly in the patients’ cells (see below; Figs. S1 and S2). The other five alleles were missense alleles. We used a WT *TYK2* allele as the substrate for site-directed mutagenesis to generate each missense variant: R864C, G634E, G996R, G1010D, and A928V. We used the kinase-dead alleles P1104A and K930R as negative controls for the catalytic activity of TYK2, as previously reported (Boisson-Dupuis et al., 2018). Unlike K930R, which is not only kinase-dead but also phosphorylation incompetent, P1104A is a functional substrate. We transiently transfected CRISPR-generated TYK2-deficient HEK293T cells with each of the five mutant alleles and analyzed the production and auto- and transphosphorylation of TYK2 and STAT1 by Western blotting (Fig. 2 A). We found that five protein variants (R864C, G634E, G996R, G1010D, and A928V) were produced in normal amounts, but four of these five protein variants (R864C, G996R, G1010D, and A928V) had little or no detectable autocatalytic activity (the remaining variant, G634E, being hypomorphic for this function; Fig. 2 A). Moreover, these five variants displayed abolished or severely reduced transphosphorylation of the substrate STAT1 relative to the WT protein (Fig. 2 A). TYK2 deficiency is known to reduce the stability of the surface receptors IFN-αR1, IL-12Rβ1, and IL-10R2, by disrupting TYK2-dependent scaffolding functions. EBV-immortalized B (EBV-B) cells derived from a TYK2-deficient patient with no TYK2 protein production were stably transduced with a retrovirus generated with an empty vector (EV) or a vector containing the WT, R864C, G634E, G996R, P1104A, or K930R cDNA (Boisson-Dupuis et al., 2018; Martinez-Barricarte et al., 2016). Transduction with each of the alleles tested restored the scaffolding-dependent surface expression of IFN-αR1, IL-12Rβ1, and IL-10R2 (Fig. 2 B). These results suggest that, when overexpressed, the five TYK2 missense proteins—R864C, G634E, G996R, G1010D, and A928V—are produced in normal amounts and perform their role in scaffolding, but have lower levels of auto- and transphosphorylation activities than their WT counterpart.

### Responses to IFN-α, IL-10, IL-12, and IL-23 in cell lines overexpressing mutant *TYK2*

The function of the five missense alleles was then analyzed in cell lines: a TYK2-deficient HEK293T cell line for the IFN-α and IL-10 signaling pathways and a TYK2-deficient fibrosarcoma cell line stably expressing the IL-12R heterodimer (IL-12Rβ1 and IL-12Rβ2) or IL-23R (IL-12Rβ1 and IL-23R) for IL-12 and IL-23 signaling, respectively. In addition, the R864C, G634E, and G996R *TYK2* alleles were analyzed in a TYK2-deficient EBV-B cell line for the IFN-α and IL-10 pathway. We studied the responses to IFN-α, IL-10, IL-12, and IL-23 by Western blotting or flow cytometry, with K930R as a negative control (Kreins et al., 2015). Three of the variant proteins responded to IFN-α like the WT protein, whereas R864C and G1010D displayed impaired STAT1 phosphorylation in response to IFN-α (Fig. 2 C). Western blotting revealed higher levels of STAT1 phosphorylation for R864C than for K930R (Fig. 2 D). The phosphorylation of STAT3 in response to IL-10 in EBV-B and TYK2-deficient HEK293T cells transiently expressing IL-10R1 and IL-10R2 was normal for three of the missense proteins, the exceptions again being R864C and G1010D, which behaved like K930R (Fig. 2, E and F). We used TYK2-deficient fibrosarcoma (U1A) cells stably expressing IL-12Rβ2 and IL-12Rβ1 to test the impact of the *TYK2* variants on the IL-12 response pathway. STAT1 phosphorylation, as detected by intracellular flow cytometry, was abolished by the G1010D protein (Fig. 2 G) but was impaired without total abolition by the R864C protein, as shown by the Western blot in Fig. 2 H. The other variants (P1104A, G996R, A928V, and G634E) behaved normally (Fig. 2, G and H). We assessed the substrate capacity of each variant in U1A-expressing IL-12Rβ1 and β2 cells. We found that, like P1104A, G634E, A928V, and G996R were phosphorylated in response to IL-12 stimulation, whereas G1010D and R864C, like K930R, were not (Fig. 2 I). Like P1104A, all the missense proteins resulted in impaired responses to IL-23 in TYK2-deficient fibrosarcoma (U1A) cells stably expressing IL-12Rβ1 and IL-23R (Fig. 2, J and K). Collectively, these data show that the *TYK2* alleles G1010D and R864C are LOF and severely hypomorphic (e.g., with a marked reduction, but not a complete abolition, of function), respectively, in response to all known TYK2-dependent cytokines, through a mechanism probably similar to that of K930R. By contrast, G996R, A928V, and G634E, like the P1104A common variant, selectively impair IL-23 signaling, at least in these conditions of overexpression.

### Production and role in scaffolding of TYK2 variants in the EBV-B cells of patients with *TYK2* mutations

**Figure 3.**
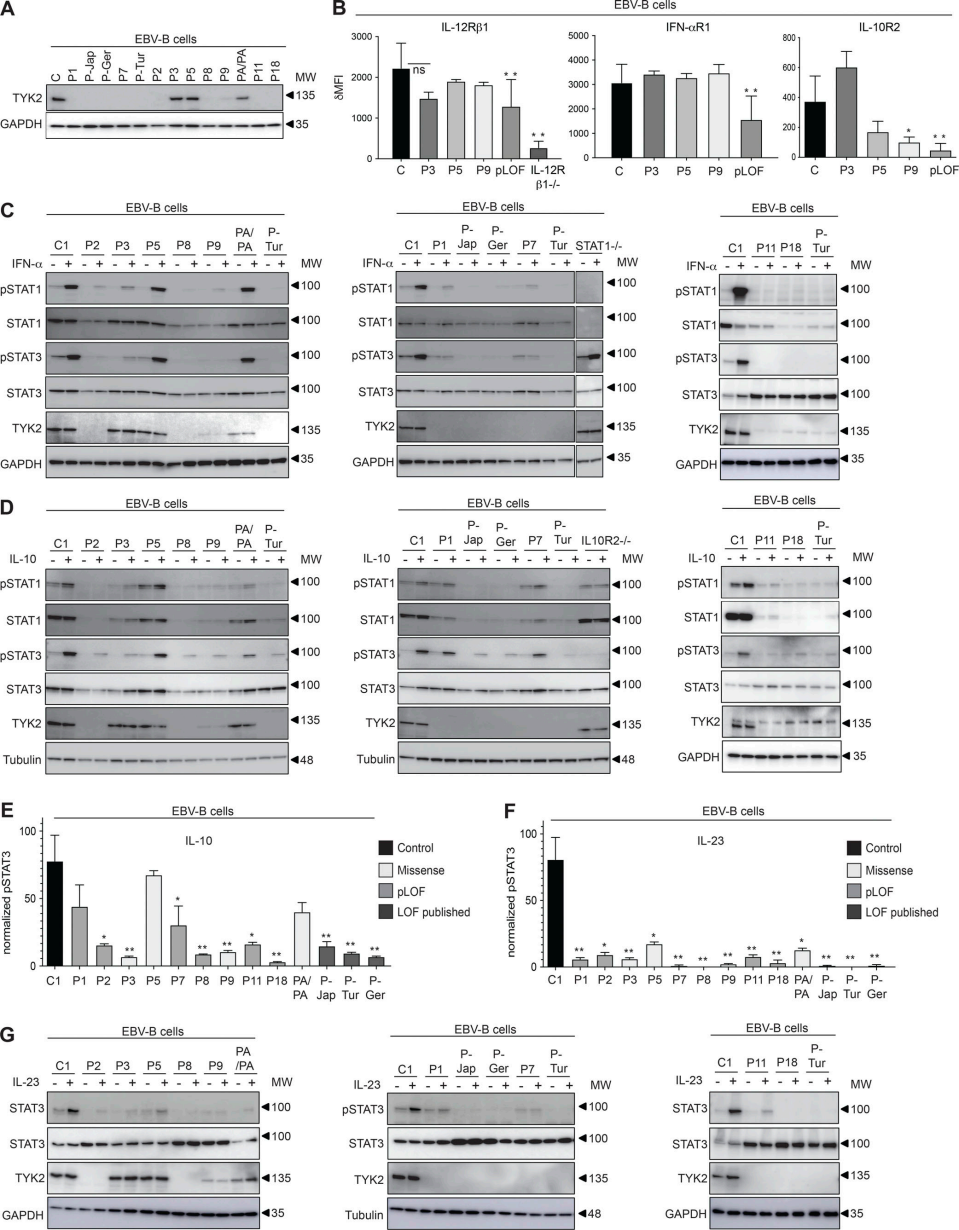
**Functional characterization of the patients’ cell lines. (A)** TYK2 levels, assessed by Western blotting of EBV-B cells from the patients. The data shown are representative of at least two independent experiments. **(B)** Expression of IL-12Rβ1, IFN-αR1, and IL-10R2 in EBV-B cells from the patients indicated, as determined by flow cytometry. pLOF cells comprise cells from P1, P-Jap, P-Tur, P-Ger, P7, P2, and P8. *, P < 0.05; **, P < 0.01, two-tailed Student’s *t* tests. Nonsignificant values are not indicated. Two to three independent experiments were performed. MFI, mean fluorescence intensity. **(C–G)** Response to IFN-α (C) IL-10 (D and E), and IL-23 (F and G) of EBV-B cells from the patients, as determined by Western blotting (C, D, and G) and flow cytometry (E and F), assessing STAT phosphorylation. TYK2 levels were determined by Western blotting of the patients’ EBV-B cells. *, P < 0.05; **, P < 0.01, two-tailed Student’s *t* tests between the stimulated condition of the controls and the other stimulated conditions. Nonsignificant values are not indicated. Data representative of at least two independent experiments are shown. MW, molecular weight in kD. Source data are available for this figure: SourceData F3.

We assessed TYK2 production by immunoblotting in EBV-B cells from the patients. No EBV-B cells were available for P4, P6, P10, P12–P17, or P19. P-Jap carries C70Hfs*21 (Minegishi et al., 2006), P-Ger carries P216Hfs*16 (Fuchs et al., 2016), and P-Tur carries L767* (Kilic et al., 2012; Kreins et al., 2015). TYK2 protein levels were similar in patients homozygous for R864C (P3), G634E (P5), a patient homozygous for P1104A (PA/PA; Boisson-Dupuis et al., 2018), and healthy control cell lines (Fig. 3 A). By contrast, TYK2 was undetectable in cells homozygous for pLOF variants from P1 (C70Hfs*21/C70Hfs*21), P2 (E19_25del/E19_25del), P7 (P216fs*14/P216fs*14), P8 (c.2466+1G>T/c.2466+1G>T), P11 (P216fs*14/P216fs*14), and P18 (c.466-1G>A/c.466-1G>A), as in previously characterized cells from P-Jap, P-Tur, and P-Ger (Fig. 3 A). In addition, TYK2 production was almost completely abolished in P9 (G1010D/G1010D; Fig. 3 A). We assessed the scaffolding role of TYK2 by analyzing the expression of the various cytokine receptors associated with TYK2 (IL-12Rβ1, IFN-αR1, and IL-10R2) by flow cytometry. As previously shown (Boisson-Dupuis et al., 2018; Kreins et al., 2015; Ragimbeau et al., 2003), the surface expression of these receptors was diminished on EBV-B cells from patients with pLOF variants (P1: C70Hfs*21/C70Hfs*21, P2: E19_25del/E19_25del, P7: P216Rfs*14/P216Rfs*14, P8: c.2466+1G>T/c.2466+1G>T, P-Jap: C70Hfs*21/C70Hfs*21, P-Tur: L767*/L767*, and P-Germ: P216Rfs*14/P216RfsX14) than on healthy control cells (Fig. 3 B). The cells of P3 (R864C/R864C) and P5 (G634E/G634E) displayed normal levels of expression for IFN-αR1, IL-12Rβ1, and IL-10R2. The cell surface expression of IL-10R2 was weak in cells from P9 (G1010D/G1010D), whereas the expression of IFN-αR1 and IL-12Rβ1 was intact, probably due to the residual TYK2 expression of P9. Overall, our data show that all the pLOF alleles (C70Hfs*21, p216Hfs*16, L767*, E154*, E19_25del, and c.2466+1G>T) are LOE in the patients’ cells, abolishing the scaffolding function of TYK2. The missense variants, R864C and G634E, were produced in normal amounts and exerted their scaffolding properties, which may also have been the case for G996R and A928V, which we were unable to test due to an absence of material from the patients, and G1010D, which is LOF when overexpressed, impairs endogenous TYK2 expression but retains its scaffolding properties for IL-12Rβ1 and IFN-αR1, but not IL-10R2.

### Response to IFN-α, IL-10, and IL-23 in EBV-B cells from patients with *TYK2* mutations

We analyzed TYK2-mediated cellular responses to three cytokines with receptors expressed on EBV-B cells, comparing these responses to those of previously reported patients with the same *TYK2* genotype. We stimulated EBV-B cells from patients and controls with recombinant IFN-α, IL-23, or IL-10 and assessed the phosphorylation of STAT molecules (STATs). As already reported for the published TYK2-deficient patients with LOE variants (P-Tur: L767*/L767*, P-Ger: P216Rfs*14/P216Rfs*14, P-Jap: C70Hfs*21/C70Hfs*21), cells from P1 (C70Hfs*21/C70Hfs*21), P2 (E19_25del/E19_25del), P7 (P216Rfs*14/P216Rfs*14), P8 (c.2466+1G>T/c.2466+1G>T), P9 (G1010D/G1010D), P11 (P216 fs*14/P216 fs*14), and P18 (c.466-1G>A/c.466-1G>A) displayed an impaired response to IFN-α, whereas cells from P5 (G634E/G634E) responded normally to IFN-α (Fig. 3 C). Consistent with the overexpression data, levels of STAT1 and STAT3 phosphorylation in P3 (R864C/R864C) were intermediate between those in control cells and cells with complete TYK2 deficiency (Fig. 3 C). As previously observed for the published cases (Kreins et al., 2015), levels of STAT3 and STAT1 phosphorylation after IL-10 stimulation were lower than normal, to various degrees, in cells from P1 (C70Hfs*21/C70Hfs*21), P2 (E19_25del/E19_25del), P3 (R864C/R864C), P7 (P216Rfs*14/P216Rfs*14), P8 (c.2466+1G>T/c.2466+1G>T), P9 (G1010D/G1010D), P11 (P216 fs*14/P216 fs*14), and P18 (c.466-1G>A/c.466-1G>A), probably due to their lower levels of IL-10R2 expression at the cell surface, whereas a normal response was observed for P5 (G634E/G634E; Fig. 3, D and E). Furthermore, STAT3 phosphorylation in response to IL-23 stimulation was impaired in the EBV-B cells of all patients, regardless of TYK2 levels (Fig. 3, F and G). Thus, cells from all patients homozygous for LOE alleles displayed an impaired response to IFN-α, IL-10, and IL-23, consistent with previous findings for patients with complete TYK2 deficiency (Kreins et al., 2015; Minegishi et al., 2006). P9 (G1010D/G1010D) is the first patient with complete TYK2 functional deficiency reported to have cells displaying residual TYK2 expression. Cells from P5 (G634E/G634E) behaved like cells homozygous for P1104A, with a specific defect of IL-23 signaling (Boisson-Dupuis et al., 2018). Finally, cells from P3 and P4 (R864C/R864C), homozygous for a hypomorphic *TYK2* allele, displayed a partial form of AR TYK2 deficiency across TYK2-dependent pathways.

**Figure 4.**
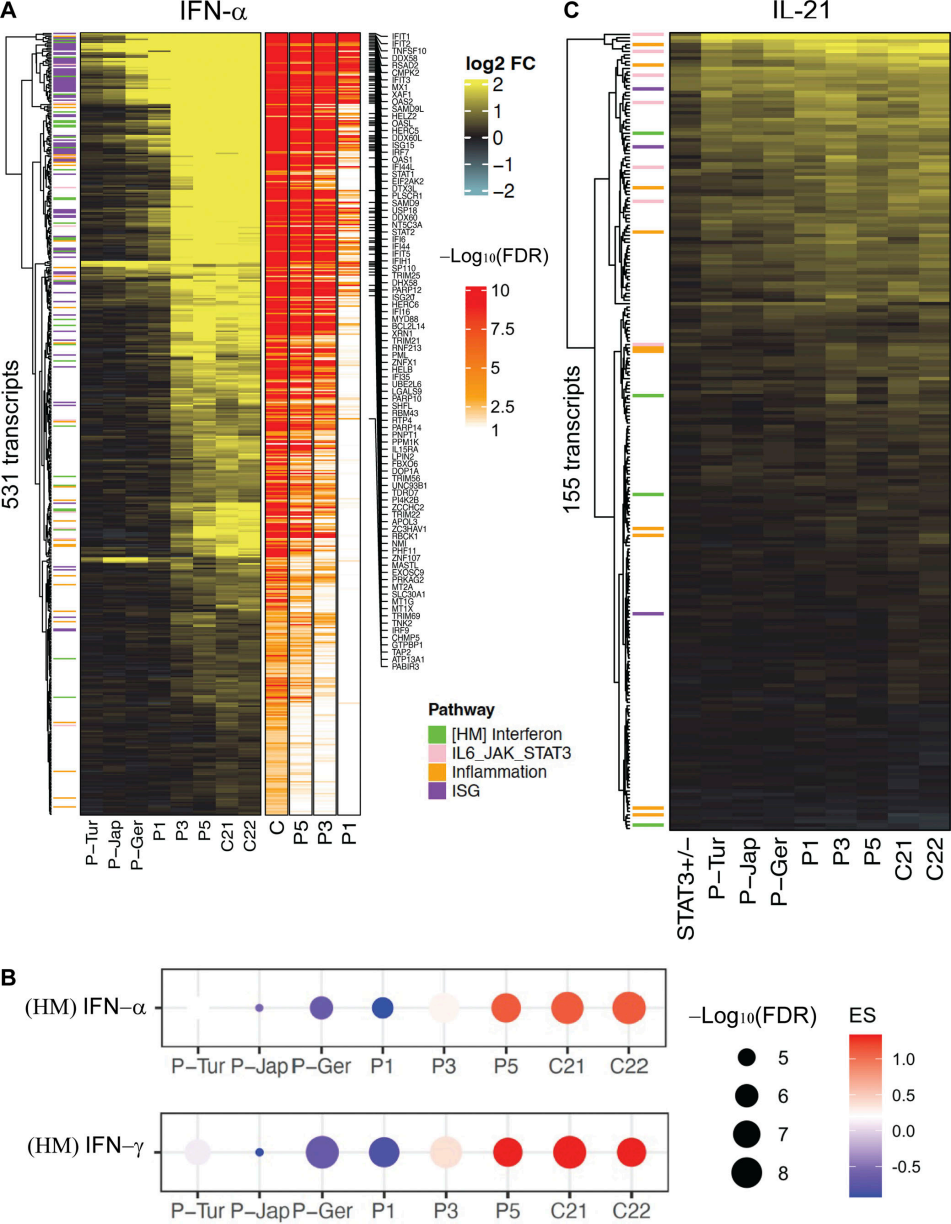
**Induction of target genes in the patients’ EBV-B cells.** RNA-seq analysis of EBV-B cells stimulated with IFN-α (10^5^ IU/ml) and IL-21 (100 ng/ml) for 2 h. Data normalization using negative binomial distribution (DESeq2 package). Benjamini–Hochberg FDR and log_2_ fold-change are represented. Each condition was duplicated, and then the mean of gene expression level was used for downstream analysis. **(A)** Heatmap includes 531 genes with relative fold-change >2 (FDR <0.05) in response to IFN-α treatment relative to NS samples in the control group (C21, C22). ISGs are indicated. **(B)** Dot plot representing IFN-α and IFN-γ enrichment scores (ES) between patients (P1, P3, and P5) and controls (C21 and C22). ES is represented by a spot of color, with red meaning increased abundance, and blue, decreased abundance. The degree of intensity of the spots denotes the levels of ES. The size of the spot represents FDR. HM, Hallmark gene sets. **(C)** Visualization of the DEGs between patients (P1, P3, and P5) and controls after IL-21 stimulation.

### Homozygosity for R864C *TYK2* defines a partial deficiency of the TYK2-dependent response to cytokines

**Figure S3.**
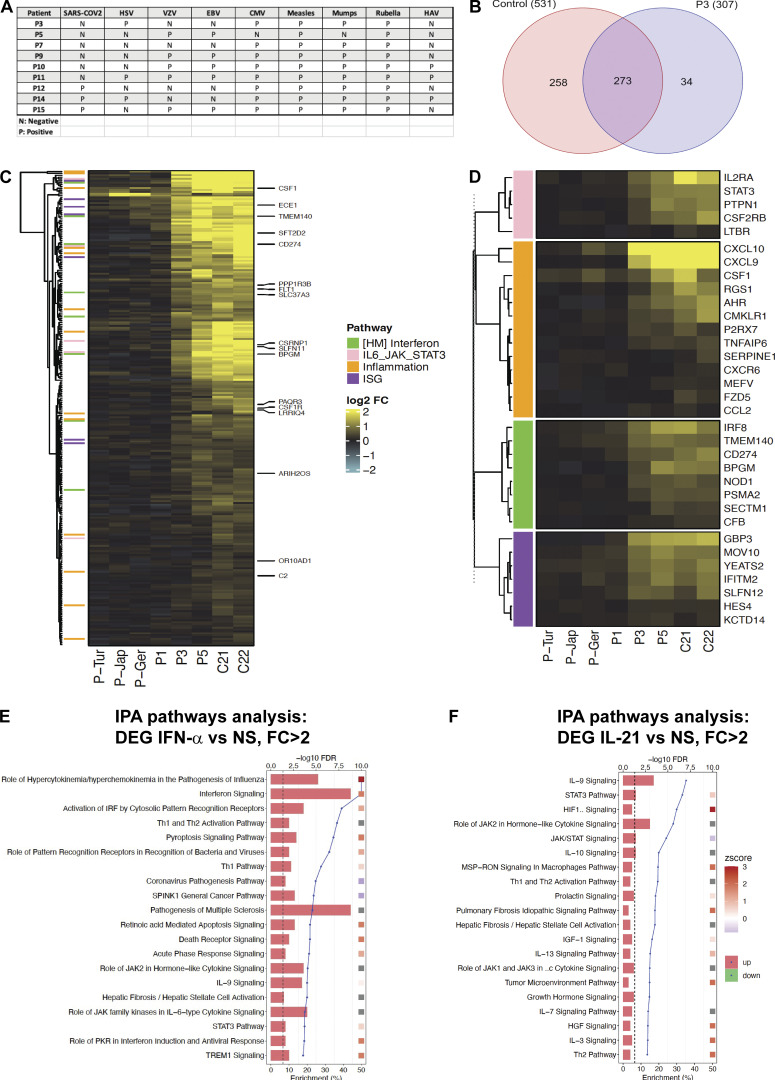
**Serological tests on the available serum samples from TYK2-deficient patients and IFN-α–induced genes in EBV B cells from P3 (R864C/R864C). (A)** Available serologies of TYK2-deficient patients. **(B)** Venn diagram of DEGs (IFN-α vs. NS) in controls and P3. **(C)** Heatmap of gene significantly induced by IFN-α in controls but not in P3 (R864C/R864C). **(D)** Heatmap of selected gene related to ISG, IFN, and inflammation. **(E and F)** DEGs between IFN-α vs. nonstimulation (E) and IL-21 vs. nonstimulation (F) samples. Top 20 canonical pathways ranking modulated by treatment identified using IPA analysis according to significance level (log_2_ fold-change, FDR < 0.05).

We investigated whether the partial response observed on STAT1 phosphorylation immunoblots of cells from P3 (R864C/R864C) stimulated with IFN-α affected the induction of transcription in response to IFN-α stimulation for IFN-stimulated genes (ISGs). We performed RNA sequencing (RNA-seq) to evaluate the transcriptomic response to IFN-α in EBV-B cells from P3 (R864C/R864C) and compared it with those of P1 (C70Hfs*21/C70Hfs*21), P5 (G634E/G634E), P-Tur (L767*/L767*), P-Jap (C70Hfs*21/C70Hfs*21), P-Ger (P216Rfs*14/P216Rfs*14), and healthy controls (Fig. 4 and S3, B and C). EBV-B cells were stimulated for 2 h with IFN-α. IL-21 was used as a positive control. In comparing the differentially expressed genes (DEGs) between the IFN-α stimulated and nonstimulated (NS) cells, we found 531 DEGs (false discovery rate [FDR] <0.05, fold-change >2) in the healthy controls (Fig. 4 A and Table S1), 63 DEGs in P1 (C70Hfs*21/C70Hfs*21), 307 DEGs in P3 (R864C/R864C), and 459 DEGs in P5 (G634E/G634E). The functional interpretation of the 531 transcripts (IFN-α vs. NS) using Ingenuity Pathway Analysis (IPA) demonstrated a strong enrichment of genes involved in the IFN signaling, which displayed the highest proportion of dysregulated transcripts among the top 20 differentially modulated IPA canonical pathways (Fig. S3 E). The impact of TYK2 deficiency in the IFN network was dissected by performing gene set enrichment analysis (GSEA: fgsea) using hallmark gene sets (http://www.gsea-msigdb.org/) and a threshold of log_2_ fold-change (531 DEGs: IFN-α vs. NS) of individual samples. We found that enrichment scores of the control samples were significantly (FDR <0.05) enriched in IFN-α– and IFN-γ–related pathways. The enrichment score was low for the AR complete TYK2-deficient patients (P-Tur [L767*/L767*], P-Jap [C70Hfs*21/C70Hfs*21], P-Ger [P216Rfs*14/P216Rfs*14], and P1 [C70Hfs*21/C70Hfs*21]), similar between the healthy controls and P5 (G634E/G634E), and intermediate between the healthy controls and the complete TYK2-deficient patients for P3 (Fig. 4 B). Interestingly, of the 77 canonical ISGs induced in controls (list of 146 ISGs from PMID: 34429372), 71 were similarly induced in P3 (R864C/R864C), likely explaining the absence of viral disease in this patient (Fig. S3 D). By contrast, transcriptomic changes in response to IL-21 (155 genes) were similar in all patients and controls (Fig. 4 C and S3 F). Thus, P3 (R864C/R864C) has a novel form of AR partial TYK2 deficiency, with detectable levels of TYK2 protein and an impaired response to all TYK2-dependent cytokines, including type I IFNs. However, this defect is milder than that seen in patients with complete TYK2 deficiency with or without protein production.

### Response to other potential TYK2-dependent cytokines in EBV-B cells from TYK2-deficient patients and TYK2-deficient HEK293T cells

**Figure 5.**
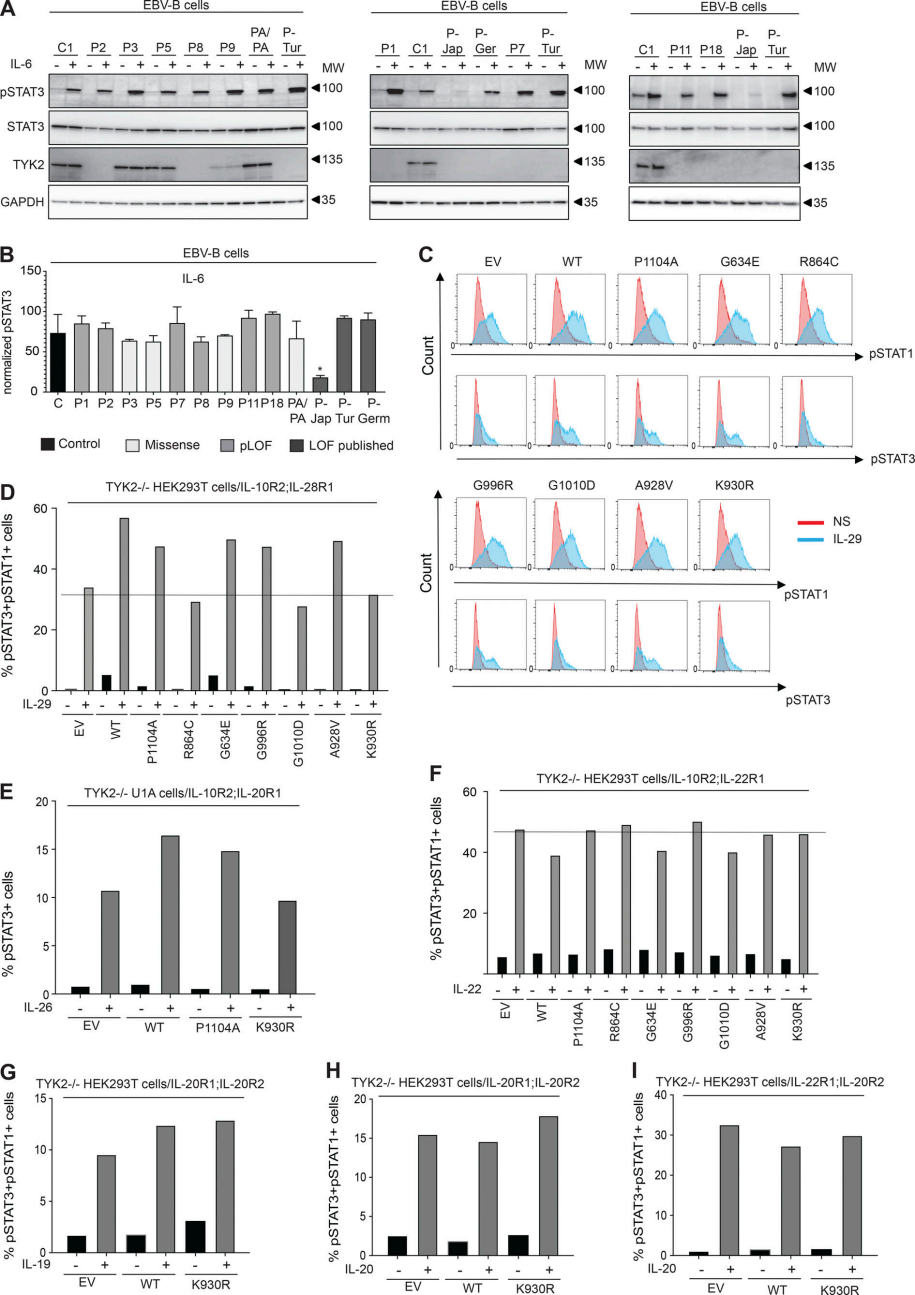
**TYK2-independent signaling pathways. (A and B)** Response to IL-6 in EBV-B cells from TYK2-deficient patients, as assessed by Western blotting (A) and flow cytometry (B) with an anti-pSTAT3 antibody. Data representative of at least two independent experiments are shown. *, P < 0.05; two-tailed Student’s *t* tests between the stimulated condition of the controls and the other stimulated conditions. Nonsignificant values are not indicated. **(C and D)** Response to IL-29 in TYK2-deficient HEK293T cells transiently transfected with IL-28R and IL-10R2, and the various *TYK2* alleles, as assessed by flow cytometry with specific labeled anti-pSTAT1 and anti-pSTAT3 antibodies. Data representative of at least two independent experiments are shown. **(E)** Diagram of the response to IL-26 in TYK2-deficient U1 cells transiently transfected with IL-10R2 and IL-20R1 and the various *TYK2* alleles, as measured by flow cytometry. Data representative of at least two independent experiments are shown. **(F)** Diagram of the response to IL-22 in TYK2-deficient HEK293T cells transiently transfected with IL-10R2 and IL-22R1, and the various *TYK2* alleles, as assessed by flow cytometry. Data representative of at least two independent experiments are shown. **(G–I)** Diagram of the responses to IL-19 (G) and IL-20 (H and I) in TYK2-deficient HEK293T cells transiently transfected with IL-20R1/IL-20R2, IL-22R1 and IL-20R2, and the various *TYK2* alleles, as assessed by flow cytometry. Data representative of at least two independent experiments are shown. MW, molecular weight in kD. Source data are available for this figure: SourceData F5.

The first patient with the homozygous C70Hfs*21 mutation (P-Jap) described had HIES, and his cells responded poorly to IL-6 (Minegishi et al., 2006). We therefore evaluated the IL-6 signaling pathway in all the available EBV-B cells from our patients (P1: C70Hfs*21/C70Hfs*21, P2: E19_25del/E19_25del, P3: R864C/R864C, P5: G634E/G634E, P7: P216Rfs*14/P216Rfs*14, P8: c.2466+1G>T/c.2466+1G>T, P9: G1010D/G1010D, P11: P216Rfs*14/P216Rfs*14, and P18: c.466-1G>A/c.466-1G>A) and from P-Tur (L767*/L767*) and P-Ger (P216Rfs*14/P216Rfs*14). STAT3 phosphorylation in response to IL-6, as determined by immunoblotting and intracellular flow cytometry, was similar to that in control cells for all TYK2-deficient cell lines—including those carrying the homozygous missense and pLOF variants—other than that for P-Jap, the first TYK2-deficient patient with HIES (Fig. 5, A and B). Remarkably, P1 and P-Jap are homozygous for the same *TYK2* variant (C70Hfs*21/C70Hfs*21). We previously showed that the impaired response to IL-6 in P-Jap (C70Hfs*21/C70Hfs*21) was TYK2 independent (Kreins et al., 2015). The impaired response to IL-6 in P-Jap (C70Hfs*21/C70Hfs*21) probably accounts for his HIES phenotype, the mechanism of which remains unknown (Minegishi et al., 2006). Inborn errors of the IL-6 pathway and acquired autoantibodies against IL-6 have been detected in patients with HIES and staphylococcal infections, respectively (Puel and Casanova, 2019; Puel et al., 2008). The basis of the high serum IgE levels in P-Ger (P216Rfs*14/P216Rfs*14) remains unknown. WES was performed for P-Jap (C70Hfs*21/C70Hfs*21) and P-Ger (P216Rfs*14/P216Rfs*14) on genomic DNA extracted from their EBV-B cells. No candidate variants that could account for the HIES (P-Jap: C70Hfs*21/C70Hfs*21) or high serum IgE levels (P-Ger: P216Rfs*14/P216Rfs*14) of these patients were identified (data not shown). The responses to several other cytokines from the IL-10 superfamily, including IL-22, IL-26, IL-19, IL-20, and the three IFN-λs (also known as IL-28A, IL-28B, and IL-29), are thought to be TYK2 dependent (Commins et al., 2008; Ouyang and O’Garra, 2019; Schnepf et al., 2021). We therefore transiently transfected TYK2-deficient HEK293T or TYK2-deficient U1 fibrosarcoma cells with genes encoding each pair of receptors (IL-10R2 and IL-22R1 for IL-22; IL-10R2 and IL-20R1 for IL-26; IL-10R2 and IL-28R1 for IFN- λ; IL-20R2 and IL-20R1 for IL-19 and IL-20; and IL-20R2 and IL-22R1 for IL-20). These cells were then transfected with *TYK2,* and we determined the proportions of phospho-STAT3-positive and phospho-STAT1-positive cells by flow cytometry. In these experimental conditions, a response to IFN-λ1 (IL-29) was observed with EV, and the response was only slightly enhanced by adding WT *TYK2* or a catalytically inactive *TYK* variant (P1104A, G634E, G996R, and A928V); no enhancement of the response was observed with R864C, G1010, or K930R (Fig. 5, C and D). Similar results were obtained for IL-26 stimulation, with only a modest increase in STAT3 phosphorylation relative to EV following transfection with WT *TYK2* (Fig. 5 E). These results confirm that the responses to IL-26 and IFN-λ are mainly TYK2 independent (Fuchs et al., 2016; Kreins et al., 2015; Schnepf et al., 2021). Finally, our results indicate that responses to IL-22, IL-19, and IL-20 are completely TYK2 independent, as shown by a comparison of the responses obtained following the transfection of the cells with an EV or with the WT *TYK2* allele (Fig. 5, F–I). Overall, our results indicate that, in the test conditions used, responses to IL-6 (other than in P-Jap), IL-22, IL-19, IL-20, and, to a lesser extent, IL-26 and IFN-λ, are independent of TYK2.

### Immunophenotypes of primary leukocytes from TYK2-deficient patients

**Figure S4.**
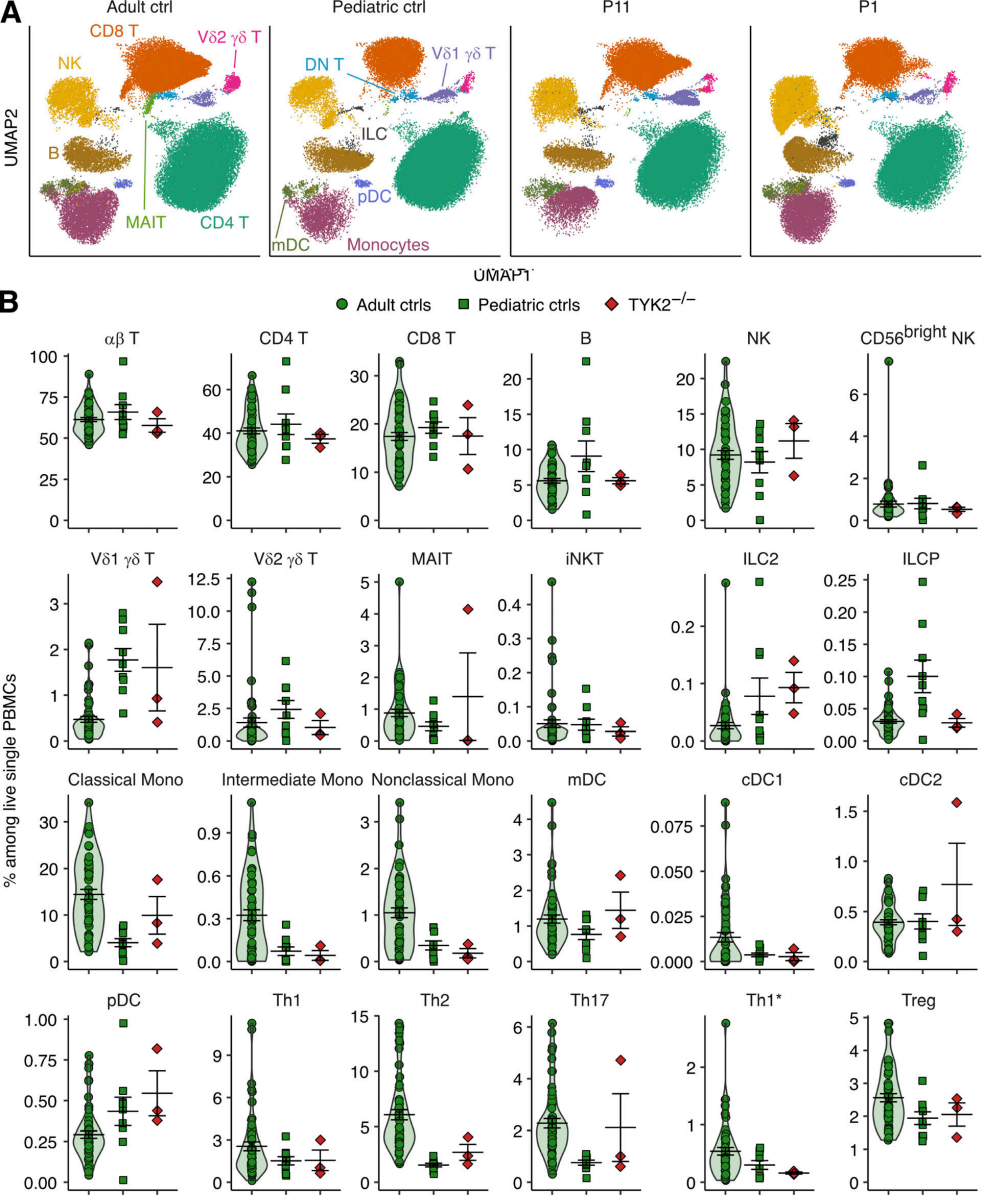
**Deep immunophenotyping of patients with complete TYK2 deficiency. (A)** UMAP representation of an adult control, a pediatric control, and two TYK2-deficient patients. The various cell subsets visualized are indicated. **(B)** Identification of the different cell subsets according to their abundance, measured as a percentage of live single PBMCs.

**Figure 6.**
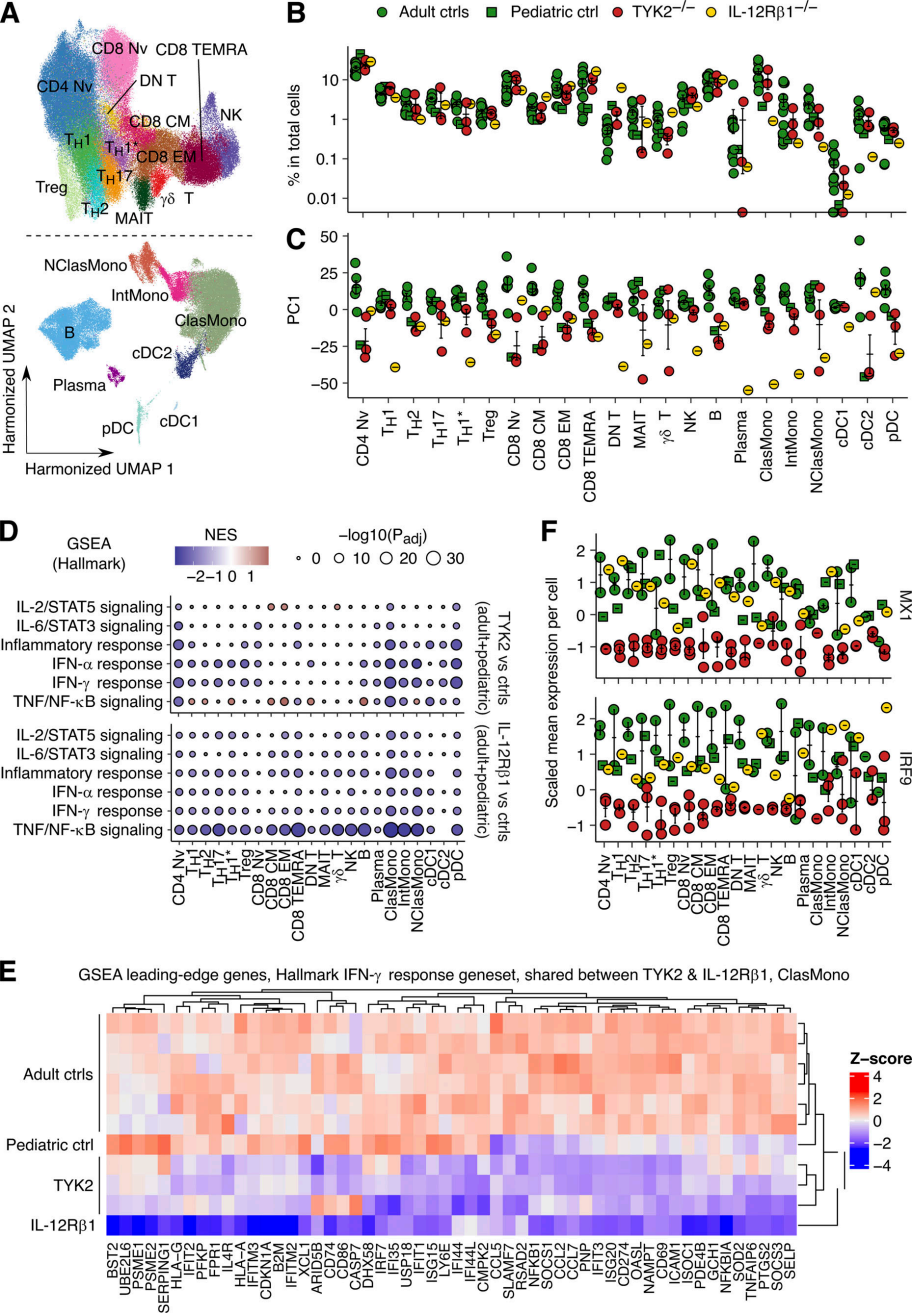
**scRNA-seq analysis of patients with complete TYK2 deficiency. (A)** UMAP representation of the different subsets of myeloid and lymphoid leukocyte subsets. PBMCs from seven controls (including one pediatric control), one IL-12Rβ1-deficient patient, and three TYK2-deficient patients (P-Tur [L767*/L767*], P1 [C70Hfs*21/C70Hfs*21], and P11 [P216Rfs*14/P216Rfs*14]) were analyzed. **(B)** Proportions of leukocyte subsets. **(C)** Pseudobulk principal component analysis. **(D)** GSEA. TYK2- and IL-12Rβ1–deficient cells were compared with healthy controls. Immune-related gene sets were chosen for visualization. **(E)** Heatmap analysis of the GSEA leading-edge genes for the Hallmark IFN-γ response gene set shared between TYK2- and IL-12Rβ1-deficient classic monocytes. Normalized *Z*-transformed pseudobulk read counts are shown. **(F)** Single-cell expression levels of *MX1* and *IRF9* mRNA. Only cells from healthy controls studied in the same batch of experiment with TYK2- and IL-12Rβ1-deficient patients are analyzed. CM, central memory; EM, effector memory; DN, double negative; MAIT, mucosal associated invariant T cells; TEMRA, terminally differentiated effector memory T cells; ClasMono, classical monocytes; IntMono, intermediate monocytes; NClasMono, nonclassical monocytes; cDC, conventional dendritic cells; pDC, plasmacytoid dendritic cells.

We studied the cellular basis of mycobacterial and viral diseases in TYK2-deficient patients by studying peripheral blood mononuclear cells (PBMCs) from P-Tur (L767*/L767*), P1 (C70Hfs*21/C70Hfs*21), and P11 (P216Rfs*14/P216Rfs*14). These three patients are homozygous for three different LOF alleles and have complete TYK2 deficiency. We compared these cells with PBMCs from both healthy individuals and patients with complete IL-12Rβ1 deficiency. Flow cytometric immunophenotyping showed normal numbers of blood cells for both the myeloid and lymphoid leukocyte subsets, including purely adaptive T cells (CD4 T, CD8 T, and their subsets), innate-like adaptive T cells (γδ T, mucosal associated invariant T, and invariant natural killer [NK] T), innate lymphoid cells (NK, innate lymphoid cell progenitors [ILCP], and ILC2), monocytes, and dendritic cells, in the three TYK2-deficient patients (Fig. S4). We then searched for more subtle alterations by performing single-cell RNA-seq (scRNA-seq) on PBMCs from the three TYK2-deficient patients and one IL-12Rβ1–deficient patient (Fig. 6). Clustering analysis revealed normal numbers of the 22 discrete leukocyte subsets detected in the three TYK2-deficient patients (Fig. 6, A and B). Pseudobulk principal component analysis (PCA) revealed that both classic and intermediate monocytes from the three TYK2-deficient patients had a transcriptional profile intermediate between those of healthy controls and an IL-12Rβ1–deficient patient (Fig. 6 C). TYK2-deficient and IL-12Rβ1–deficient classic monocytes commonly showed downregulation of genes involved in IFN-γ immunity (Fig. 6, D and E). On the other hand, multiple leukocyte subsets from the TYK2-deficient, but not IL-12Rβ1–deficient, patients showed reduced expression of *MX1* and *IRF9*, crucial components of the antiviral responses mediated by TYK2-dependent type I IFN signaling (Zhang, 2020a; Zhang S, 2020b; Fig. 6 F). Our analysis thus suggested that TYK2 deficiency impairs both antiviral type I IFN immunity and antimycobacterial type II IFN immunity in the basal state in vivo in monocytes and T cells, with no or minimal effect on the development of these lineages per se.

### Inherited TYK2 deficiency impairs responses to IL-23 and IFN-α across leukocytes

**Figure 7.**
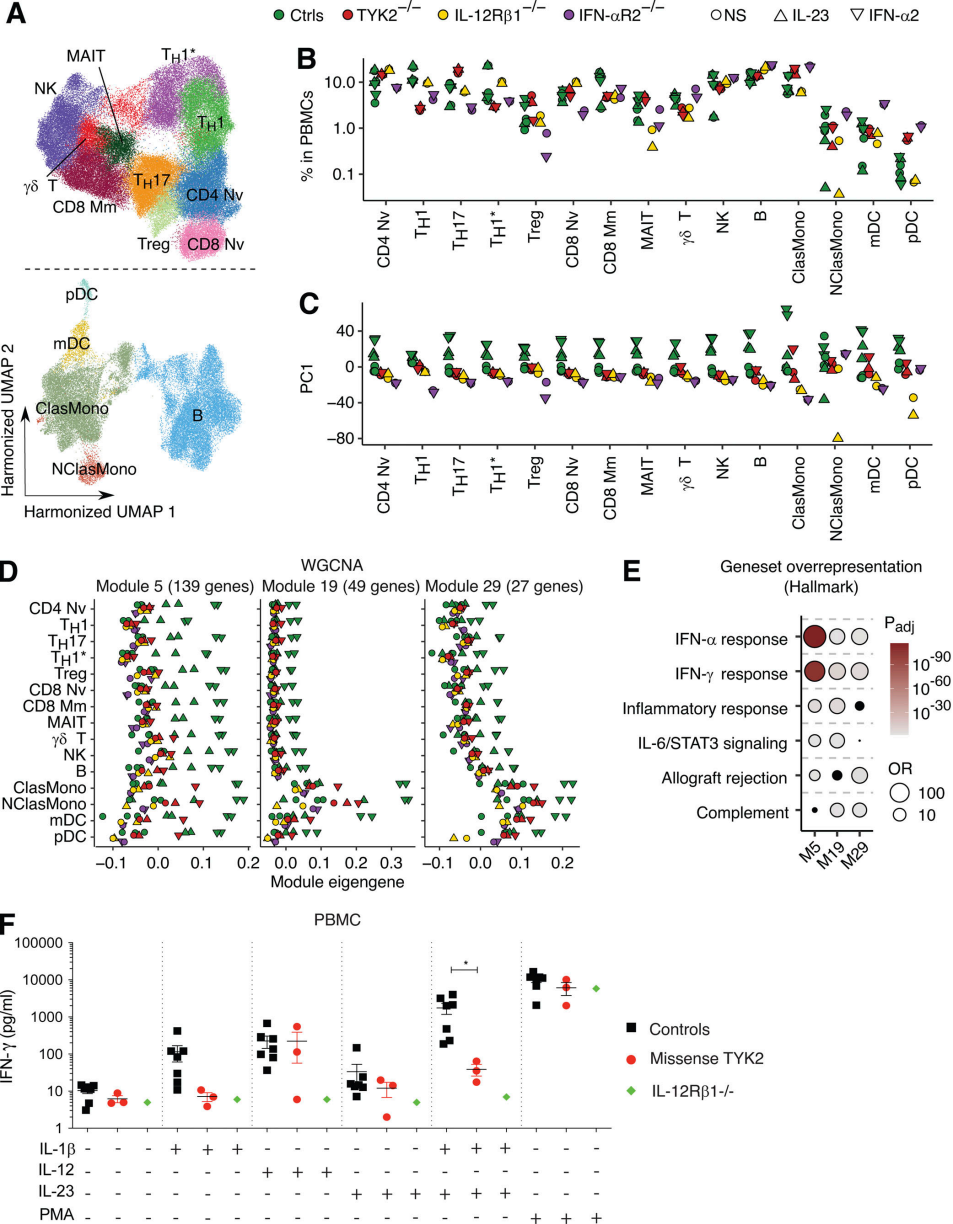
**Analysis of cellular responses to IL-23 and IFN-α2 in PBMCs. (A–E)** Cryopreserved PBMCs from two healthy controls, one TYK2-deficient patient (P-Tur [L767*/L767*]), one IL-12Rβ1–deficient patient, and one IFN-αR2–deficient patient were either left nonstimulated or were stimulated with IL-23 or IFN-α2 for 6 h, before being subjected to scRNA-seq analysis. Three batches of experiments were integrated via Harmony (Korsunsky et al., 2019). **(A)** Unsupervised clustering followed by manual identification with the aid of the SingleR pipeline (Aran et al., 2019) guided by the MonacoImmuneDataset (Monaco et al., 2019). **(B)** Relative abundance of each leukocyte subset according to clustering analysis. **(C)** Batch-corrected pseudobulk PCA. The first principal components are shown for each individual leukocyte subset. **(D)** Batch-corrected pseudobulk WGCNA. Three stimulation-dependent modules of coexpressed genes were identified. **(E)** Gene set overrepresentation analysis for genes in the three modules in D. Four gene sets with the highest odds ratio were selected for each module. Black dots are not statistically significant. **(F)** IFN-γ production by PBMCs from healthy controls and TYK2-deficient patients (two patients homozygous for the P1104A TYK2 variant and P5, homozygous for the G634E TYK2 variant), together with an IL-12Rβ1–deficient patient as a negative control, following stimulation with IL-12, IL-23, IL-1β, or a combination of IL-1β and IL-23. PMA-ionomycin was used as a control. *, P < 0.05, two-tailed Student’s *t* tests with Welch’s correction. Nonsignificant values are not indicated. MAIT, mucosal associated invariant T cells; ClasMono, classical monocytes; NClasMono, nonclassical monocytes; pDC, plasmacytoid dendritic cells.

**Figure S5.**
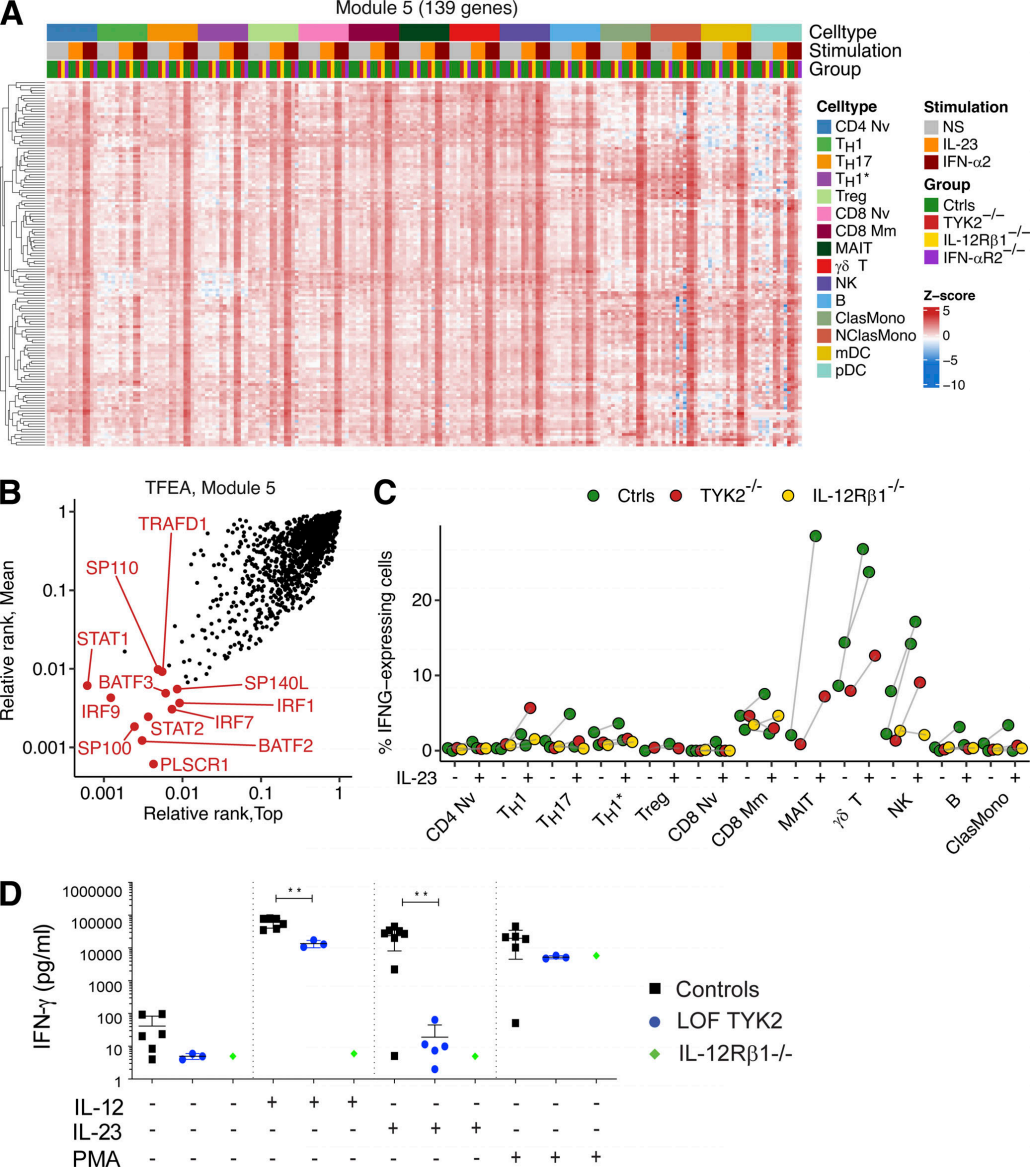
**Impaired IL-23–dependent IFN-γ induction in TYK2-deficient patients. (A)** Heatmap analysis of batch-corrected Z-transformed normalized pseudobulk read counts for genes in module 5 across all leukocyte subsets identified. **(B)** TFEA with ChEA3 (https://maayanlab.cloud/chea3/; Keenan et al., 2019) for module 5 genes. **(C)** Single-cell expression of *IFNG* mRNA across leukocyte subsets. Percentages of cells containing at least one read for *IFNG* are quantified. A given individual-cell type pair was excluded from the analysis if <100 cells were available. **(D)** IFN-γ production following stimulation by IL-1β, IL-12, and IL-23 or a combination in PBMC from controls, TYK2-deficient patients (P-Tur once and P17 twice), and an IL-12Rβ1–deficient patient, measured with a LegendPlex assay.

We further characterized the TYK2-dependent cellular responses to IL-23 and IFN-α, by performing scRNA-seq on PBMCs from one TYK2-deficient patient (P-Tur: L767*/L767*) either left nonstimulated or stimulated with IL-23 or IFN-α2 for 6 h. We simultaneously analyzed PBMCs from one IL-12Rβ1–deficient patient with and without IL-23 stimulation and PBMCs from one IFN-αR2–deficient patient with and without IFN-α2 stimulation as negative controls for the corresponding type of stimulation. Unsupervised clustering analysis identified 11 lymphoid and four myeloid leukocytic subsets (Fig. 7 A). Neither IL-23 nor IFN-α2 altered cell type abundances among the PBMCs of the TYK2-deficient patient (Fig. 7 B). However, pseudobulk PCA revealed a discernible shift of transcriptional profiles in the cells of healthy controls in response to IL-23 or IFN-α2, across all leukocyte subsets except Th1 and nonclassic monocytes (Fig. 7 C). By contrast, IL-12Rβ1–deficient and IFN-αR2–deficient cells displayed no transcriptional response to the corresponding stimuli (Fig. 7 C). Remarkably, the lymphoid cell subsets and pDCs of the TYK2-deficient patient displayed no transcriptional response to either IL-23 or IFN-α2, whereas TYK2-deficient classic monocytes and mDCs had impaired, but not abolished, transcriptional responses to IFN-α2 (Fig. 7 C). We further dissected the components of the IL-23– and IFN-α2–induced transcriptional modules, by applying the weighted gene coexpression network analysis (WGCNA) framework to the pseudobulk data. We identified three stimulation-dependent modules of coexpressed genes—modules 5, 19, and 29—consisting of 139, 49, and 27 genes, respectively (Fig. 7 D;Fig. S5 A). Module 5 contained genes induced by both IL-23 and IFN-α2 across all leukocyte subsets (e.g., *IFIT1/2/3/5*, *IRF7/9*, *ISG15/20*, and *MX1/2*), whereas modules 19 and 29 contained genes predominantly induced by IFN-α2 in classic/nonclassic monocytes and mDCs (e.g., *CD274*, *CXCL9/10/11*, *SIGLEC1*, and *JAK2*; Fig. 7, D and E). No module of genes induced by IL-23 but not by IFN-α2 was identified, suggesting that most genes induced by IL-23 overlap with type I ISGs. Transcription factor enrichment analysis (TFEA) predicted the involvement of STAT1/2/IRF1/7/9 in the regulation of module 5 genes (Fig. S5 B). Overall, human TYK2 governs cellular responses to both IL-23 and IFN-α2 across leukocyte subsets.

### Impaired IL-23–dependent IFN-γ production in TYK2-deficient patients

IFN-γ–mediated immunity is crucial for protection against mycobacterial diseases (Boisson-Dupuis and Bustamante, 2021). We therefore evaluated IFN-γ induction after IL-23 stimulation in PBMC from TYK2-deficient patients, comparing the results with those for healthy individuals and IL-12Rβ1–deficient patients. Focusing on *IFNG* in the scRNA-seq data, we evaluated the percentage of cells producing *IFNG* mRNA with and without IL-23 stimulation. Single-cell *IFNG* expression increased in control mucosal associated invariant T, γδ T, and NK cells, but is impaired in the TYK2-deficient patient’s cells (Fig. S5 C). We also showed that PBMCs from controls secreted IFN-γ in response to IL-12, IL-23, and a combination of IL-23 and IL-1β (known to potentiate IL-23; Serafini et al., 2022) as determined by multiplex ELISA (Fig. 7 F). By contrast, in patients with TYK2 missense variants (P1104A and G634E homozygotes), IFN-γ production was impaired upon IL-1β and IL-23 stimulation but preserved upon IL-12 stimulation (Fig. 7 F). Furthermore, patients with complete TYK2 deficiency displayed impaired IL-12– and IL-23–mediated IFN-γ production (Fig. S5 D), whereas IL-12– and IL-23–mediated IFN-γ production was completely abolished in the IL-12Rβ1–deficient patient. As a positive control, IFN-γ production upon stimulation with PMA plus ionomycin was similar for all controls and patients. Thus, all TYK2-deficient patients (complete TYK2 deficiency with or without TYK2 protein production, partial TYK2 deficiency across signaling pathways, or rare or common partial TYK2 deficiency specific for IL-23 signaling) have impaired IL-23–dependent IFN-γ production, probably largely accounting for their mycobacterial diseases.

## Discussion

We have characterized 19 new patients with AR TYK2 deficiency, including 13 with complete TYK2 deficiency and no TYK2 protein production. We also report the first patient with complete TYK2 deficiency with protein production (P9, homozygous for the G1010D allele). These patients have similar cellular phenotypes for the response to TYK2-dependent cytokines, with cellular responses to IFN-α, IL-10, IL-12, and IL-23 impaired, but not abolished, as already observed in the 15 previously reported patients (including P7, P12, P14, and P15 described in Sarrafzadeh et al. [2020]; Zhang et al. [2022]) with a complete lack of TYK2 protein (Fuchs et al., 2016; Kreins et al., 2015; Minegishi et al., 2006; Sarrafzadeh et al., 2020; Wu et al., 2020; Zhang et al., 2022). We also show that cellular responses to IL-10 family cytokines (IL-26, IL-22, IL-20, IL-19, and IFN-λs; Schnepf et al., 2021) are intact in the absence of TYK2. The G1010D mutation is LOF across signaling pathways because the resulting protein has no kinase activity and cannot serve as a substrate for phosphorylation, like the kinase-dead and substrate-dead K930R control mutant.

The 25 known patients with complete TYK2 deficiency (including 15 previously reported and 10 reported herein) have suffered from intramacrophagic infections (mostly due to mycobacteria) or viral infections (mostly due to herpesviruses) or both. These infections result from impaired IL-12/IL-23–mediated IFN-γ production by lymphocytes (Boisson-Dupuis and Bustamante, 2021; Bustamante, 2020) and impaired responses to IFN-α/β across cell types (Meyts and Casanova, 2021). We found that 48% of the 25 patients with AR complete TYK2 deficiency had mycobacterial diseases (due to BCG, EM, or *M.tb*) and 16% had salmonellosis, brucellosis, or leishmaniasis, as often reported in patients with MSMD (de Beaucoudrey et al., 2010; Fieschi et al., 2003; Parvaneh et al., 2017; van de Vosse et al., 2013). Moreover, 60% had viral diseases (due to HSV-1, VZV, influenza A virus, SARS-CoV-2, respiratory syncytial virus [RSV], EBV, CMV, and MMR), and 16% had fungal diseases (mainly due to *C. albicans,* with chronic mucocutaneous candidiasis in one case), which is more common in patients with IL-12Rβ1 or IL12p40 deficiencies, due to the impairment of IL-17 immunity (Puel et al., 2011; Kreins et al., 2015; Li et al., 2019).

Penetrance was incomplete for mycobacterial and viral diseases, probably due to the impairment, but not total abolition of responses to IL-12/IL-23 and IFN-α/β, respectively, and the variability of microbial exposure and infectious inoculum. Indeed, 25 and 32% of the patients had isolated mycobacterial and viral diseases, respectively, whereas the remaining ∼40% had a combination of mycobacterial and viral diseases. 22 of the 25 patients with complete TYK2 deficiency had been vaccinated with BCG. Only eight of these patients presented BCG disease, attesting to the incomplete clinical penetrance of TYK2 deficiency for MSMD. Incomplete penetrance was observed for viral diseases as well, which were rarely life-threatening. It is remarkable that even the most common viral pathogens in these patients, HSV-1 and VZV, rarely cause disease, with life-threatening disease rarer still. However, the occurrence of HSE in one child confirms the importance of type I IFN in cortical neuron intrinsic immunity to HSV-1 (Bastard et al., 2021). Mucocutaneous candidiasis is even less penetrant in these patients than in IL-12Rβ1–deficient patients (Kreins et al., 2015).

We also describe two other previously unknown forms of AR TYK2 deficiency in six patients. We characterized a partial form of TYK2 deficiency, with a deleterious impact evenly distributed across all TYK2-dependent pathways, in two patients (P3 and P4, homozygous for R864C). Cells homozygous for this variant have a response to IFN-α/β intermediate between the responses of cells homozygous for the WT allele and cells homozygous for a LOE *TYK2* allele. Consistently, this allele was also hypomorphic for IL-12 signaling in an overexpression system. The mechanism involved is similar to that for K930R, with effects on the capacity of the protein to phosphorylate other proteins (as an enzyme) and to be phosphorylated (as a substrate). These functions are completely abolished for the LOF variant K930R, but only reduced for the hypomorphic variant R864C. Nevertheless, this proband was clinically indistinguishable from the patients with complete TYK2 deficiency. By contrast, his sister, who was not vaccinated with BCG, remains asymptomatic at the age of 4 yr.

We also discovered and characterized three previously unknown rare missense variants underlying specific IL-23 signaling deficiency in three patients (G634E, G996R, and A928V). The mechanism underlying this deficiency is similar to that of P1104A, involving the abolition or severe impairment of catalytic activity, but with preservation of the capacity of the resulting protein to serve as a substrate for phosphorylation upon activation. Like patients homozygous for P1104A (Boisson-Dupuis et al., 2018), these new patients suffered from mycobacterial infection: one patient was infected with *M. fortuitum* and two with *M.tb*. The proportions of patients with MSMD and TB were, therefore, similar to those previously documented for P1104A homozygosity in the same cohorts (Boisson-Dupuis et al., 2018; Kerner et al., 2021; Kerner et al., 2019). In addition, one patient had nocardiosis and infections caused by Enterobacteriaceae, as also reported in patients with IL-12Rβ1 or IL-12p40 deficiencies (Bustamante et al., 2014; Fieschi et al., 2003).

**Figure 8.**
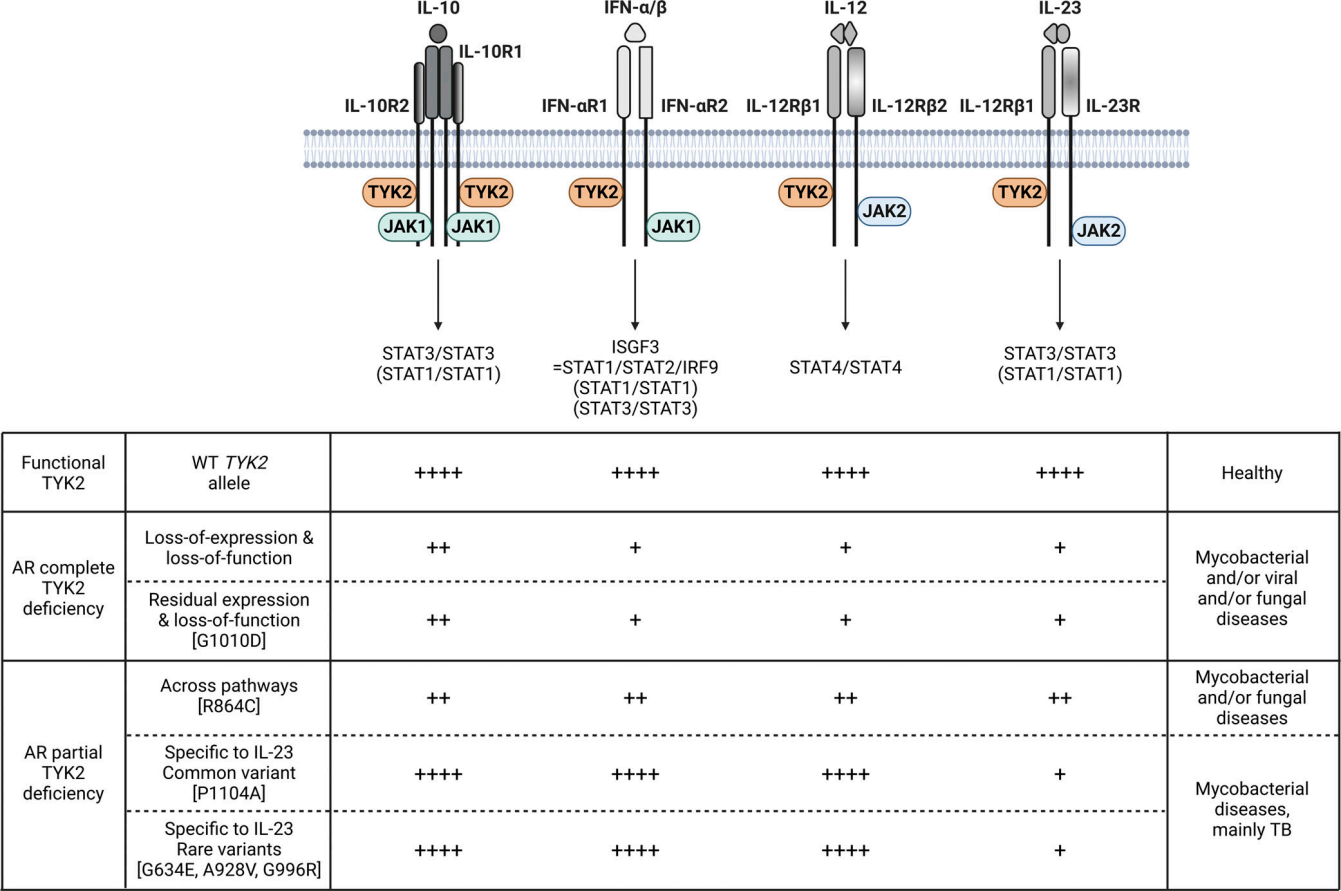
Schematic representation of the TYK2-dependent signaling pathways and the resulting functional deficiency in the five forms of TYK2 deficiency identified.

We define three previously unknown forms of TYK2 deficiency in total, expanding the family of TYK2 deficiencies to five distinct forms (Table 1 and Fig. 8). Remarkably, one of the new forms selectively impairs cellular responses to IL-23 and underlies TB or MSMD in homozygotes, confirming our previous findings implicating the IL-23 pathway in the pathogenesis of mycobacterial disease in patients with IL-23R deficiency or homozygosity for *TYK2* P1104A (Fieschi and Casanova, 2003; Boisson-Dupuis et al., 2018; Martinez-Barricarte et al., 2018; Kerner et al., 2019; Boisson-Dupuis and Bustamante, 2021; Casanova and Abel, 2022). Impairment of the IL-23–dependent induction of IFN-γ appears to be the only mechanism of mycobacterial disease common to patients with the five forms of AR TYK2 deficiency. Additional impairment of the IL-12-dependent induction of IFN-γ  in three of the five forms of TYK2 deficiency is apparently associated with more severe mycobacterial disease. The other two forms suggest that IL-12 cannot compensate for defective human IL-23 to ensure efficient IFN-γ immunity to intramacrophagic pathogens.

## Materials and methods

### Human participants

Healthy volunteers were recruited at The Rockefeller University. The patients and their family members were recruited at Necker Hospital for Sick Children. Written informed consent was obtained from all patients and healthy volunteers enrolled in this study. For minors, parental consent was obtained for the study of biological materials and for the reporting of the findings. The study was approved by the institutional ethics committees of The Rockefeller University and Necker Hospital for Sick Children and was performed in accordance with the requirements of these bodies, in accordance with local regulations, and with the approval of the institutional review boards of the corresponding institutions.

### Case reports

#### Patient 1 (kindred A, II.2)

A 17-mo-old girl (Fig. 1 B) from Iran suffered from bronchiolitis starting at the age of 4 mo. The causal microbes were identified as RSV and adenovirus by PCR. This patient also had CMV viremia, which was treated with gancyclovir. She was immunized with BCG vaccine at birth and did not develop an adverse reaction. Her lymphocyte and Ig levels were within the normal range. She has never developed dermatitis or fungal, intracellular, or staphylococcal infections. She had normal levels of Ig, including IgE. She developed an intracranial hemorrhage and underwent surgery in Thailand for hydrocephalus. Her CMV titers on PCR subsequently increased, and she was kept on gancyclovir for prophylaxis. The patient’s serum IgE levels were determined three times, and the highest value obtained was in the normal range (76 kU/liter). The patient had no atopy, dermatitis, or fungal infections.

#### Patient 2 (kindred B, II.2)

The patient (Fig. 1 B) was a 5-yr-old girl from Iran. At 6 mo, she developed BCG-itis, following BCG vaccination. She also had generalized HSV infection and allergic dermatitis. 2 mo later, she suffered an episode of oral candidiasis. At the age of 1 yr, the patient presented disseminated BCG-osis (submandibular, cervical, and axillary). She was treated with anti-TB drugs, which induced hepatitis. She developed cutaneous leishmaniasis, with *Staphylococcus aureus* superinfection.

#### Patients 3 and 4 (kindred C, II.1 and II.2)

P3 was a 5-yr-old boy (Fig. 1 B) from Iran suffering from bilateral axillary and cervical lymphadenopathy 8 mo after BCG vaccination. His parents were consanguineous. He had been hospitalized on several occasions for HSV infections by the age of 3 yr. He also suffered from fungal infections, including mild oral candidiasis, which ceased on antimycobacterial therapy. The levels of serum Ig, lymphocytes, and nitroblue tetrazolium were within the normal range in routine laboratory tests. His younger sister, P4, an 11-mo-old girl not immunized with BCG, was clinically healthy.

#### Patient 5 (kindred D, II.1)

Patient 5 (Fig. 1 B) was a 31-yr-old woman of Asian descent who had suffered recurrent episodes of infection since her early teens. These infections included *M. fortuitum* chronic osteomyelitis, *Nocardia* infection, and recurrent septicemia caused by Enterobacteriaceae (including *Klebsiella pneumoniae*, *Escherichia coli*, *Proteus mirabilis*, and *Enterobacter spp.*). She also suffered *Candida* urosepsis and *Clostridium difficile* infection. She tested negative for HIV, and her lymphocyte subsets and Ig levels were found to be predominantly in the normal range. This patient also had type 1 diabetes mellitus and hypothyroidism. She still requires repeated courses of antibiotics for her infections and is regularly treated with Ig.

#### Patient 6 (kindred E, II.1)

Patient 6 (Fig. 1 B) was a Moroccan woman born in 1988. She presented smear-positive pulmonary tuberculosis at the age of 24 yr. She was treated with streptomycin, isoniazid, rifampicin, and pyrazinamide for 2 mo, and then with rifampicin and isoniazid for 4 mo, and she recovered. She was vaccinated at birth with BCG, without adverse effects.

#### Patient 7 (kindred F, II.2)

The clinical report for this patient (Fig. 1 B) has been published (Sarrafzadeh et al., 2020). Briefly, this patient was an Iranian boy born to consanguineous parents. Following BCG vaccination at birth, he was diagnosed with BCG-osis at the age of 7 mo. All Ig levels, including IgE levels, were in the normal range. P7 did not have atopic dermatitis or staphylococcal disease. He suffered two episodes of HSV, at the ages of 3 and 7 yr, presenting as fever and gingivostomatitis, respectively; one episode of VZV infection at the age of 6.5 yr; and aseptic meningitis at the age of 6 yr.

#### Patient 8 (kindred G, II.3)

This patient (Fig. 1 B) was an 8-yr-old boy from Saudi Arabia who was born at full term, after an uneventful pregnancy, to consanguineous parents. The birth was a normal spontaneous vaginal delivery. P8 developed normally until the age of 2 yr and 8 mo, when he experienced a fever that lasted 1 mo, followed by generalized lymphadenopathy. Investigations at a local hospital were unremarkable, and he was treated with a prolonged course of antibiotics. At the age of 4 yr, he was admitted to the hospital with severe gastroenteritis, complicated by hypovolemic shock, requiring pediatric intensive care unit (PICU) admission for 1 mo. He then developed brain damage, with neurodevelopmental regression and a seizure disorder. The family mentioned a history of recurrent chest infections beginning at the age of 4 yr. The chest infections improved with nebulization and antibiotics, requiring hospital admission almost every 2 mo. The patient had one episode of HSV gingivostomatitis. The patient’s family took him to a hospital in Jordan for a second opinion. While there, he received a 3-mo course of steroid treatment for presumed acute disseminated encephalomyelitis (the MRI report was not available). The family noted an improvement in the patient’s level of consciousness and his sleeping pattern, and the patient became less irritable, with better control of his seizures. Some examinations were performed at the local hospital. Basic immunological tests, metabolic tests (including determinations of very long-chain fatty acids, ammonia, lactic acid, serum amino acids, creatine kinase, and urine organic acids), sweat chloride tests, anti-nuclear antibody screening, lysosomal enzyme studies for storage disorder, and neurodegenerative screening were performed; all the results were normal. *C. albicans* was detected once in tracheal aspirate during an episode of chest infection. The patient was vaccinated with MMR without adverse effects.

#### Patient 9 (kindred H, II.2)

This patient (Fig. 1 B) was a girl born in 2013 to first-degree consanguineous Turkish parents. She was treated for meningitis at the age of 3 mo and for pneumonia requiring hospitalization as an inpatient twice in 1 yr (at 3 and 3.5 yr of age). On one of these occasions, PCR tests for respiratory viruses were performed on nasal swab samples: the results were positive for RSV A/B and coronavirus 229. This patient had recurrent upper respiratory tract infections. Her physical examination was normal, as were the other laboratory test results.

#### Patient 10 (kindred I, II.1)

This patient (Fig. 1 B) was a girl from Chile born in 2000. She suffered from miliary tuberculosis at the age of 10 yr. She was treated and survived but with heavy neurological sequelae: tetraplegia and epilepsy.

#### Patients 11 and 12 (kindred J, II.2 and II.3)

Patient P11 (Fig. 1 B) was a Turkish girl born in 2019 to second-degree consanguineous parents. She was vaccinated with BCG at birth, with no adverse reaction. She was admitted to hospital for sepsis at the age of 6 d, and was treated for 26 d. At the age of 4 mo, she was intubated for 14 d in intensive care for influenza A pneumonia. She has been hospitalized many times for recurrent viral pneumonia (rhinovirus, RSV), and noninvasive ventilation in the PICU was required once. She was vaccinated against varicella and, 2 wk later, at the age of 15 mo, she had disseminated varicella infection. P12 (Fig. 1 B) is an older brother of P11, born in 2016. He was vaccinated with BCG, without complications. He was diagnosed with sepsis during the first month of life and was treated in an ICU for 20 d. At the age of 3 yr, he was hospitalized in intensive care for another 20 d for severe pneumonia due to influenza A. At the age of 4 yr, he was hospitalized for 15 d for severe pneumonia, suspected to be COVID-19 pneumonia, but with a negative test for SARS-CoV-2. No history of atopic dermatitis, high IgE levels, or staphylococcal infections was documented in either of these patients. Both children received the live vaccines against polio and MMR without adverse effects. They are both now receiving Ig replacement treatment and antimycobacterial and antiviral prophylaxis.

#### Patient 13 (kindred K, II.1)

This patient (Fig. 1 B) was a boy born in 2020 to consanguineous parents in Iran. He was vaccinated with BCG at birth and developed disseminated BCG disease at the age of 13 mo, which was treated with a combination of isoniazid, rifampin, ethambutol*,* levofloxacin, and amikacin. He has suffered from oral candidiasis since infancy, but has no signs of HIES (staphyloccocal infections, high IgE levels or atopic dermatitis). His family is related to a previously described family with the same mutation (P5 and P6, described by Kreins et al. [2015]).

#### Patient 14 (kindred L, II.1)

This patient (Fig. 1 B) was a Turkish girl born in 2018 to second-degree consanguineous parents. She was vaccinated with BCG at birth without complications. She was hospitalized for 10 d, for neonatal sepsis, in the first month of life. She was then hospitalized twice in the ICU: at 2 mo of age for fever and dyspnea (3 d in the ICU), and then at 3 mo of age for fever, diarrhea, and dyspnea (14 d in the ICU). She was hospitalized at 5 mo of age for diarrhea and vomiting, and at 8 mo of age for hand-foot-and-mouth disease. At the age of 11 mo, she was diagnosed with Kawasaki disease (fever and rash). At the age of 13 mo, she was hospitalized for aphthous stomatitis. At the age of 1.5 yr, she was hospitalized for 10 d for acute COVID-19 pneumonia (PCR-positive); she did not require oxygen therapy. She was treated with azithromycin and hydroxychloroquine. At the age of 2 yr, following MMR vaccination (delayed due to the i.v. Ig used to treat Kawasaki fever), she developed fever, malaise, rash, and hepatomegaly (requiring hospitalization). She was diagnosed with rubella/measles (confirmed by the detection of IgG against measles). A second child in this family died at the age of 5 mo without a definitive diagnosis, after suffering from dyspnea, icterus, and hypotonia since the age of 2 mo. No genetic analysis was possible for this child.

#### Patient 15 (kindred M, II.2)

This patient (Fig. 1 B) was a Turkish girl born in 2006. At the age of 15 yr, she was admitted to a pediatric surgical department for severe abdominal pain. Purulent fluid was detected during abdominal surgery, and piperacillin/tazobactam and teicoplanin treatment was initiated. Generalized white granulomatous lesions were detected in her peritoneal material, and tuberculosis was suspected. Acid-fast bacillus tests were negative on stomach fluid but strongly positive on peritoneal material. In addition, a sputum PCR test was positive for *M.tb*, and *M.tb* grew in her sputum culture. This patient is now receiving quadritherapy with isoniazid (1× 300 mg), rifampicin (1× 600 mg), pyrazinamide (1× 1.5 g), and ethambutol (1× 1 g). This patient tested positive for anti–COVID-19 antibodies but did not develop any symptoms.

#### Patient 16 (kindred N, II.1)

This patient (Fig. 1 B) was a Turkish boy born in 2011 to nonconsanguineous parents. At the age of 9 yr, he was hospitalized for 2 wk in an inpatient clinic for lobar pneumonia. He was then admitted to the ICU for high-flow oxygen therapy for 4 d. He was given levofloxacin, teicoplanin, clindamycin, and methylprednisolone during this hospitalization. He was vaccinated with BCG at birth, with no adverse effects. He attended irregular follow-up visits at the pediatric allergy department for asthma. He was hospitalized several times for bronchiolitis and received inhaler therapy. His immunological evaluations, including IgE determinations in particular, were all normal. This patient does not have an HIES phenotype, with no eczema or atopic dermatitis, skeletal involvement (bone fracture, coarse face), or skin viral infections or high serum IgE levels. He suffered anaphylaxis on amoxicillin treatment.

#### Patients 17 and 18 (kindred O, II.2 and II.1)

These two patients (Fig. 1 B) originated from a consanguineous Pakistani family. P17 was hospitalized for COVID-19 and required 2 d of treatment with i.v. fluids to treat a fever. She was not vaccinated with BCG. Her IgE levels were normal. Her older sister, P18, was born in 2014 and remains asymptomatic. She has not been vaccinated against BCG. She had dry skin and high IgE levels (682 IU/ml).

#### Patient 19 (kindred P, II.1)

This patient (Fig. 1 B) was a 7-yr-old girl originating from Turkey. At 8 mo of age, she suffered from CMV colitis (with colon perforation) and HSV sepsis with stomatitis aphthosis. At the age of 9 mo, her blood continued to test positive for HSV, and her urine for CMV. She received antiviral treatment (valgancyclovir) and antibacterial and antifungal prophylaxis and later presented recurrent oral HSV infection. At 17 mo of age, she suffered recurrent vomiting for 2 mo, her urine tested positive for CMV (but not her blood or stools), the oral and perioral area tested positive for HSV-1, and *C. difficile* was detected in her stools. P19 suffered from pneumonia at the age of 2 yr, without identification of the causal microbe. At the age of 3 yr, she had gastroenteritis due to norovirus and, 1 mo later, bronchopneumonia with respiratory insufficiency due to EBV infection. At the age of 6 yr, she suffered from COVID-19 pneumonia, requiring 5 d of oxygen therapy, after which she presented gastroenteritis with positive tests for *Campylobacter* and noroviruses. Prophylaxis with acyclovir was prescribed, with a doubling of the dose for several days if any breakthrough infections occurred. The patient recovered, but with developmental delays.

### Plasmids, cell lines, and retroviral transduction of B cell lines

T cell lines (Herpesvirus Saimiri: HVS-T) and EBV-transformed lymphoblastoid cell lines (EBV-B cells) were generated by infecting PBMCs from healthy controls or patients with HVS or EBV, as previously described (Boisson-Dupuis et al., 2018). A retroviral pLZRS-IRES-dNGFR vector containing a puromycin resistance cassette was used, as previously described, for the stable transduction of EBV-B cells (Boisson-Dupuis et al., 2018). The mutant *TYK2* alleles used here were generated by site-directed mutagenesis, with specific primers and the PfuUltra II Hotstart PCR Master Mix (Agilent Technologies), first on the pMSCV-TYK2 backbone, according to the manufacturer’s instructions.

### Cell culture and stimulation

HEK293T cells were cultured in DMEM (Gibco) supplemented with 10% FBS (Invitrogen). EBV-B cells were cultured in RPMI (Gibco) supplemented with 10% FBS. HVS-T cells were cultured in a 1:1 mixture (by volume) of RPMI and Panserin 401 (PAN Biotech) supplemented with 10% FBS, GlutaMAX (350 µg/ml; Gibco), gentamicin (0.1 mg/ml; Gibco), and rhIL-2 (20 IU/ml; Roche).

The cells were starved for 2 h by incubation in serum-free RPMI. The cells were then left unstimulated or were stimulated with rhIFN-α 2b (10^5^ IU/ml; Schering), rhIL-23 (100 ng/ml; R&D Systems), rhIL-12 (20 ng/ml; R&D Systems), or rhIL-10 (50 ng/ml; PeproTech) for 5 min to assess the phosphorylation of JAKs, and for 30 min to assess the phosphorylation of STATs. For quantitative RT-PCR, cells were stimulated for 6 h with IL-10 (50 ng/ml) or IFN-α (10^5^ IU/ml). For RNA-seq experiments, cells were starved for 1.5 h in RPMI containing 1% FCS and were stimulated for 2 h with IFN-α (10^5^ IU/ml) and IL-21 (100 ng/ml). RNA was extracted with the Zymoresearch kit. For scRNA-seq experiments, PBMCs were either left unstimulated or were stimulated with 100 ng/ml of IL-23 or 10^3^ IU/ml IFN-α2b for 6 h.

### Generation of TYK2-deficient HEK293T cells

Two guide RNAs (gRNAs) were synthesized by Synthego, one binding to exon 6 and the other to exon 7. They were combined with recombinant cas9 protein to form a complex used for the nucleofection of HEK293T cells, in accordance with the manufacturer’s instructions. Cells were cloned by serial dilution, and PCR was used to select for deletions between the two gRNAs.

### Western blotting

Total protein was extracted from EBV-B or HVS-T cells in a lysis buffer containing 1% NP-40, 20 mM Tris-HCl, pH 7.4, 140 mM NaCl, 2 mM EDTA, and 50 nM NaF supplemented with 100 mM orthovanadate, 200 mM phenylmethylsulfonyl fluoride, 1% aprotinin, pepstatin (1 mg/ml), leupeptin (1 mg/ml), and antipain (1 mg/ml). Protein extracts were separated by SDS-PAGE, and the resulting bands were electroblotted onto polyvinylidene difluoride membranes. The blots were incubated for 1 h with a blocking solution consisting of Tris-buffered saline (TBS), 0.01% Tween 20 (Sigma-Aldrich), and 5% nonfat milk powder (Bio-Rad). The following primary antibodies were diluted 1:1,000 in blocking solution and incubated overnight with the blots: rabbit anti-phospho-Y1054/1055 TYK2 (Cell Signaling Technology), rabbit anti-phospho-1022/1023 JAK1 (Cell Signaling Technology), rabbit anti-phospho-1007/1008 JAK2 (Cell Signaling Technology), mouse anti-phospho-Y701 STAT1 (BD), rabbit anti-phospho-Y705 STAT3 (Cell Signaling Technology), rabbit anti-phospho-693 STAT4 (Cell Signaling Technology), mouse anti-STAT1 (BD), mouse anti-STAT3 (Cell Signaling Technology), rabbit anti-STAT4 (Cell Signaling Technology), mouse anti-tubulin (Santa Cruz Biotechnology), and rabbit anti-GAPDH (Santa Cruz Biotechnology) antibodies. The blots were washed three times, for 10 min per wash, in a washing buffer consisting of TBS plus 0.01% Tween 20. An anti-rabbit HRP or anti-mouse HRP-conjugated antibody (GE Healthcare) was then added at a dilution of 1:10,000 or 1:5,000, respectively, and the blots were incubated for 1 h. The blots were washed with washing buffer, and antibody binding was detected with the SuperSignal West Femto System (Thermo Fisher Scientific). The membranes were analyzed with an Amersham Imager 600 instrument (GE Healthcare Life Sciences).

### Flow cytometry analysis and labeling

Experiments were performed as previously described (Boisson-Dupuis et al., 2018). Briefly, for extracellular labeling, 1 million EBV-B cells were resuspended in PBS with Live Dead Aqua stain and incubated for 20 min at 4°C. Cells were washed in PBS-2% FCS and stained by incubation for 2 h with the specific or isotypic control antibodies: anti–IL10RB-488 (FAB874G; R&D) or isotypic control IgG1-isotype AF488 (IC002G; R&D), anti-IL12RB1 (556064; BD Biosciences) followed by donkey anti-mouse AF488 (A11001; BD Pharmingen), IFNAR1 (a gift from Sandra Pellegrini, France) followed by donkey anti-mouse AF488, and their respective isotypic controls (clone MOPC-21; 400102; BD Pharmingen). For the detection of intracellular STAT1 and STAT3 phosphorylation, HEK293T cells were stimulated as indicated, washed once with cold 1× PBS, and detached by pipetting. Cells were washed once in PBS-2% FCS, and fixed by incubation in fixation buffer (557870; BD Biosciences) for 10 min at 37°C. Cells were then washed with PBS-2%FCS and permeabilized by incubation with Perm Buffer III for 20 min at room temperature (558050; BD Bioscience). Cells were washed and stained by overnight incubation with AF647-anti-pSTAT3 and PE-anti-pSTAT1 antibodies (557815 and 562069; BD Biosciences) at 4°C. Cells were then washed, and events were acquired on a flow cytometer.

### WES, Sanger sequencing, and RNA-seq analyses

WES and Sanger sequencing were performed as previously described (Boisson-Dupuis et al., 2018; Kreins et al., 2015). For RNA-seq, reads were mapped with STAR v2.5.3a. A genome index specific to our data was first created, and single-end reads were then aligned in a two-pass mode in which novel splicing junctions were first detected, before final mapping. Read counts were obtained for each gene with HTSeq v0.9.1 (Anders et al., 2015). Homozygosity rates were estimated from exome data as previously described (Belkadi et al., 2016). Gene expression levels were estimated in TPM (transcripts per million). TPM estimates of expression levels take into account the size of each sample library and gene lengths (Conesa et al., 2016). Statistical analysis was performed with R v3.2.3 (Team, 2015; www.R-project.org/). Gene expression profiles are expressed as the fold-change in expression between the values obtained before and after stimulation. Raw data are available in SRA BioProject PRJNA856671.

### Deep immunophenotyping

Freshly thawed PBMCs (1.0∼1.5 × 10^6^ cells) were simultaneously stained with LIVE/DEAD Fixable Blue (Cat: L23105; 1:800 in PBS; Invitrogen) and blocked by incubation with FcR Blocking Reagent (1:25; Miltenyi Biotec) on ice for 15 min. The cells were washed and surface-stained with the following reagents on ice for 30 min: Brilliant Stain Buffer Plus (Cat: 566385, 1:5; BD Biosciences), anti-γδTCR-BUV661 (Cat: 750019, Clone: 11F2, 1:50; BD Biosciences), anti-CXCR3-BV750 (Cat: 746895, Clone: 1C6, 1:20; BD Biosciences), and anti-CCR4-BUV615 (Cat: 613000, Clone: 1G1, 1:20; BD Biosciences) antibodies. Cells were then washed and surface-stained with the following reagents on ice for 30 min: anti-CD141-BB515 (Cat: 565084, Clone: 1A4, 1:40; BD Biosciences), anti-CD57-FITC (Cat: 347393, Clone: HNK-1, 3:250; BD Biosciences), anti-Vδ2-PerCP (Cat: 331410, Clone: B6, 3:500; BioLegend), anti-Vα7.2-PerCP-Cy5.5 (Cat: 351710, Clone: 3C10, 1:40; BioLegend), anti-Vδ1-PerCP-Vio700 (Cat: 130-120-441, Clone: REA173, 1:100; Miltenyi Biotec), anti-CD14-Spark Blue 550 (Cat: 367148, Clone: 63D3, 1:40; BioLegend), anti-CD1c-Alexa Fluor 647 (Cat: 331510, Clone: L161, 1:50; BioLegend), anti-CD38-APC-Fire 810 (Cat: 356644, Clone: HB-7, 3:100; BioLegend), anti-CD27-APC H7 (Cat: 560222, Clone: M-T271, 1:50; BD Biosciences), anti-CD127-APC-R700 (Cat: 565185, Clone: HIL-7R-M21, 1:50; BD Biosciences), anti-CD19 Spark NIR 685 (Cat: 302270, Clone: HIB19, 3:250; BioLegend), anti-CD45RA-BUV395 (Cat: 740315, Clone: 5H9, 3:250; BD Biosciences), anti-CD16-BUV496 (Cat: 612944, Clone: 3G8, 3:500; BD Biosciences), anti-CD11b-BUV563 (Cat: 741357, Clone: ICRF44, 1:100; BD Biosciences), anti-CD56-BUV737 (Cat: 612767, Clone: NCAM16.2, 3:250; BD Biosciences), anti-CD4-cFluor 568 (Clone: SK3, 3:250; Cytek), anti-CD8-BUV805 (Cat: 612889, Clone: SK1, 3:250; BD Biosciences), MR1 tetramer-BV421 (1:100; National Institutes of Health Tetramer Core Facility), anti-CD11c-BV480 (Cat: 566135, Clone: B-ly6, 1:40; BD Biosciences), anti-CD45-BV510 (Cat: 563204, Clone: HI30, 3:250; BD Biosciences), anti-CD33-BV570 (Cat: 303417, Clone: WM53, 3:250; BioLegend), anti-iNKT-BV605 (Cat: 743999, Clone: 6B11, 1:25; BD Biosciences), anti-CD161-BV650 (Cat: 563864, Clone: DX12, 1:25; BD Biosciences), anti-CCR6-BV711 (Cat: 353436, Clone: G034E3, 3:250; BioLegend), anti-CCR7-BV785 (Cat: 353230, Clone: G043H7, 1:40; BioLegend), anti-CD3-Pacific Blue (Cat: 344824, Clone: SK7, 3:250; BioLegend), anti-CD20-Pacific Orange (Cat: MHCD2030, Clone: HI47, 1:50; Invitrogen), anti-CD123-Super Bright 436 (Cat: 62-1239-42, Clone: 6H6, 1:40; Invitrogen), anti-Vβ11-PE (Cat: 130-123-561, Clone: REA559, 3:500; Miltenyi Biotec), anti-CD24-PE-Alexa Fluor 610 (Cat: MHCD2422, Clone: SN3, 1:25; Invitrogen), anti-CD25-PE-Alexa Fluor 700 (Cat: MHCD2524, Clone: 3G10, 1:25; Invitrogen), anti-CRTH2-biotin (Cat: 13-2949-82, Clone: BM16, 1:50; Invitrogen), anti-CD209-PE-Cy7 (Cat: 330114, Clone: 9E9A8, 1:25; BioLegend), anti-CD117-PE-Dazzle 594 (Cat: 313226, Clone: 104D2, 3:250; BioLegend), anti-HLA-DR-PE-Fire 810 (Clone: L243, 1:50; BioLegend), and anti-CD66b-APC (Cat: 1305118, Clone: G10F5 1:50; eBioscience) antibodies. After washing, cells were further incubated with streptavidin-PE-Cy5 (Cat: 405205, 1:3,000; BioLegend) on ice for 30 min. Cells were then washed, fixed with 1% paraformaldehyde/PBS, washed again, and acquired with an Aurora cytometer (Cytek). Subsets were manually gated with FlowJo, and results were visualized with R.

### scRNA-seq

For scRNA-seq analysis of primary leukocytes at baseline, cryopreserved PBMCs from six healthy controls (including previously generated data of four healthy controls [Spaan et al., 2022], one of whom was tested twice), three TYK2-deficient patients, and one IL-12Rβ1–deficient patient were analyzed. For scRNA-seq analysis following stimulation, cryopreserved PBMCs from two healthy controls, one TYK2-deficient patient, one IL-12Rβ1–deficient patient, and one IFN-αR2–deficient patient were analyzed.

Cells were filtered through a MACS SmartStrainer with 70-µm pores (Cat: 130-098-462; Miltenyi Biotec) to remove large debris, washed three times with PBS plus 0.5% FBS, and finally filtered through a Falcon Cell Strainer with 40-µm pores (Cat: 352340; Corning), before being subjected to single-cell capture via the 10X Genomics Chromium chip. Libraries were prepared with the Chromium Single Cell 3′ Reagent Kit (v3 Chemistry) and sequenced with an Illumina NovaSeq 6000 sequencer. Sequences were preprocessed with CellRanger. Approximately 10,000 cells were sequenced per sample. For the baseline scRNA-seq analysis, data generated during this study were analyzed together with the healthy control data generated in the previous study (Spaan et al., 2022), and publicly available control PBMC datasets downloaded from the 10X Genomics web portal (https://support.10xgenomics.com/single-cell-gene-expression/datasets). Data were filtered manually on the basis of common quality-control metrics and integrated with Harmony (Korsunsky et al., 2019). Two sequential graph-based clustering analyses were performed. The first-round clustering identified general leukocyte subsets, and the second-round clustering identified memory and effector T lymphocyte subsets and NK lymphocytes at sufficiently high resolution. Clusters were identified with the SingleR pipeline (Aran et al., 2019) guided by MonacoImmuneData, and cell type-specific marker gene expression was then assessed by manual inspection (Monaco et al., 2019). The CITE-Seq datasets obtained from the 10X Genomics web portal also provided information about the identity of each cluster. Clusters were visualized by Uniform Manifold Approximation and Projection (UMAP). After cluster identification, 10X datasets were excluded from subsequent analyses.

Gene expression was quantified at the single-cell level with Seurat (Hao et al., 2021). Pseudobulk analysis was performed by aggregating all reads from cells assigned to a given cluster, as previously described (Crowell et al., 2020). We performed PCA on the read counts normalized through variance-stabilizing transformation with batch correction, using the removeBatchEffect function implemented in limma (Ritchie et al., 2015). Differential expression analysis was performed with DESeq2 (Love et al., 2014). GSEA was conducted with the fgsea package, by projecting the fold-change ranking onto various MSigDB gene sets (http://www.gsea-msigdb.org/gsea/msigdb/genesets.jsp). WGCNA was performed in R (Langfelder and Horvath, 2008). TFEA was performed with ChEA3 (https://maayanlab.cloud/chea3/; Keenan et al., 2019). All analyses were performed in R v4 (http://www.R-project.org/). Raw data are available in SRA BioProject, accession no. PRJNA856671.

### Cytokine stimulation of PBMCs

PBMCs were dispensed into a U-bottom 96-well plate at a density of 2 × 10^5^ cells per well, in 100 μl of lymphocyte medium per well. Cells were incubated with or without recombinant human IL-12 (50 ng/ml; R&D Systems) or recombinant human IL-23 (100 ng/ml, 1290-IL; R&D Systems), with or without IL-1β (2.5 ng/ml). After 48 h of stimulation, supernatants were collected and stored at −20°C for determination of cytokine concentrations.

### Online supplemental materials

The supplementary information describes in greater depth the mutations and effects at the mRNA and protein level of P2 (Fig. S1) and P8 and P9 (Fig. S2). Additional analysis of RNA-seq (Fig. S3), deep immunophenotyping (Fig. S4), and scRNA-seq (Fig. S5) are also provided. Table S1 lists the differentially expressed genes between cytokine stimulated and non-stimulated samples in EBV-B cells from controls and patients.

## Supplementary Material

Table S1 lists the differentially expressed genes between cytokine stimulated and non-stimulated samples in EBV-B cells from controls and patients.

SourceData F2 contains original blots for Fig. 2.

SourceData F3 contains original blots for Fig. 3.

SourceData F5 contains original blots for Fig. 5.

SourceData FS2 contains original blots for Fig. S2.
