# Supplementary material for: Impaired IL-23–dependent induction of IFN-γ underlies mycobacterial disease in patients with inherited TYK2 deficiency
Source: J Exp Med. 2022 Sep 12;219(10):e20220094. doi: 10.1084/jem.20220094 (PMC9472563; doi:10.1084/jem.20220094)

**Figure 2**

**A**

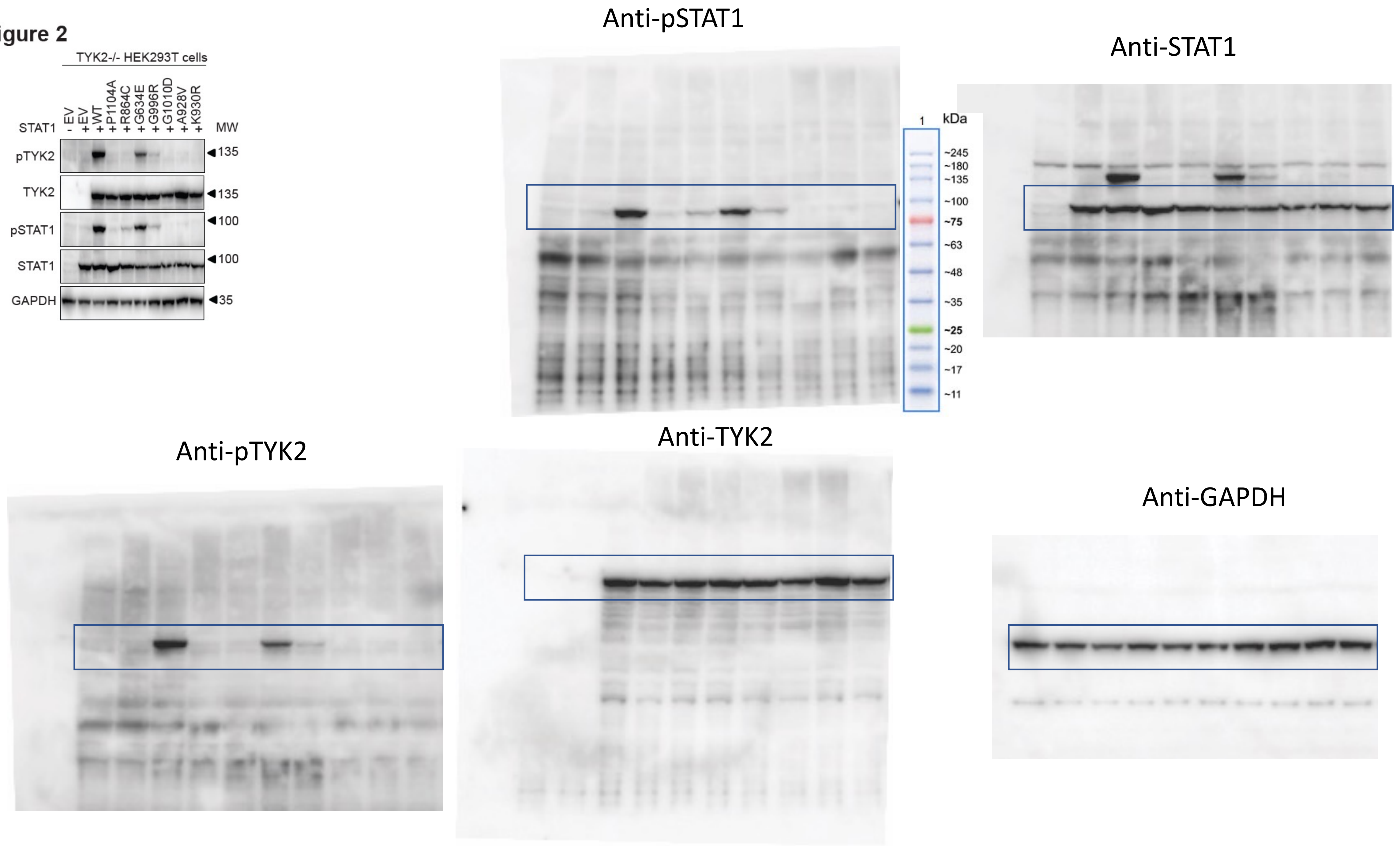
TYK2-/- HEK293T cells
STAT1
- EV
+ EV
+ WT
+ P1104A
+ R864C
+ G634E
+ G996R
+ G1010D
+ A928V
+ K930R
MW
pTYK2
TYK2
pSTAT1
STAT1
GAPDH
135
135
100
100
35
Anti-pSTAT1
1
kDa
~245
~180
~135
~100
~75
~63
~48
~35
~25
~20
~17
~11
Anti-STAT1
Anti-pTYK2
Anti-TYK2
Anti-GAPDH

Anti-pSTAT1

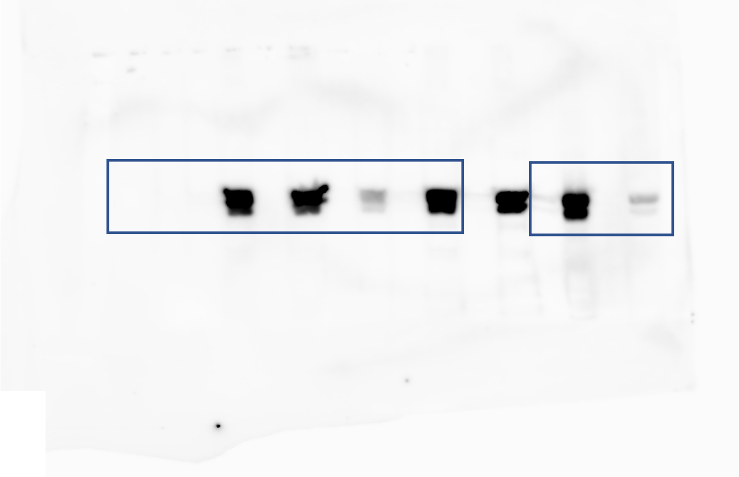

Anti-STAT1

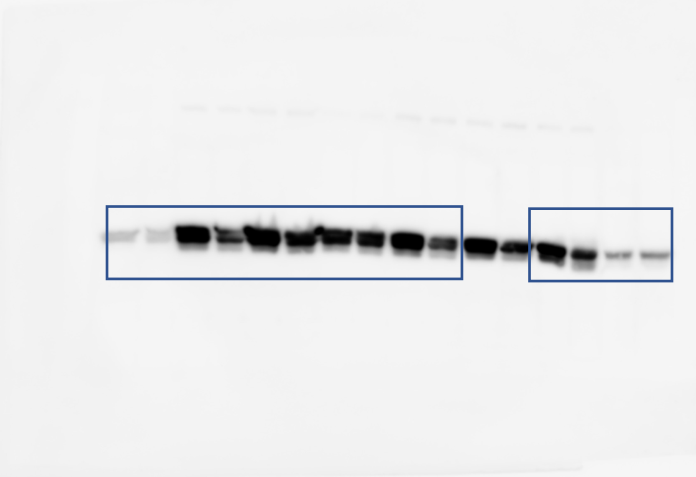

Anti-pSTAT2

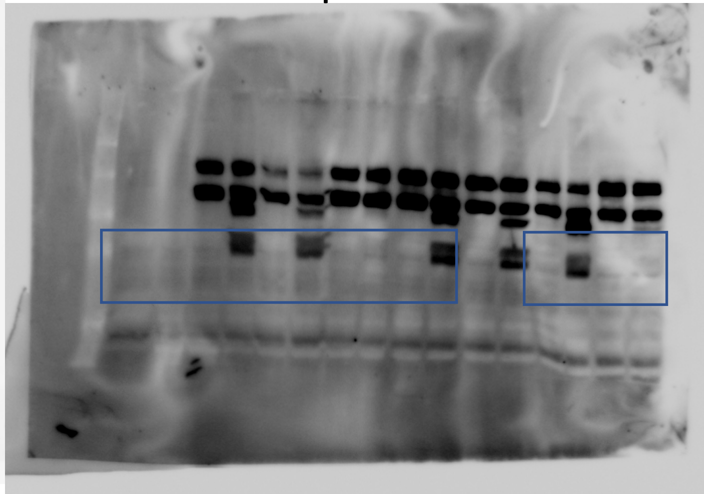

Figure 2D

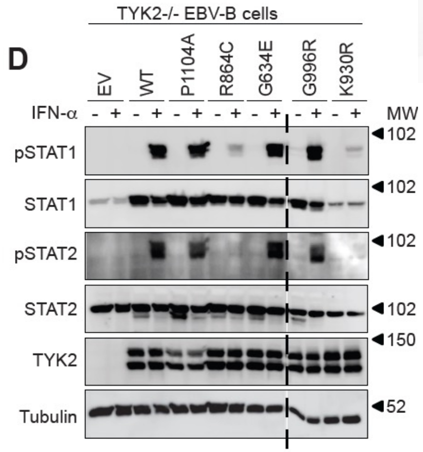


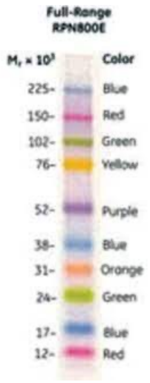


Anti-STAT2

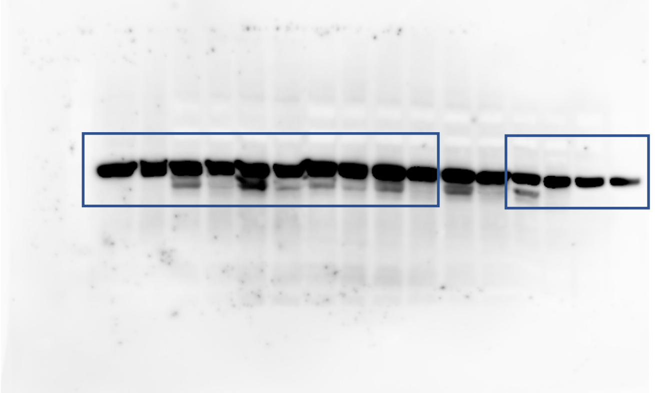

Anti-TYK2

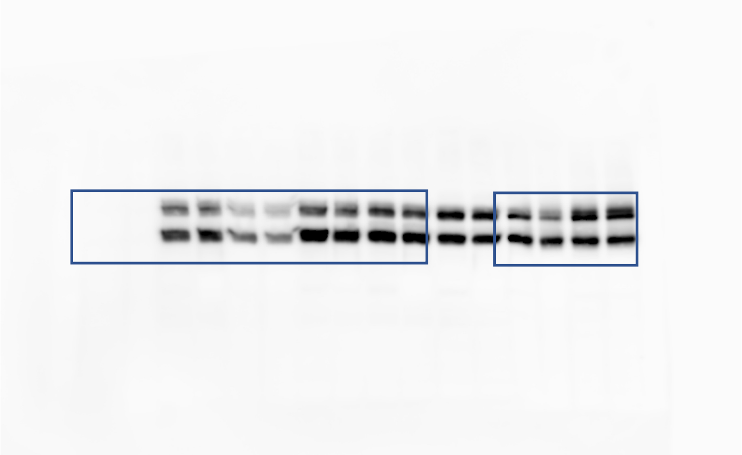

Anti-tubulin

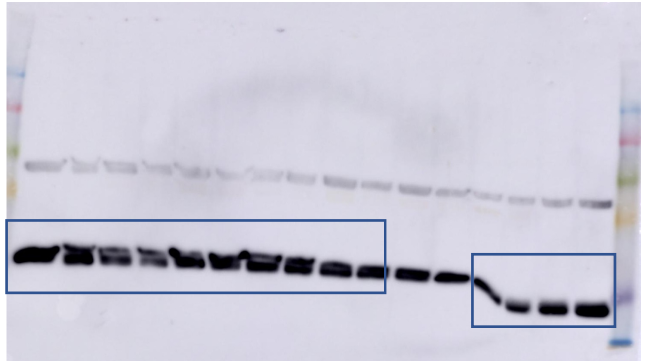

## Figure 2E

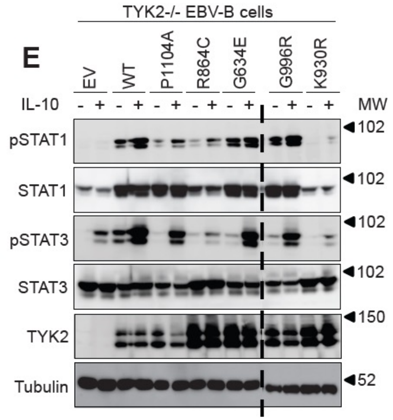


Anti-pSTAT1

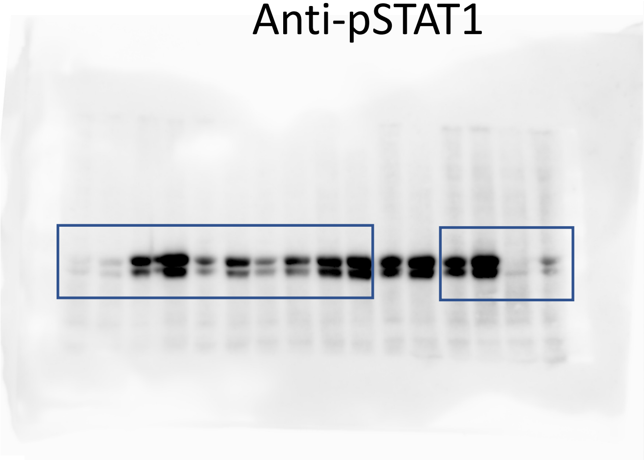

Anti-STAT1

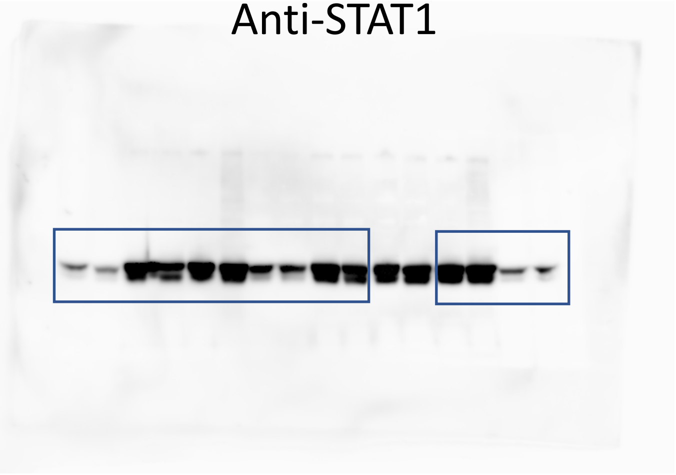

Anti-TYK2

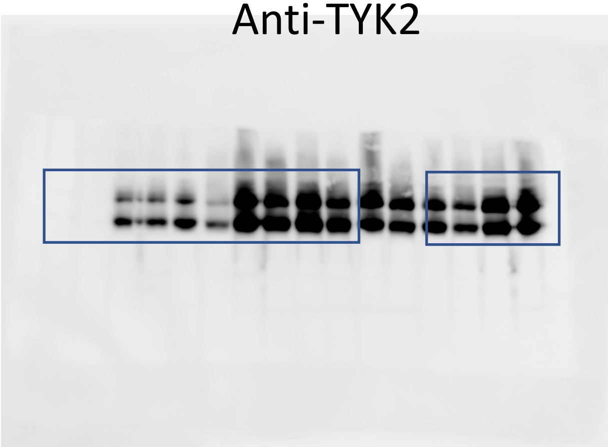

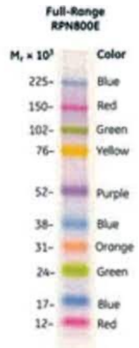


Anti-pSTAT3

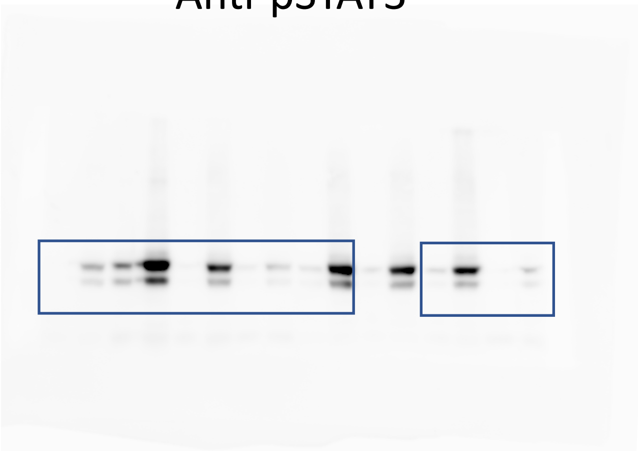

Anti-STAT3

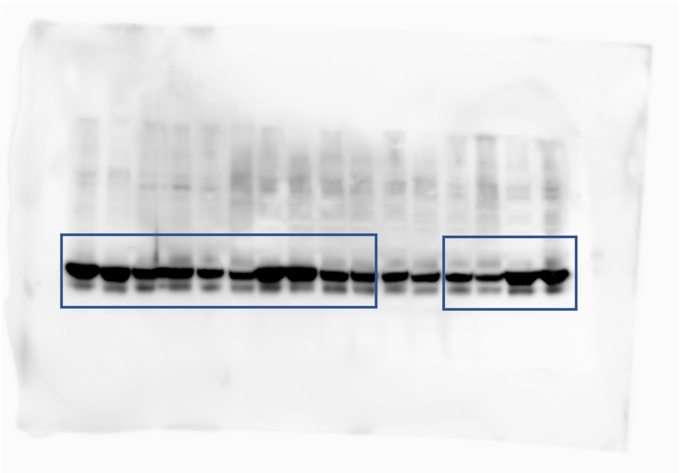

Anti-tubulin

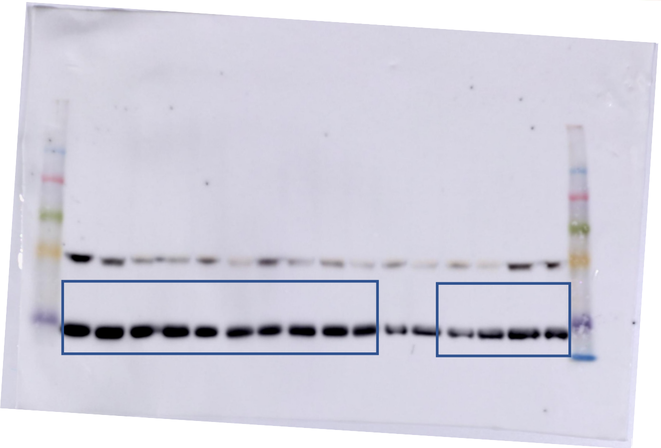

## Figure 2H

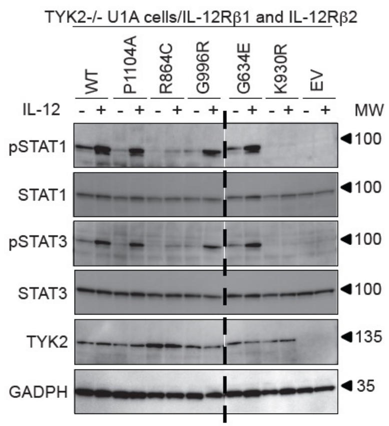
TYK2-/- U1A cells/IL-12Rβ1 and IL-12Rβ2
WT
P1104A
R864C
G996R
G634E
K930R
EV
IL-12
MW
pSTAT1
STAT1
pSTAT3
STAT3
TYK2
GADPH
100
100
100
100
135
35

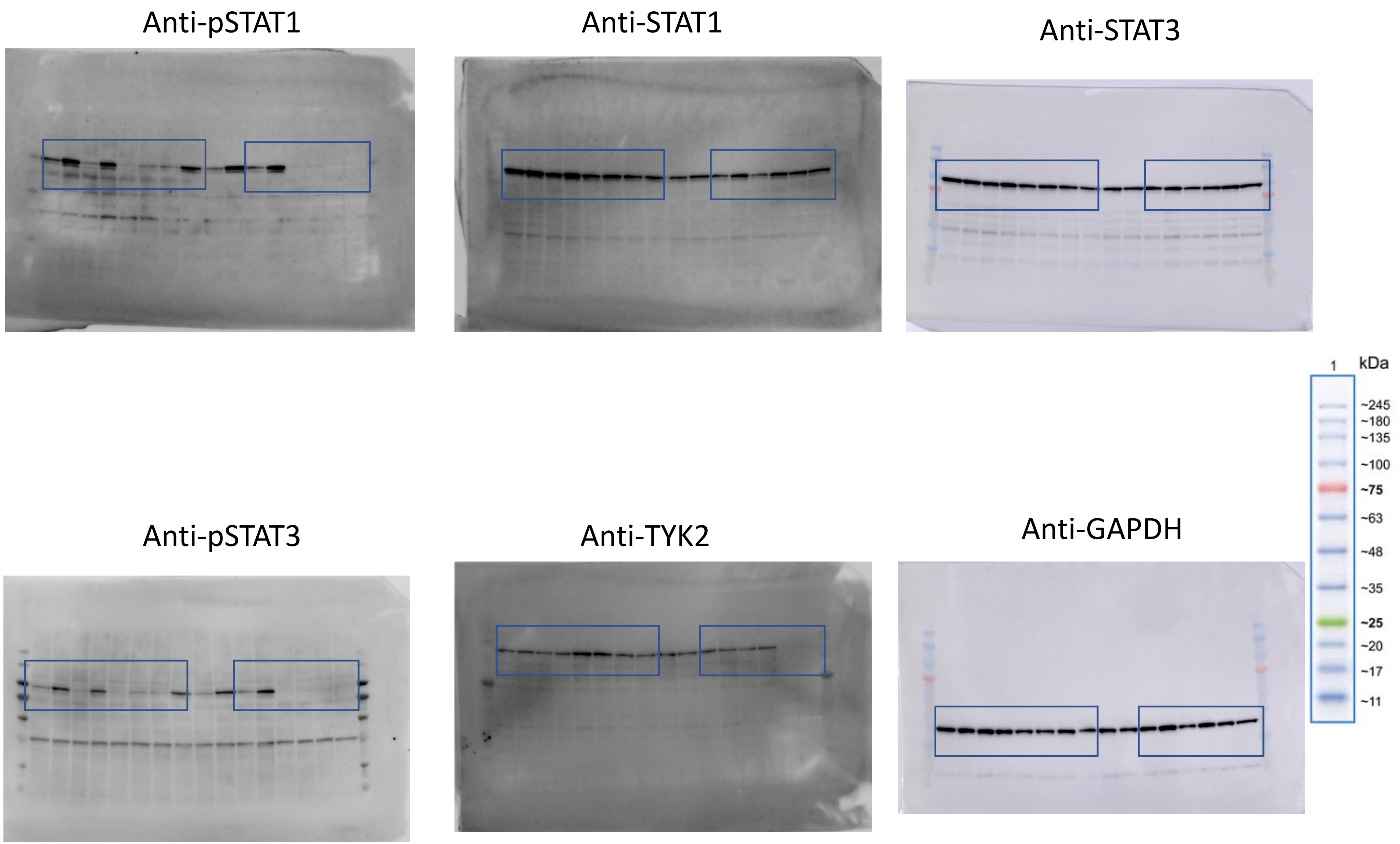
Anti-pSTAT1
Anti-STAT1
Anti-STAT3
Anti-pSTAT3
Anti-TYK2
Anti-GAPDH
1
kDa
~245
~180
~135
~100
~75
~63
~48
~35
~25
~20
~17
~11

## Figure 2I

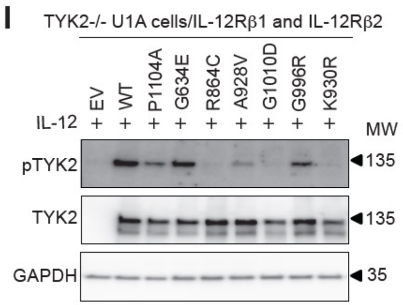


Anti-pTYK2

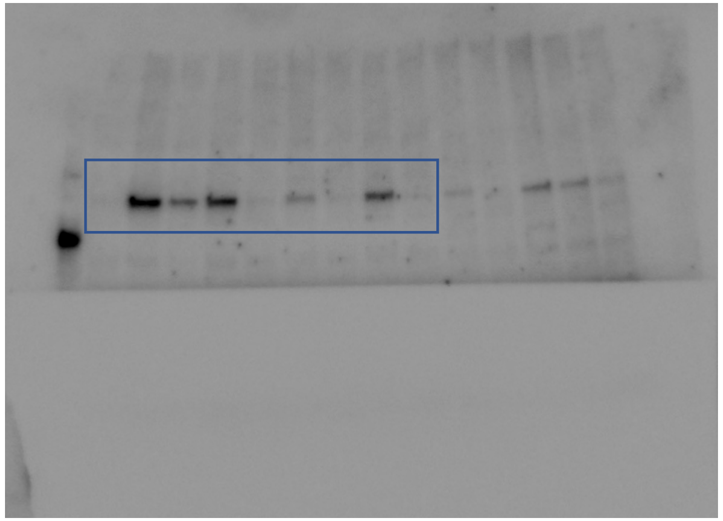

Anti-TYK2

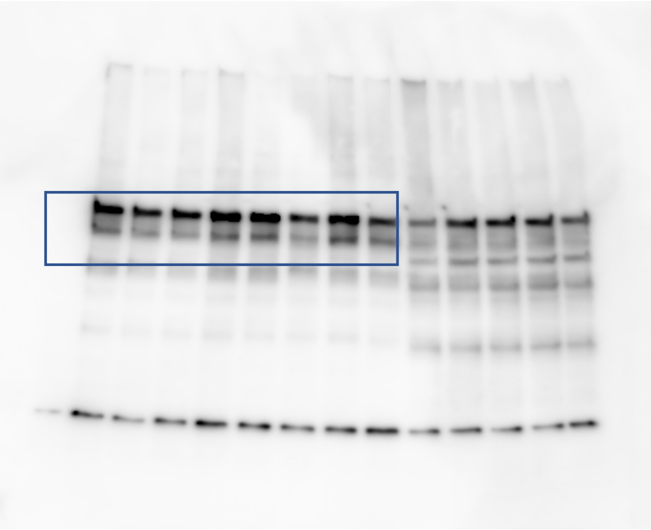

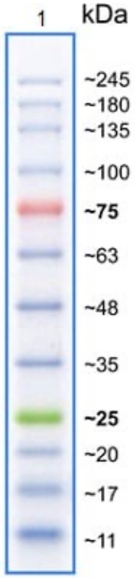


Anti-GAPDH

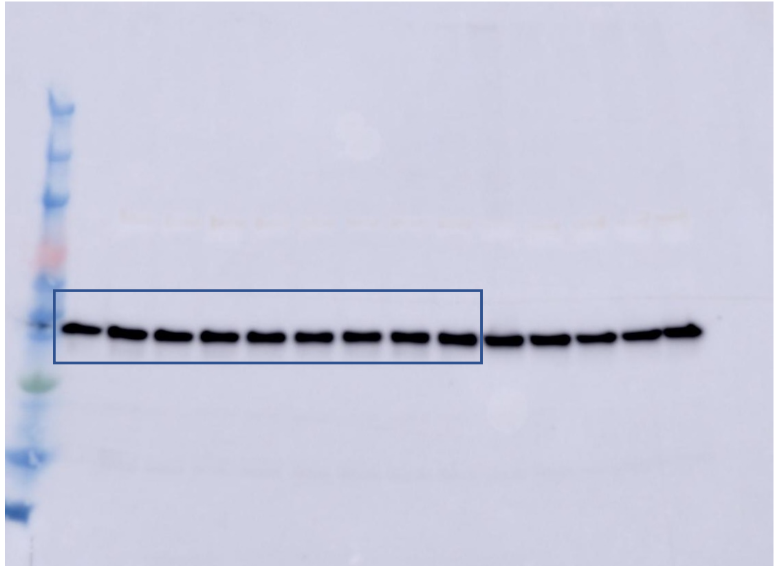

# Figure 2K left

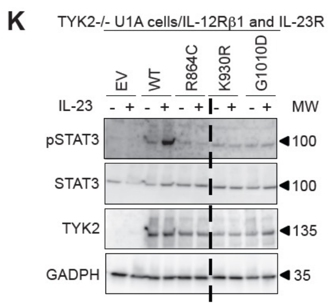


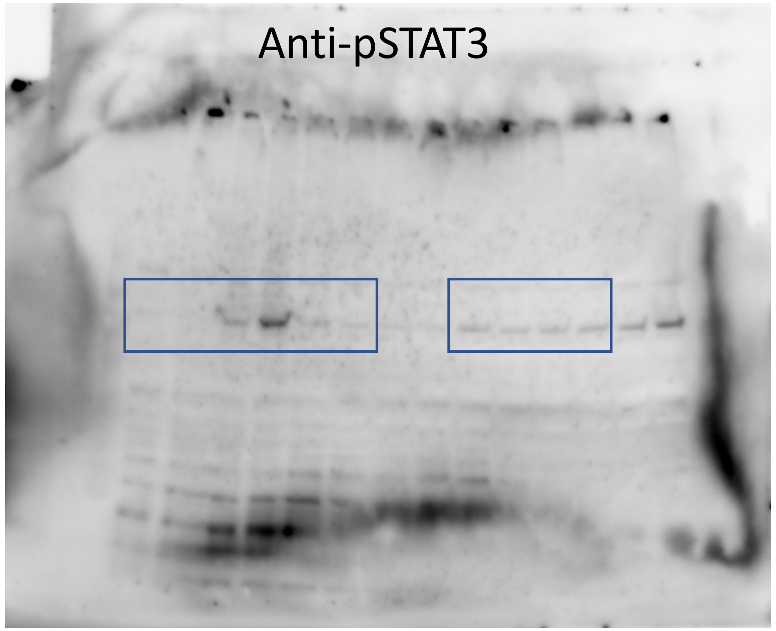


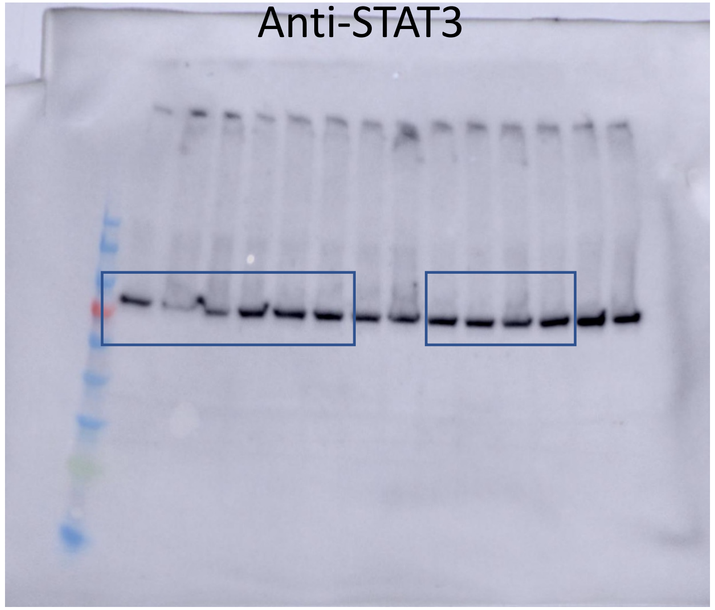


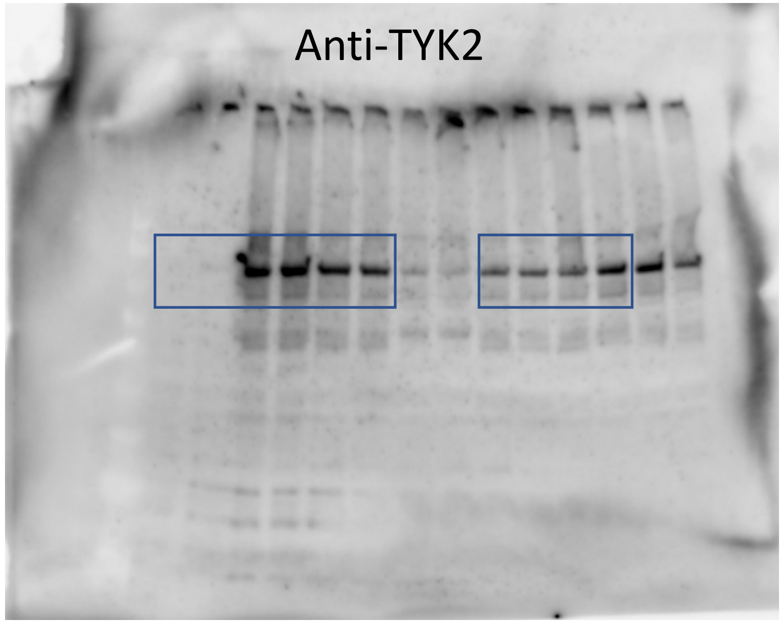


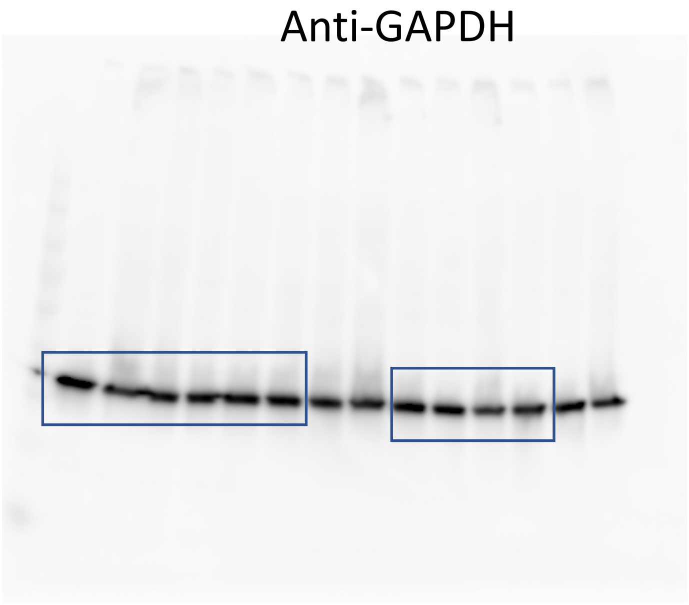


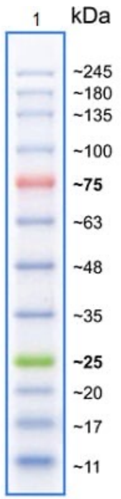

# Figure 2K right

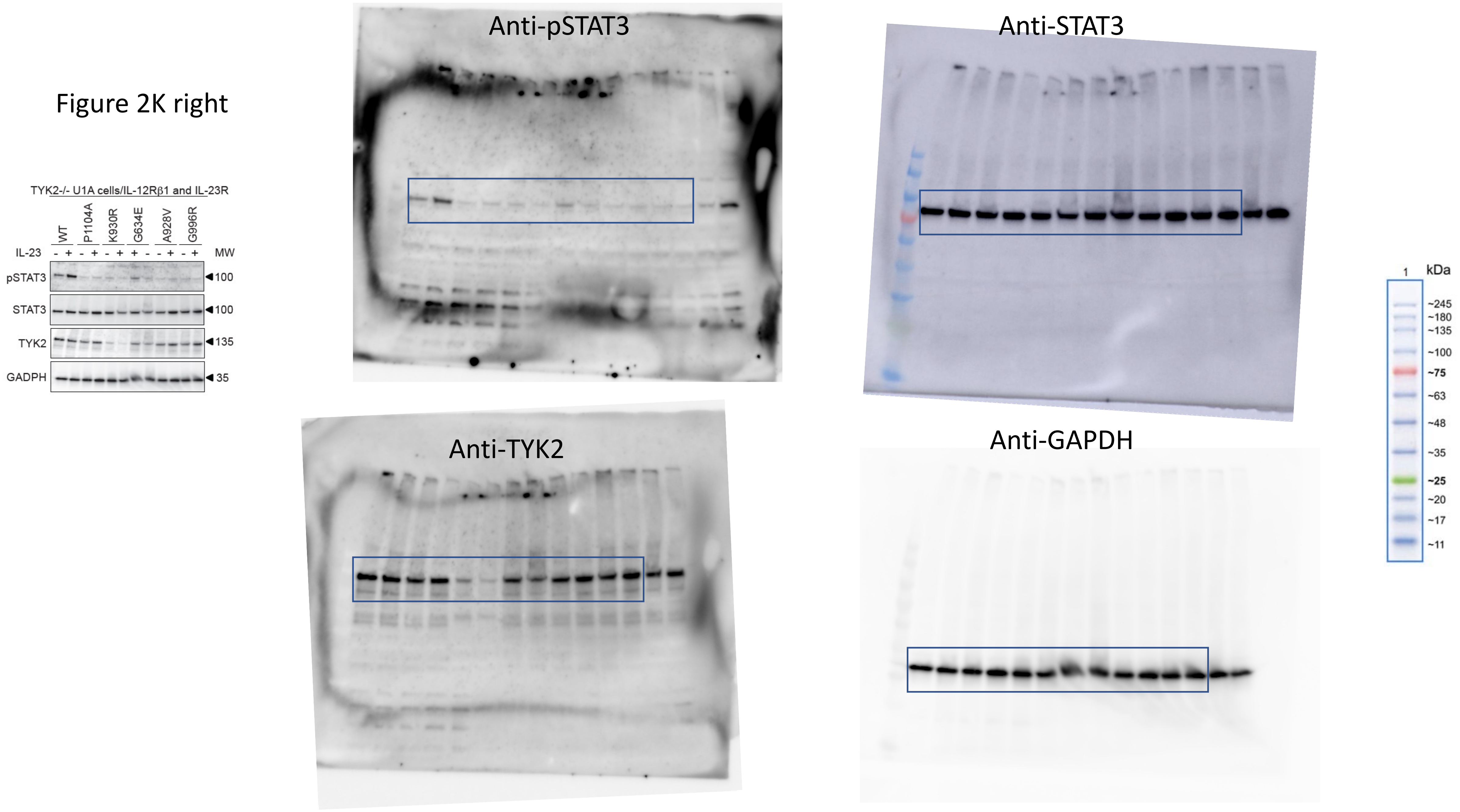

Supplement: SourceData F2 — contains original blots for Fig. 2. [file JEM_20220094_SourceDataF2.pdf]
