# Supplementary material for: Impaired IL-23–dependent induction of IFN-γ underlies mycobacterial disease in patients with inherited TYK2 deficiency
Source: J Exp Med. 2022 Sep 12;219(10):e20220094. doi: 10.1084/jem.20220094 (PMC9472563; doi:10.1084/jem.20220094)

Figure 3

A

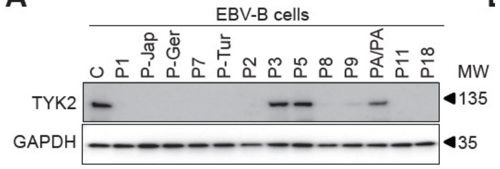

Anti-TYK2

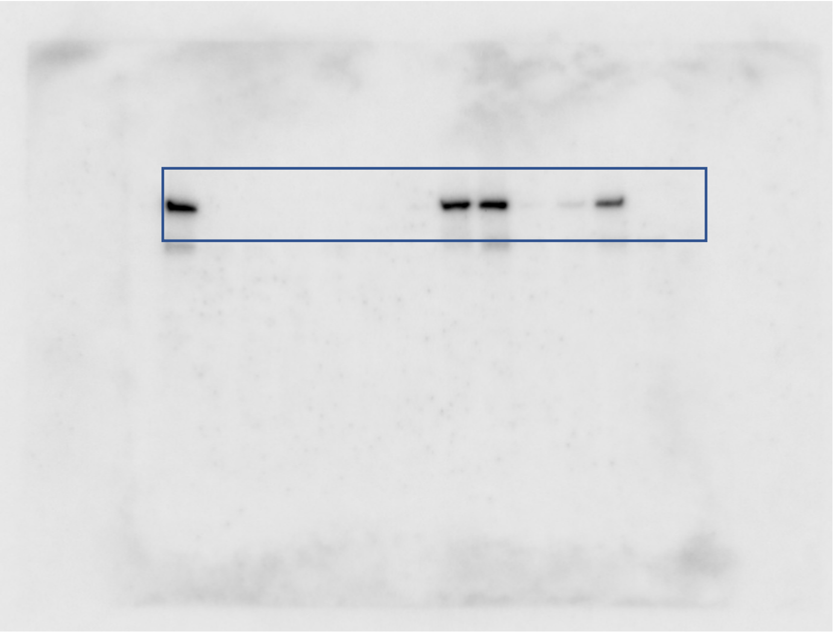

Anti-GAPDH

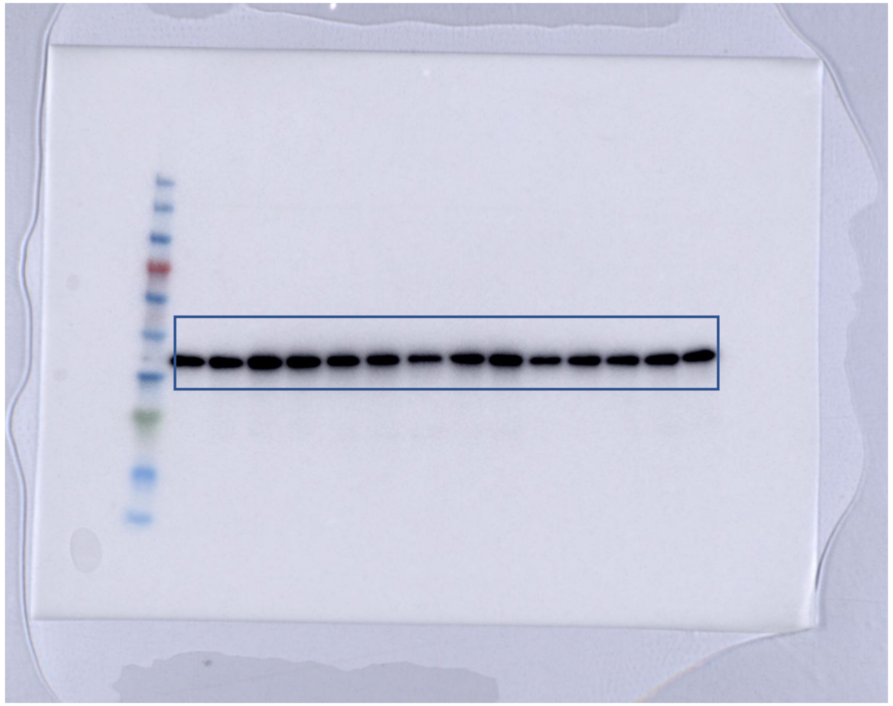

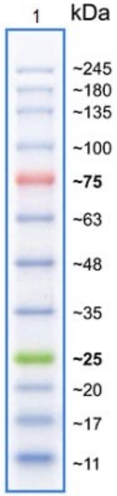

Figure 3C left

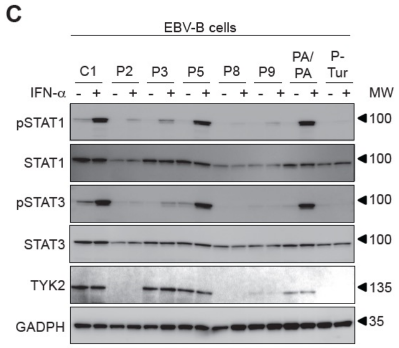

Anti-pSTAT1

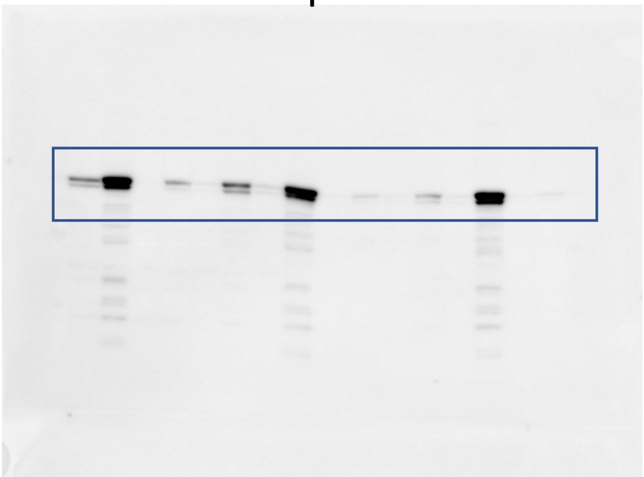

Anti-STAT1

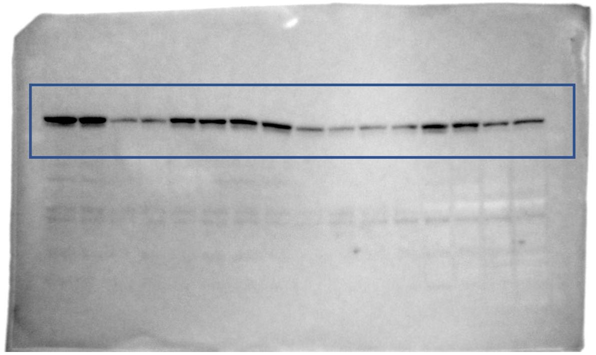

Anti-TYK2

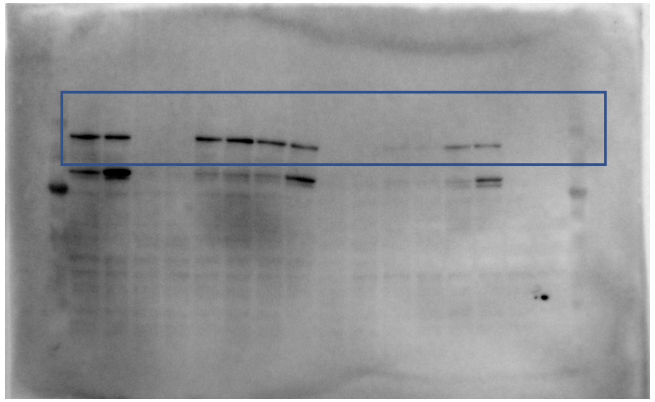

Anti-pSTAT3

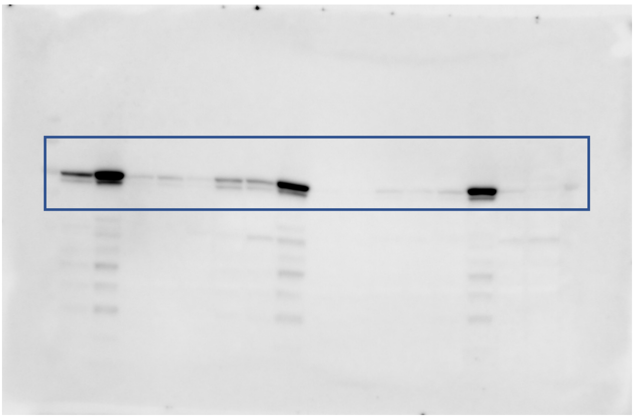

Anti-STAT3

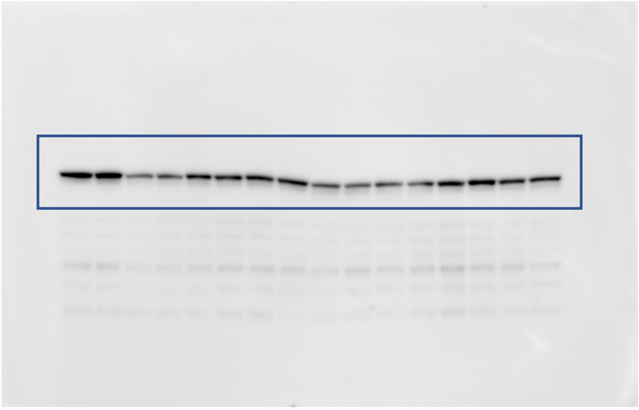

Anti-GAPDH

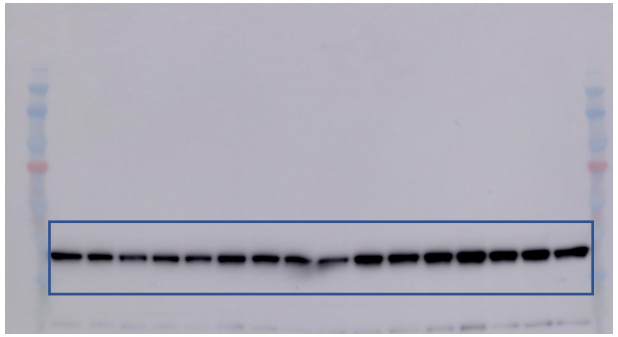

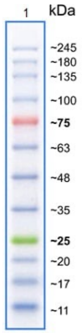

Figure 3C middle

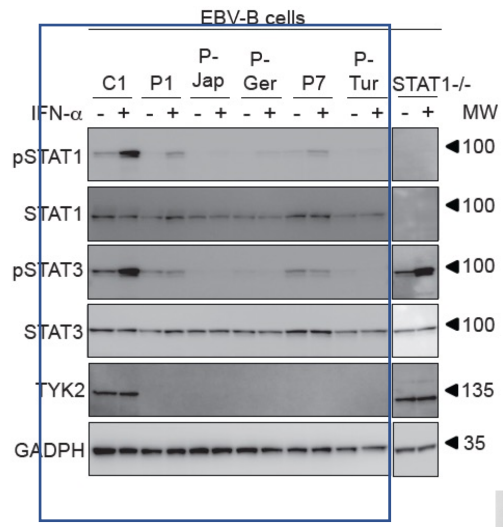

Anti-pSTAT1

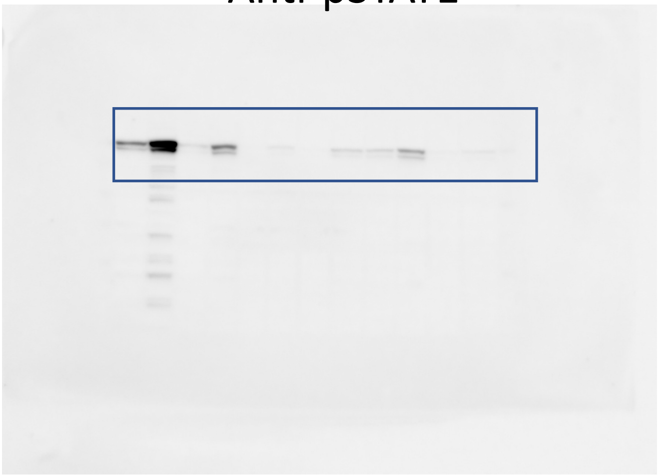

Anti-STAT1

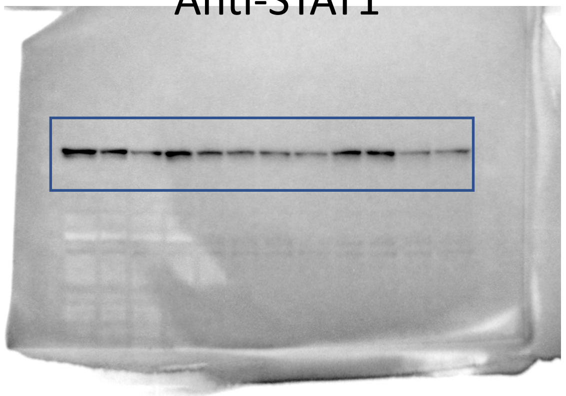

Anti-TYK2

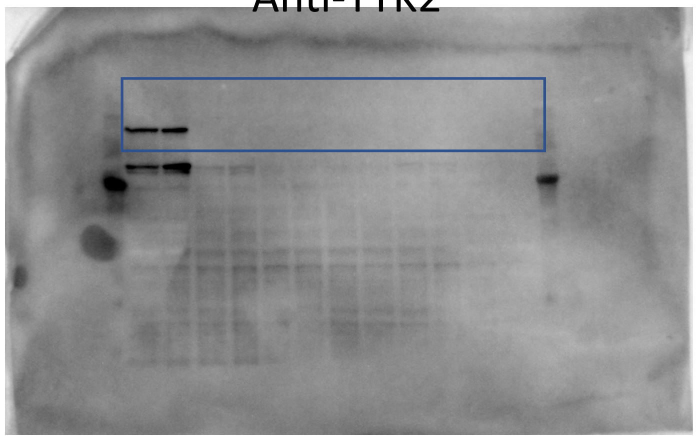

Anti-pSTAT3

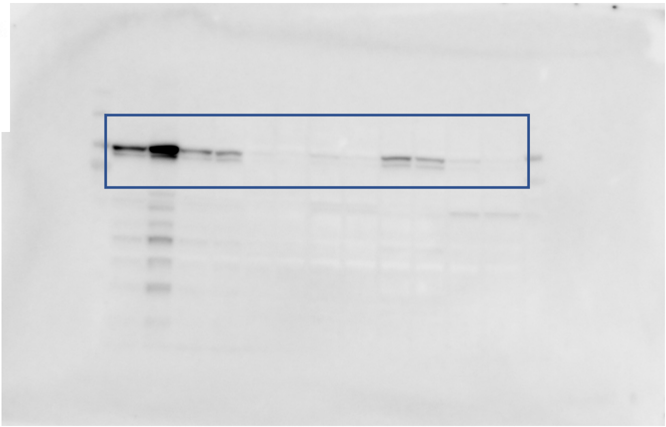

Anti-STAT3

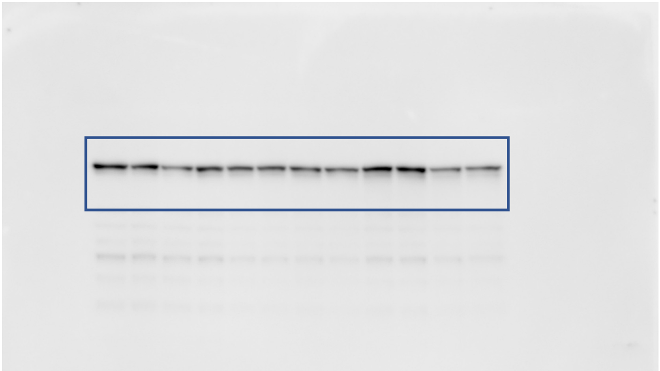

Anti-GAPDH

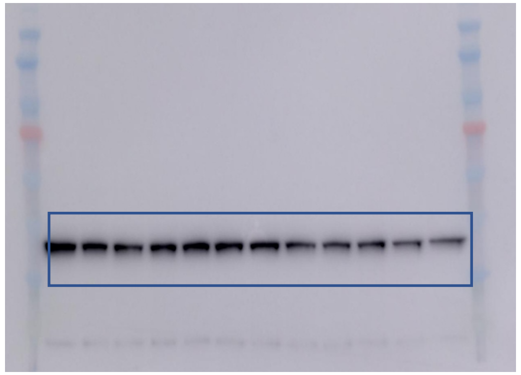

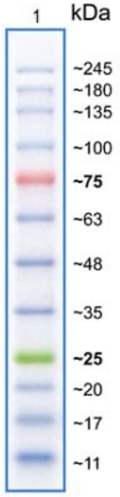

Figure 3C middle

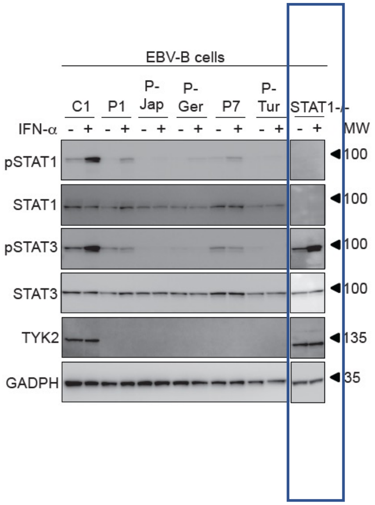

Figure 3D middle

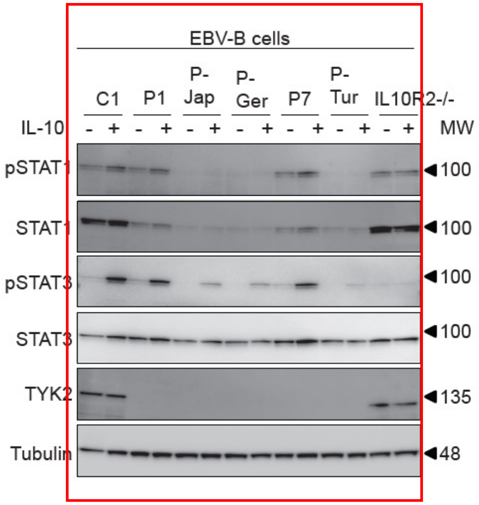

Anti-pSTAT1

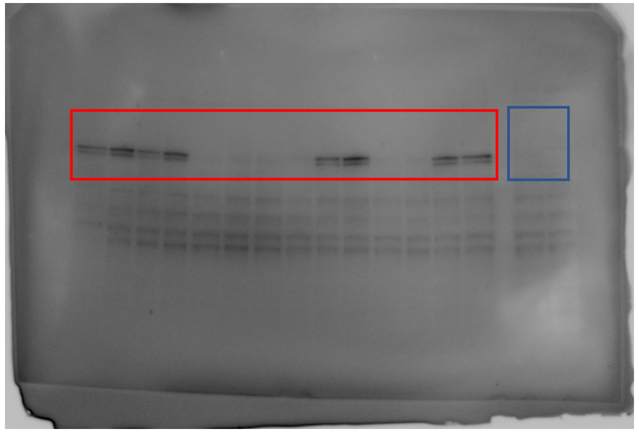

Anti-STAT1

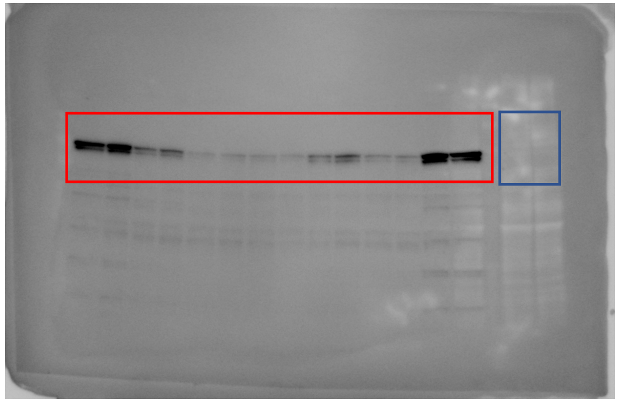

Anti-TYK2

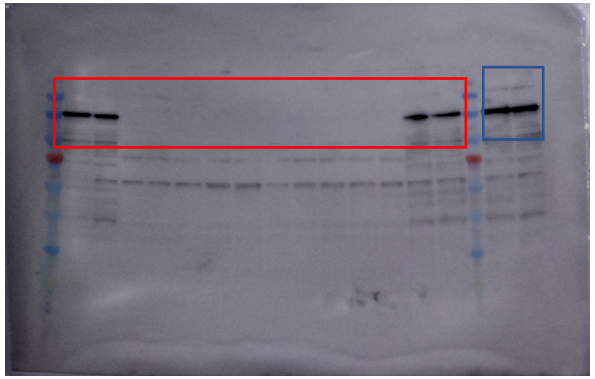

Anti-pSTAT3

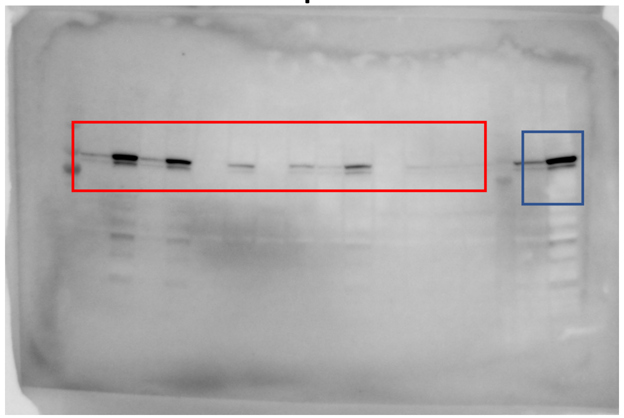

Anti-STAT3

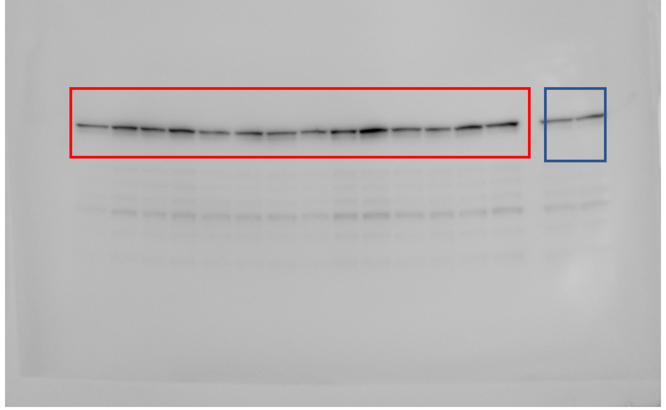

Anti-GAPDH

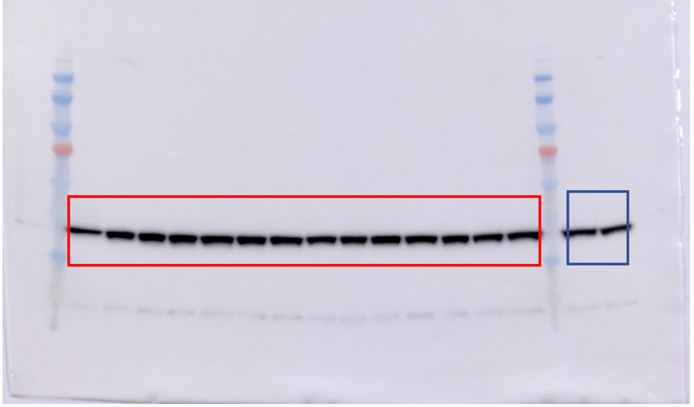

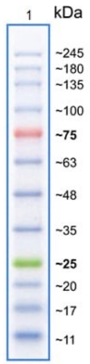

Anti-pSTAT1

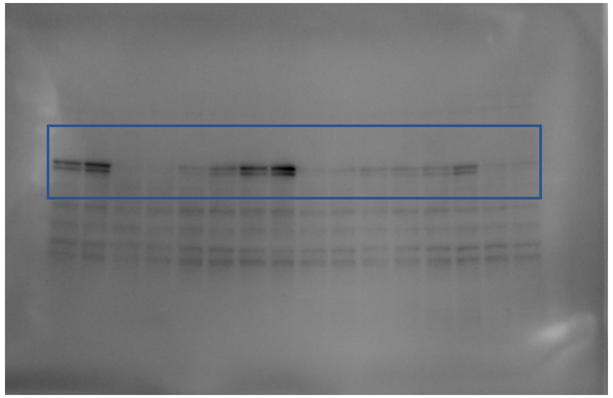

Anti-STAT1

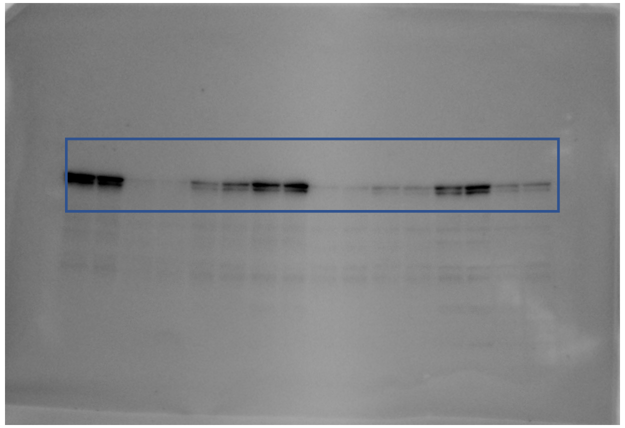

Anti-TYK2

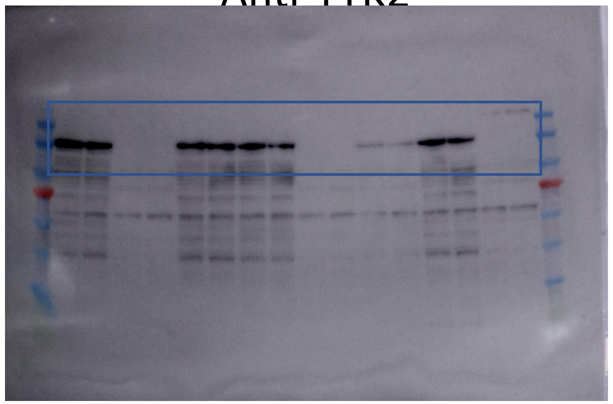

Figure 3D left

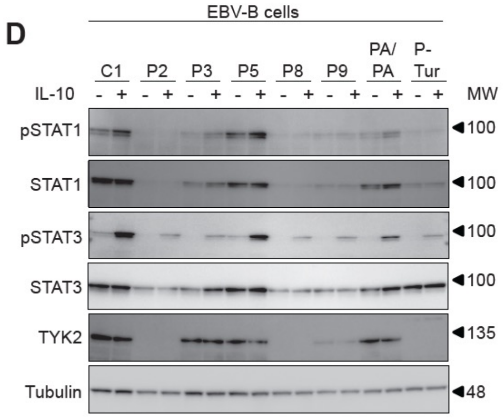

Anti-pSTAT3

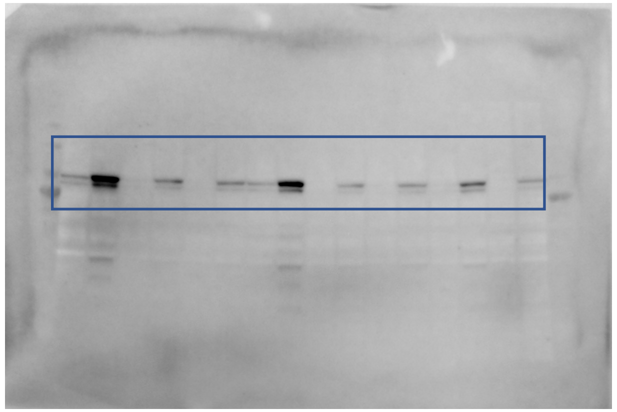

Anti-STAT3

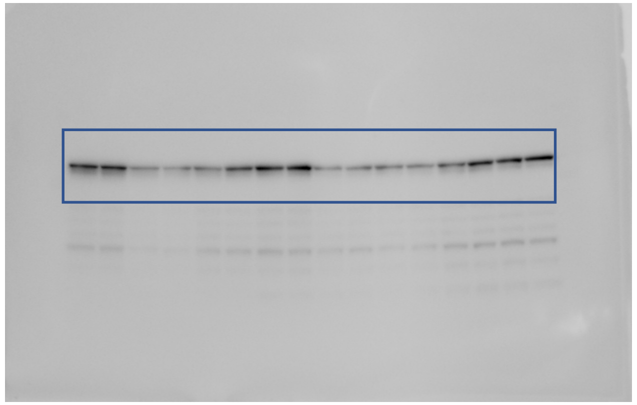

Anti-GAPDH

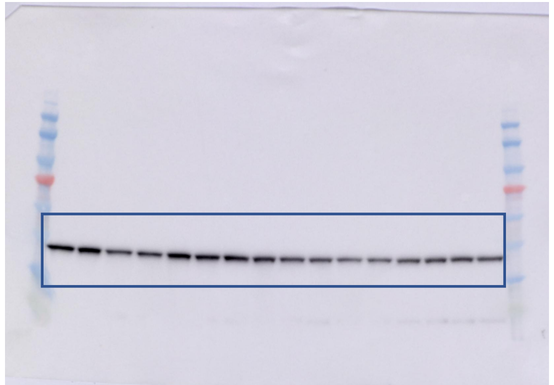

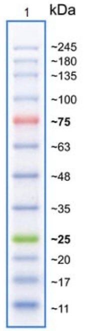

Figure 3C right

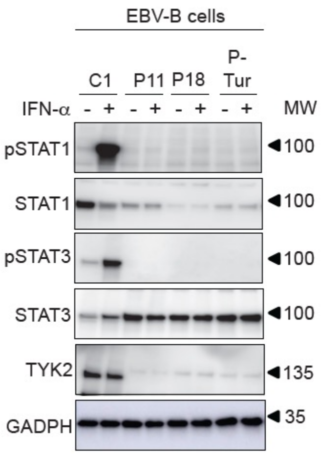

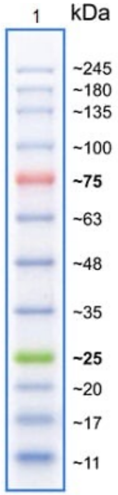

Anti-pSTAT1

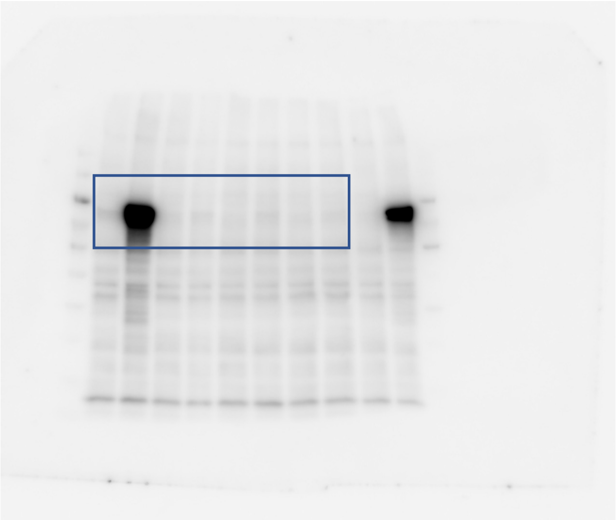

Anti-STAT1

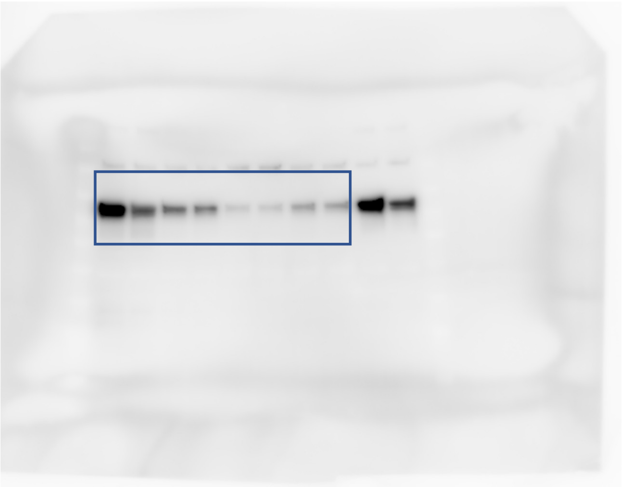

Anti-TYK2

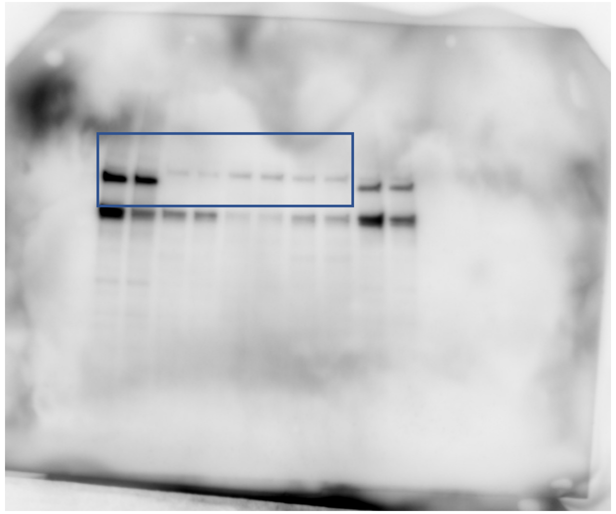

Anti-pSTAT3

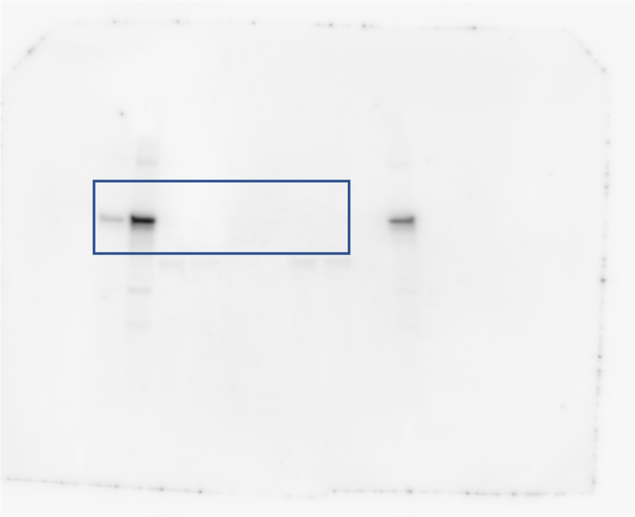

Anti-STAT3

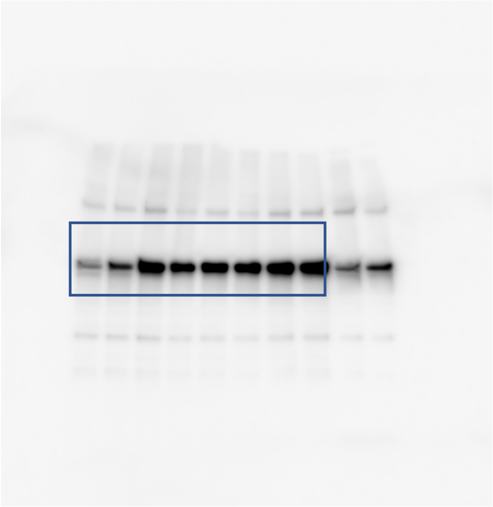

Anti-GAPDH

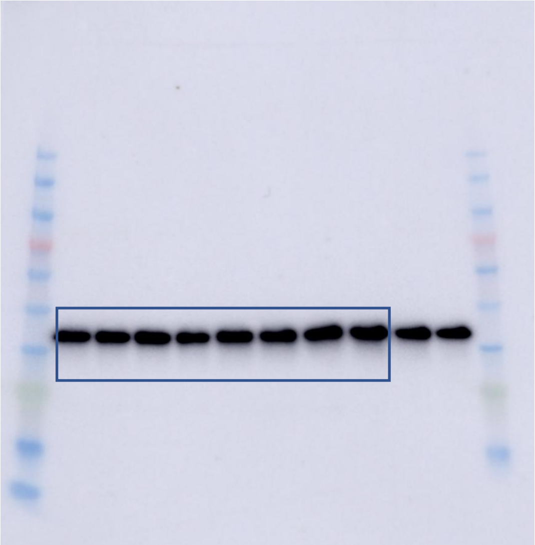

Figure 3D right

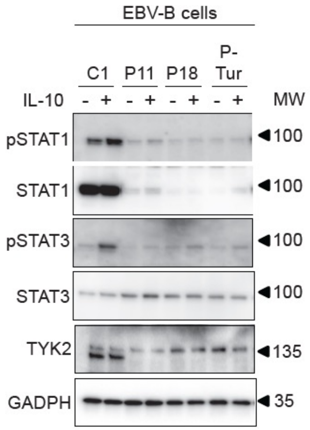

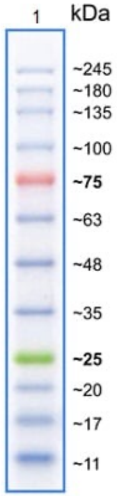

Anti-pSTAT1

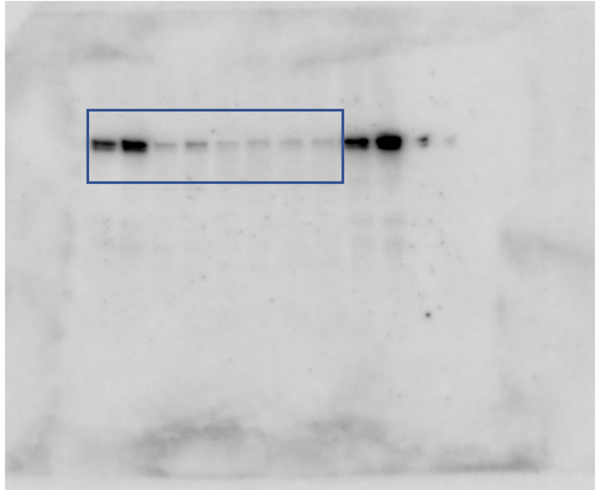

Anti-STAT1

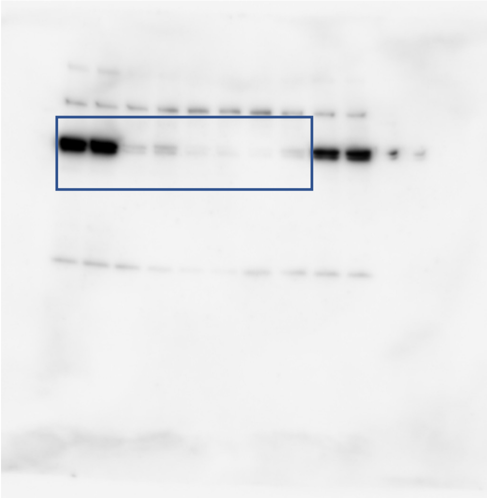

Anti-TYK2

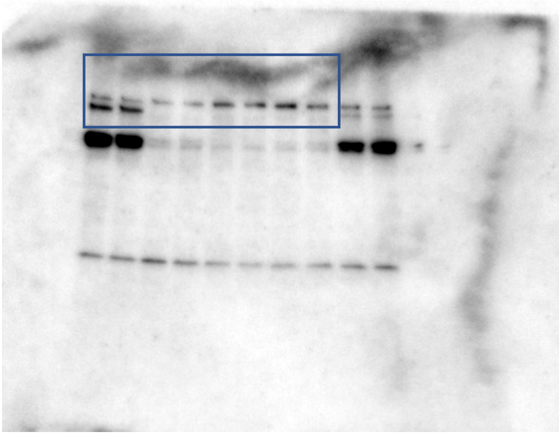

Anti-pSTAT3

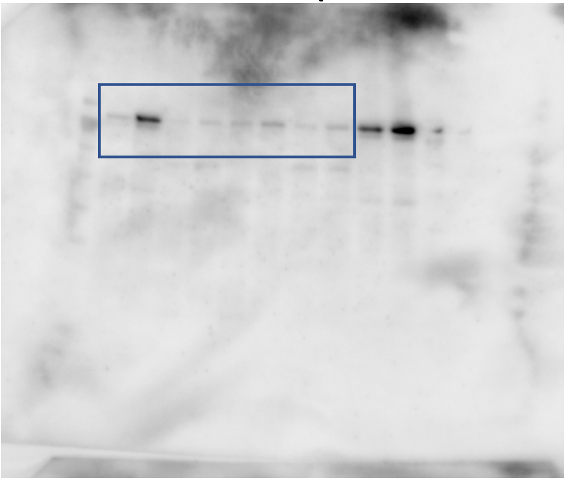

Anti-STAT3

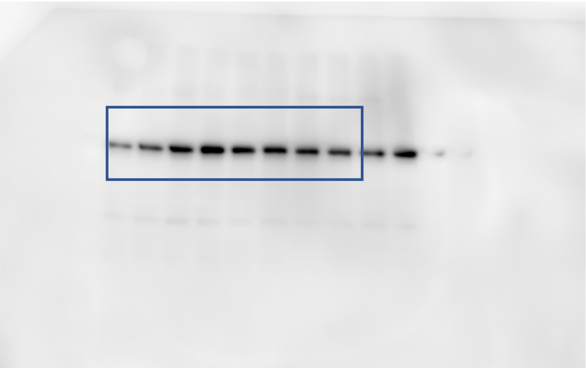

Anti-GAPDH

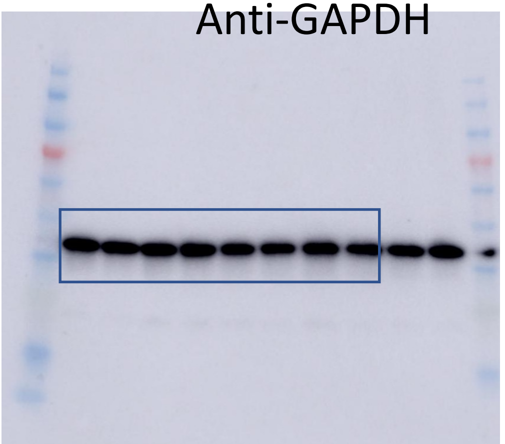

Figure 3G left

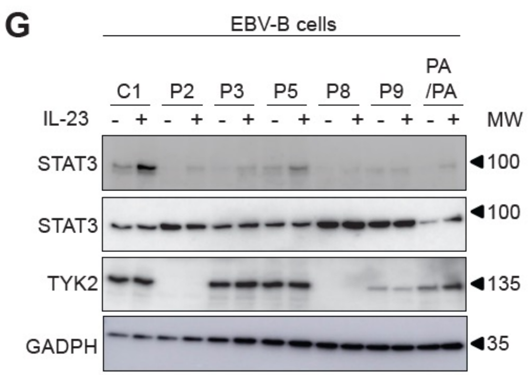

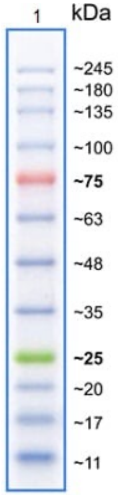

Anti-pSTAT3

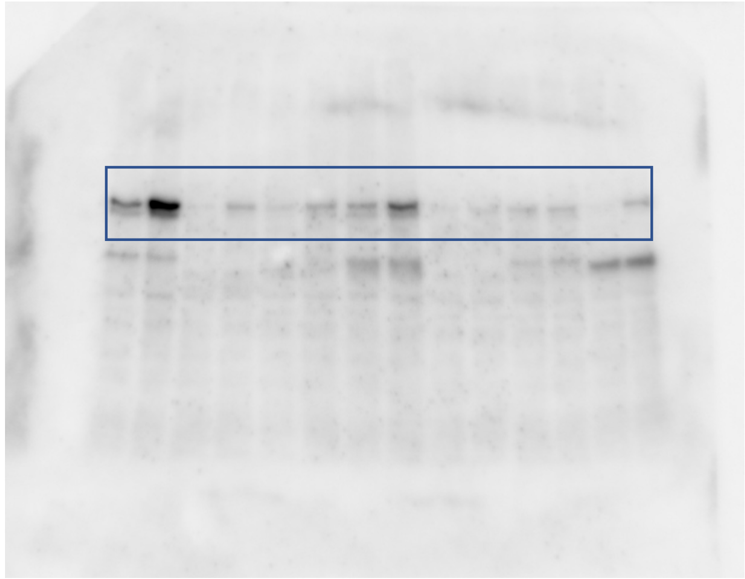

Anti-STAT3

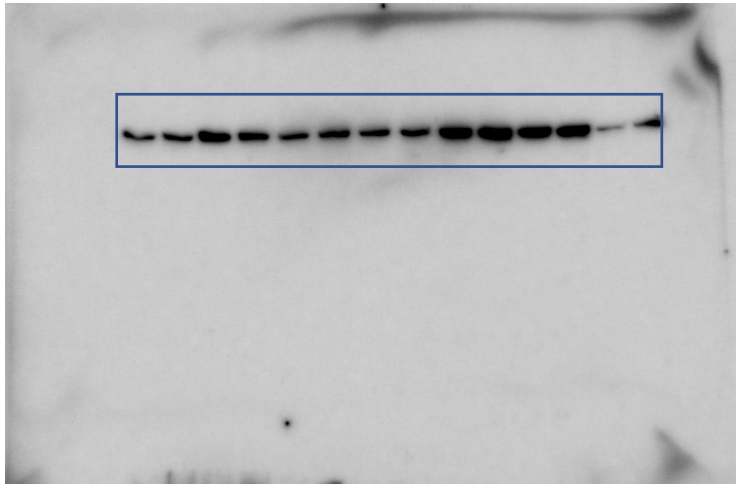

Anti-TYK2

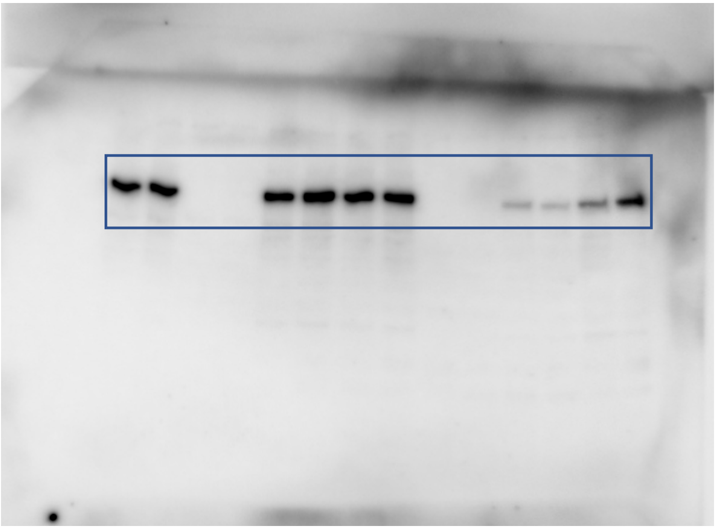

Anti-GAPDH

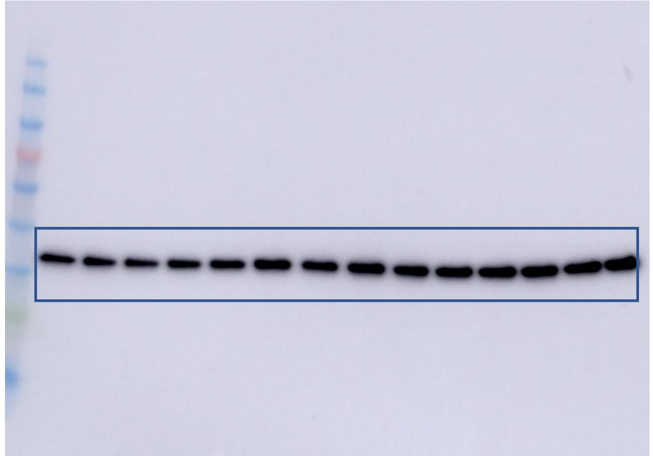

Figure 3G middle

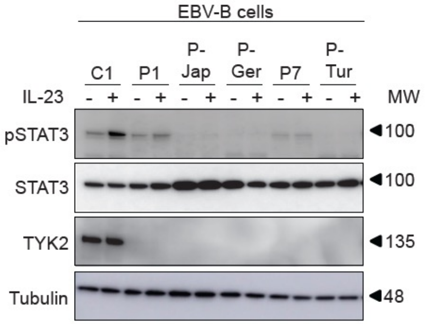

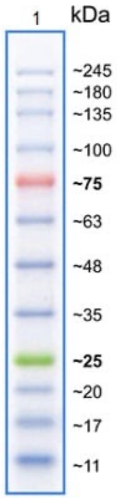

Anti-pSTAT3

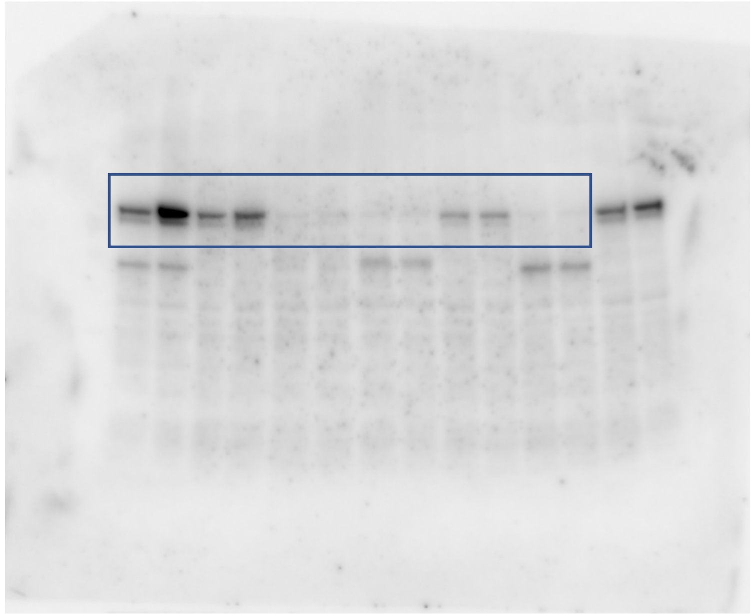

Anti-STAT3

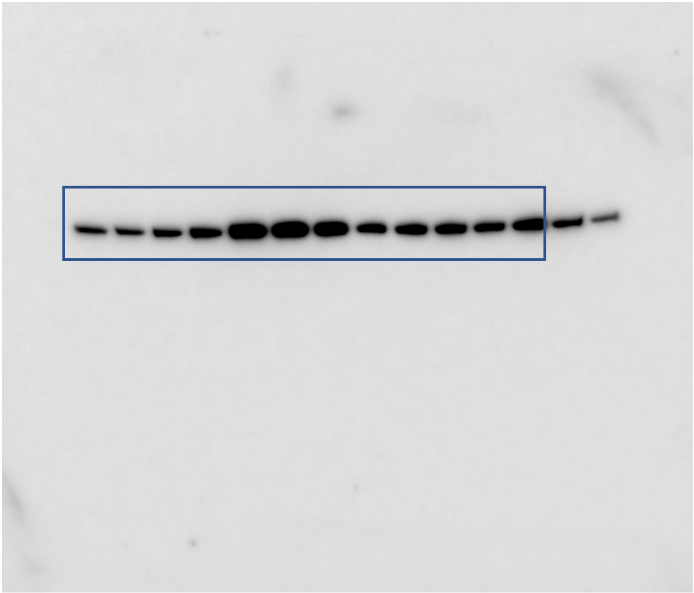

Anti-TYK2

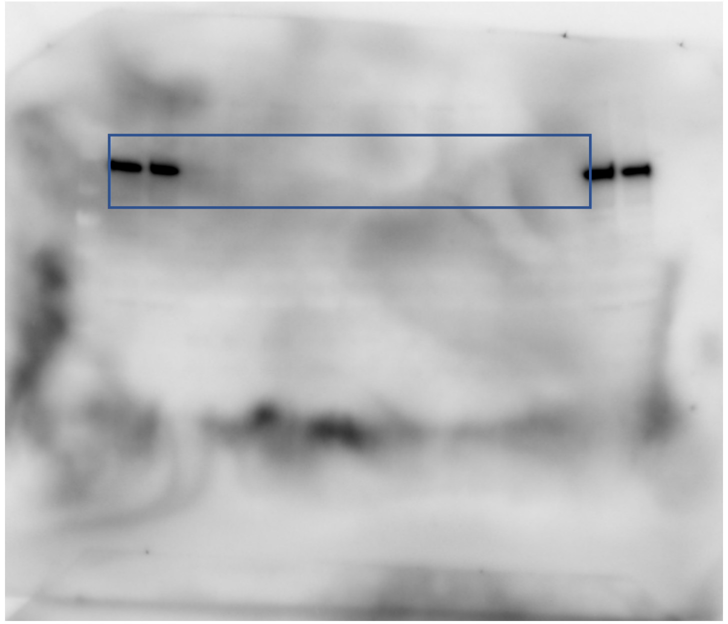

Anti-GAPDH

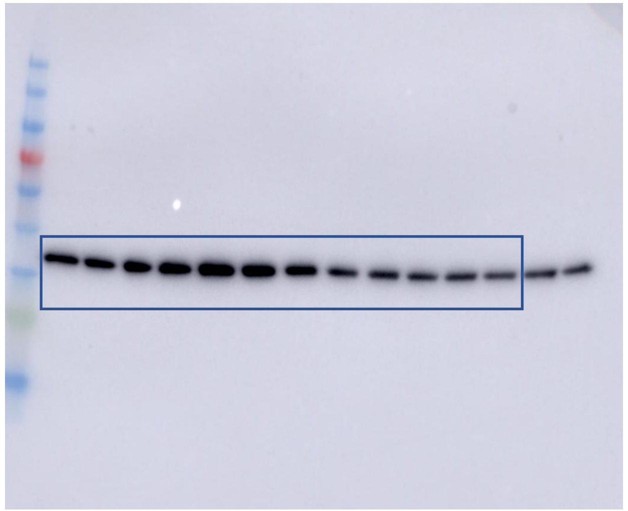

Figure 3G right

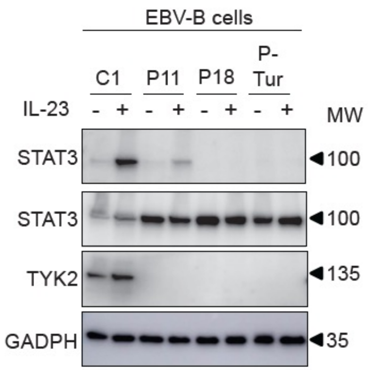

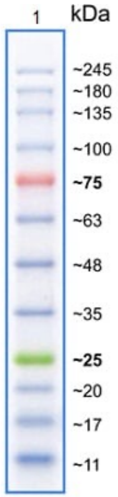

Anti-pSTAT3

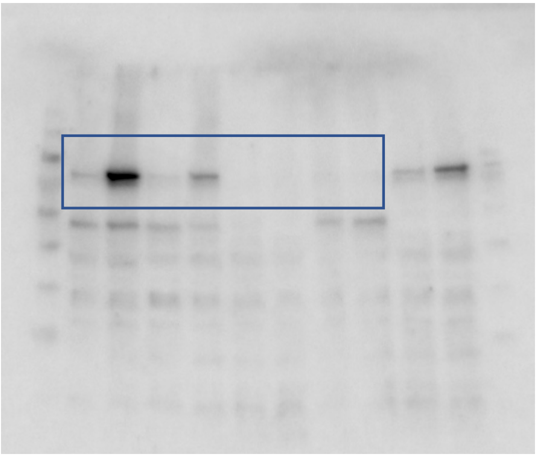

Anti-TYK2

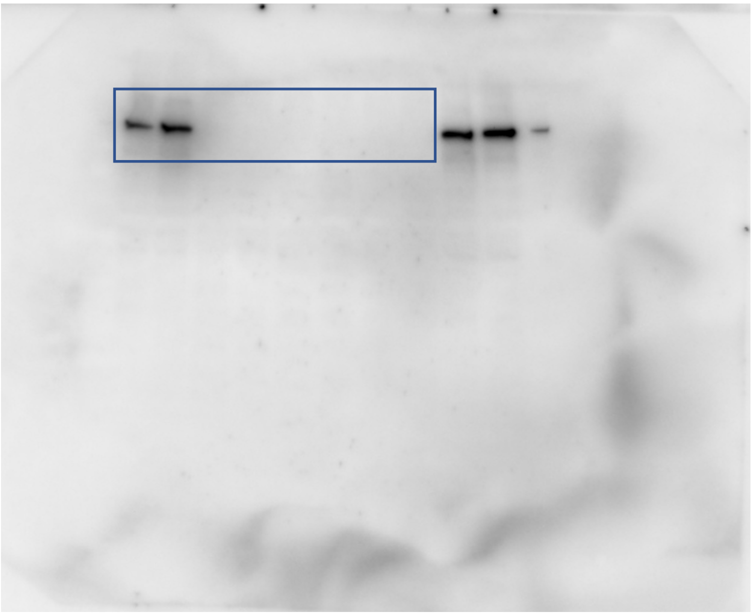

Anti-STAT3

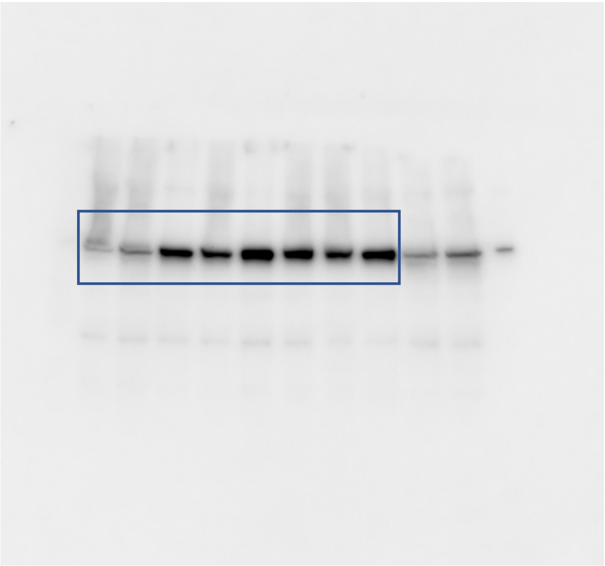

Anti-GAPDH

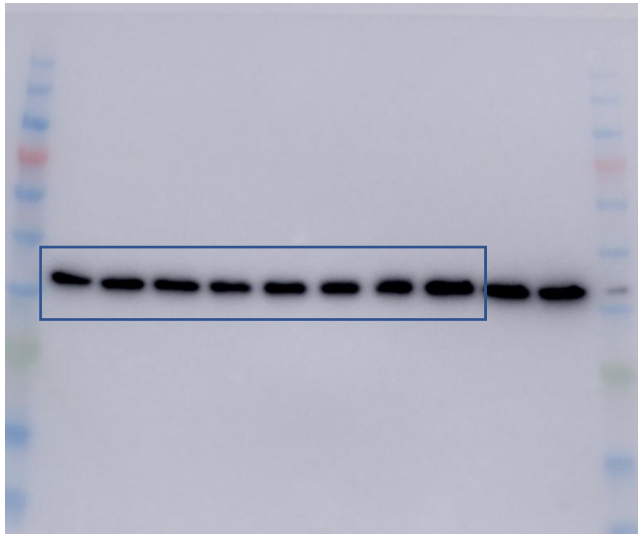

Supplement: SourceData F3 — contains original blots for Fig. 3. [file JEM_20220094_SourceDataF3.pdf]
