# Supplementary material for: Impaired IL-23–dependent induction of IFN-γ underlies mycobacterial disease in patients with inherited TYK2 deficiency
Source: J Exp Med. 2022 Sep 12;219(10):e20220094. doi: 10.1084/jem.20220094 (PMC9472563; doi:10.1084/jem.20220094)

Figure 5A left

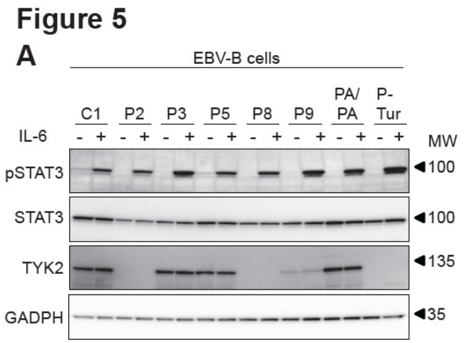

Anti-pSTAT3

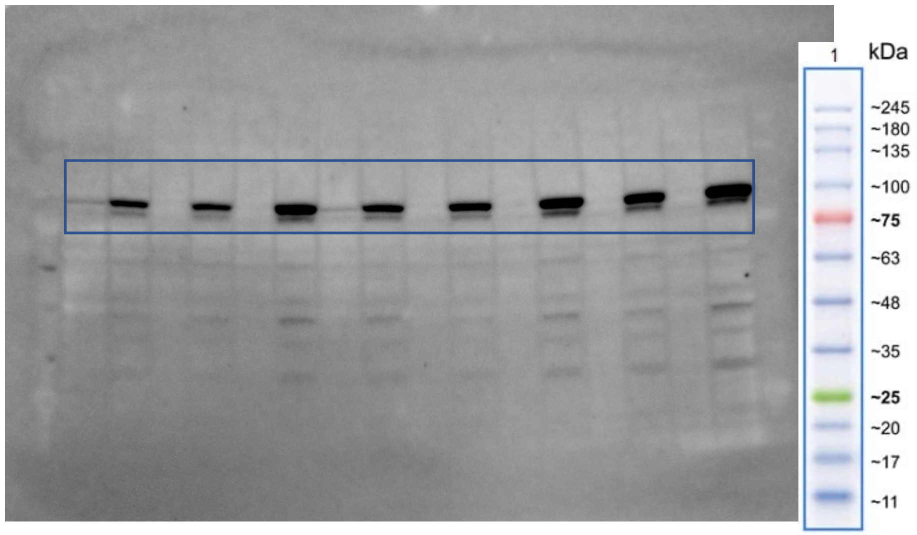

Anti-STAT3

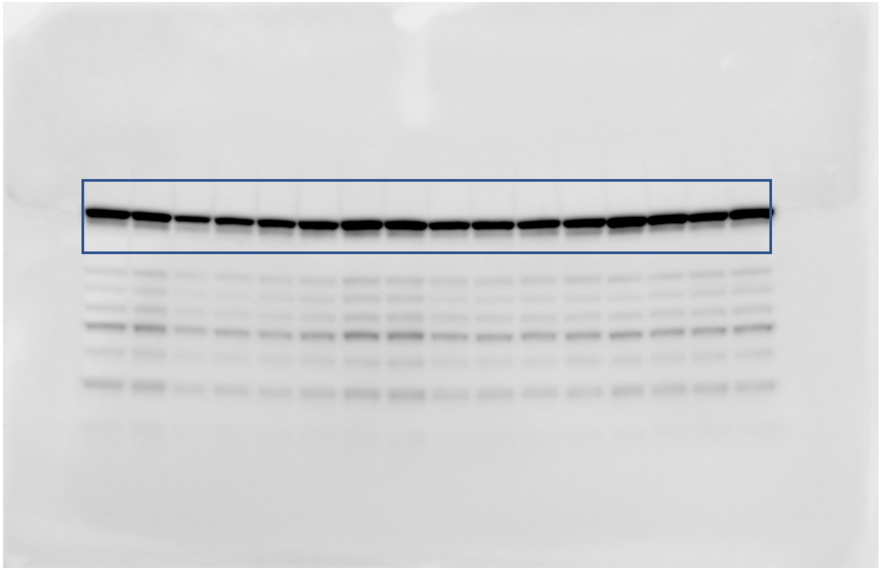

Anti-TYK2

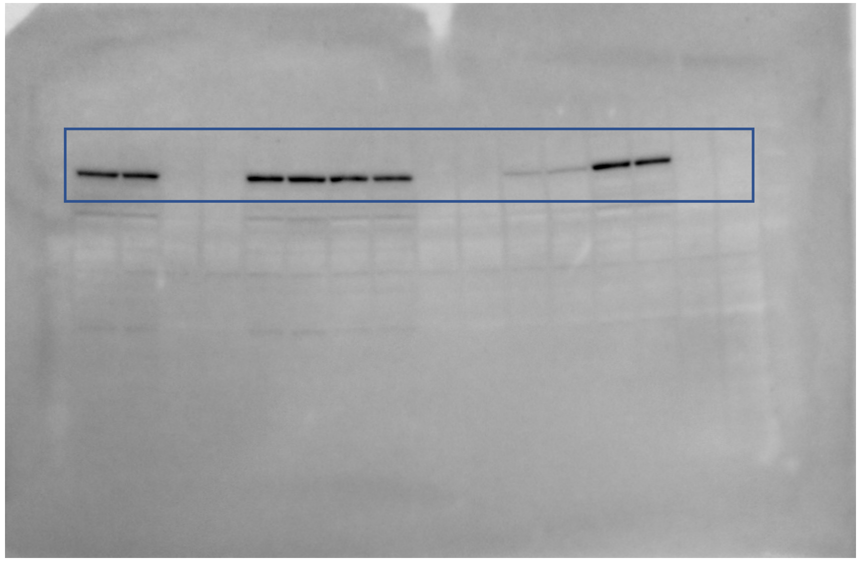

Anti-GAPDH

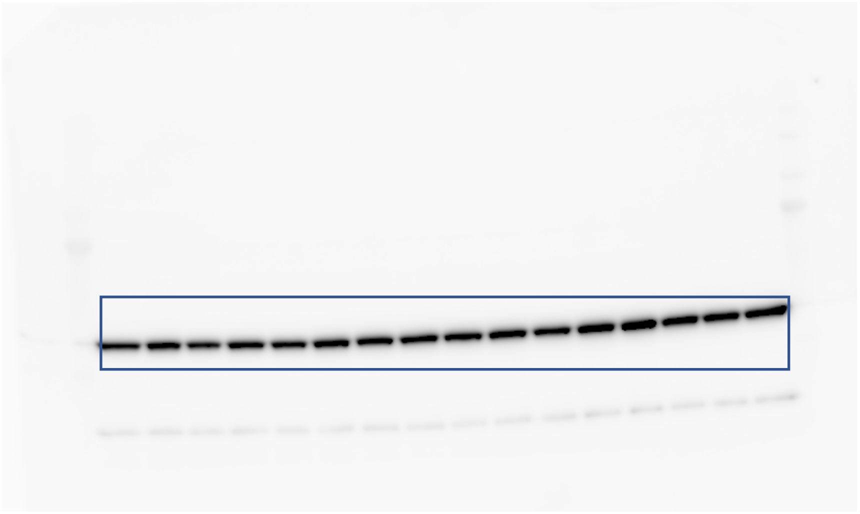

Figure 5A middle

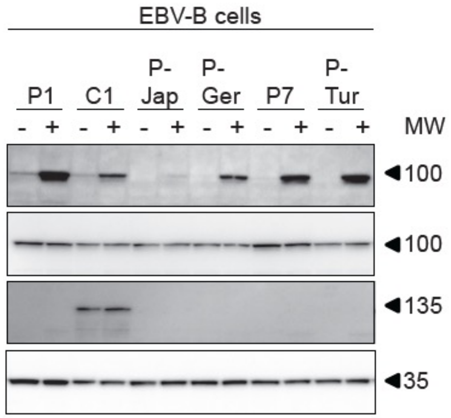

Anti-pSTAT3

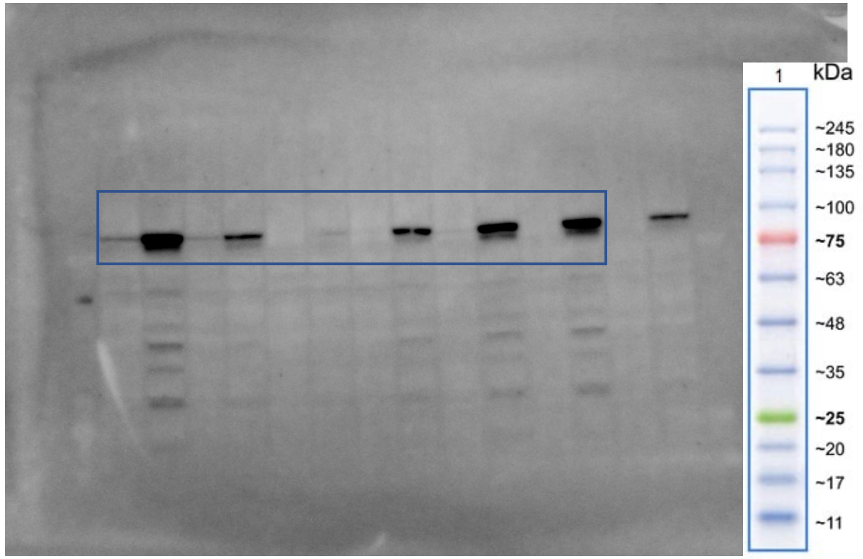

Anti-STAT3

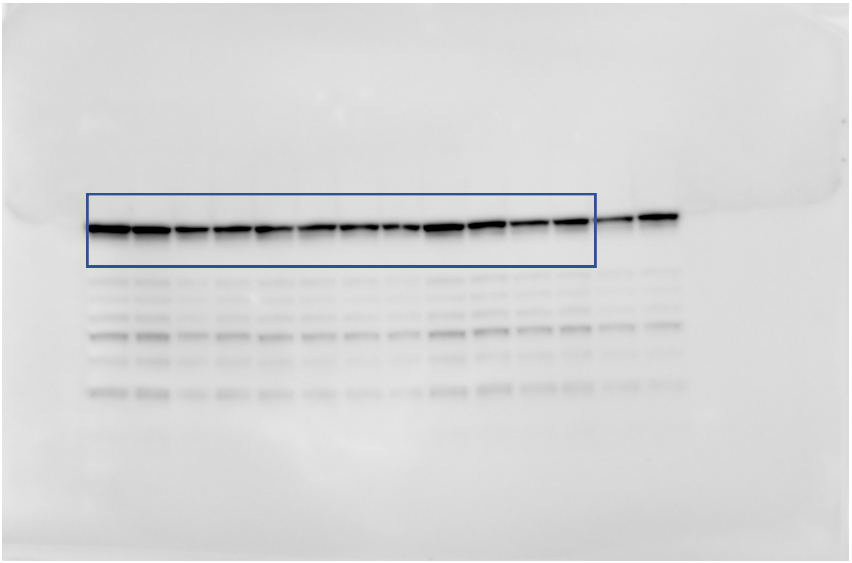

Anti-TYK2

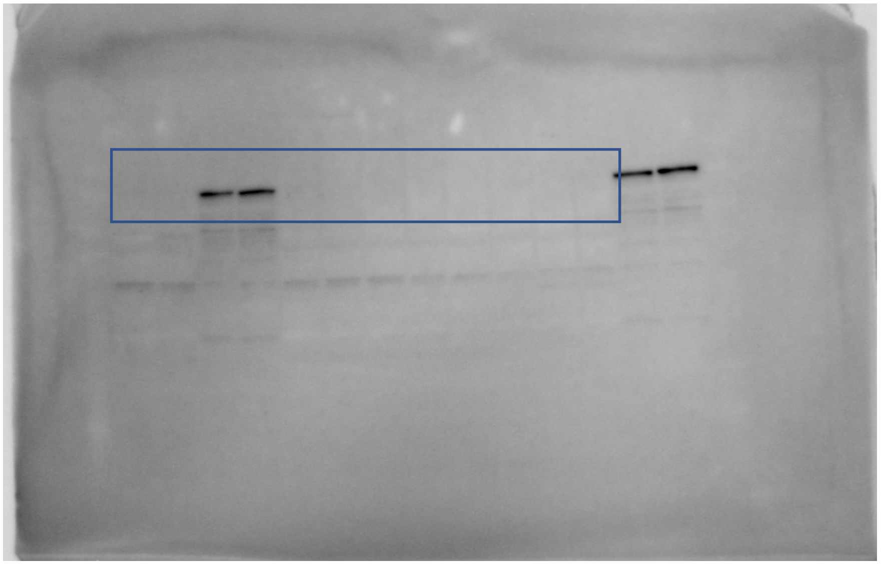

Anti-GAPDH

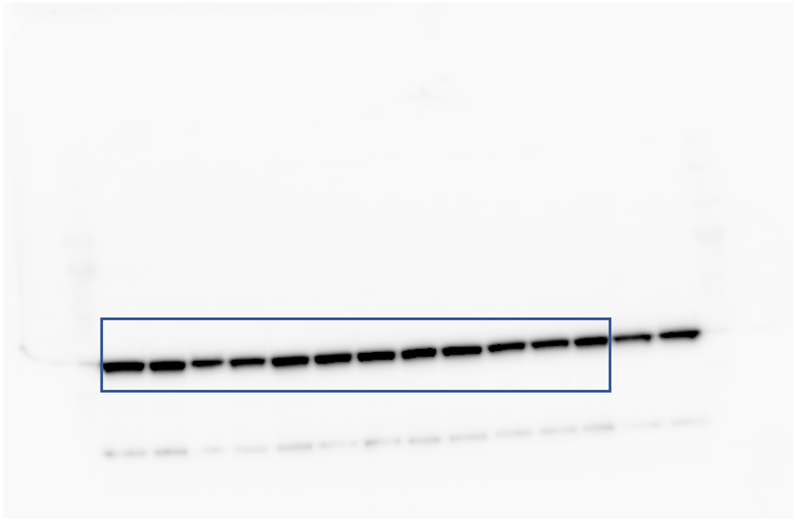

Figure 5A right

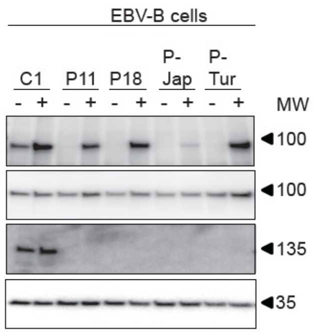

Anti-pSTAT3

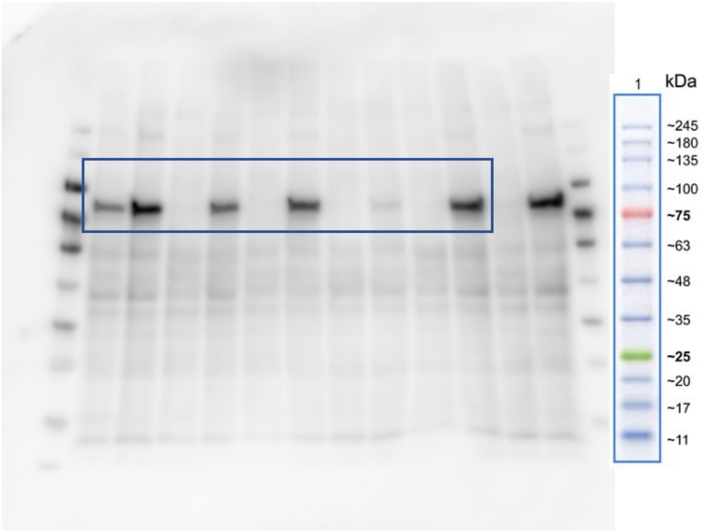

Anti-STAT3

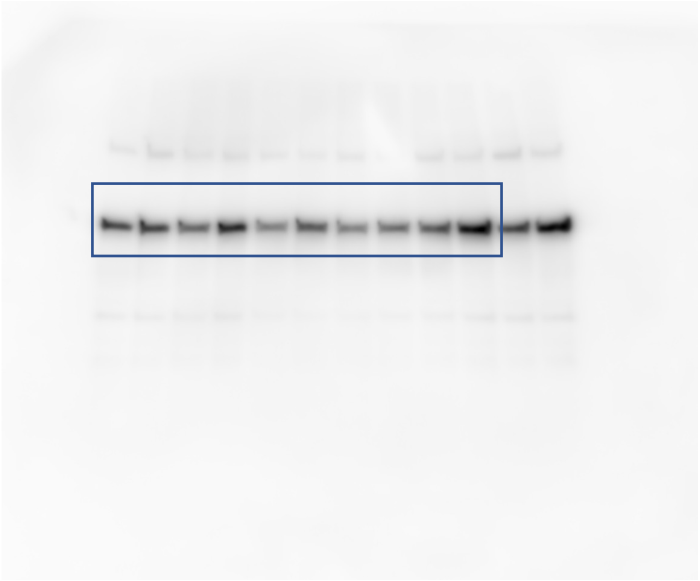

Anti-TYK2

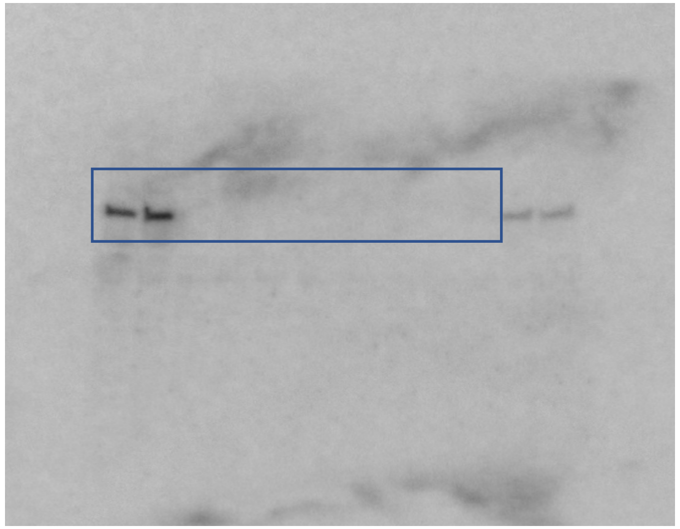

Anti-GAPDH

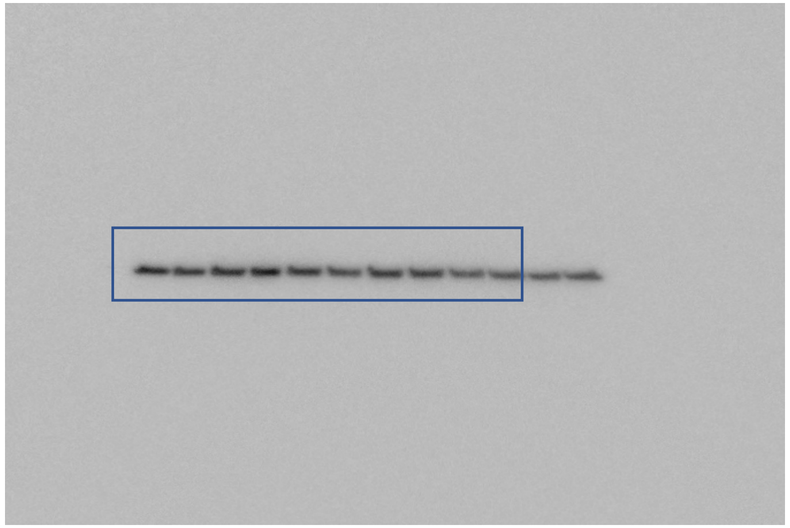

Supplement: SourceData F5 — contains original blots for Fig. 5. [file JEM_20220094_SourceDataF5.pdf]
