# Supplementary figures and images for: Impaired IL-23–dependent induction of IFN-γ underlies mycobacterial disease in patients with inherited TYK2 deficiency
Source: J Exp Med. 2022 Sep 12;219(10):e20220094. doi: 10.1084/jem.20220094 (PMC9472563; doi:10.1084/jem.20220094)

Supplementary Figure 2 B

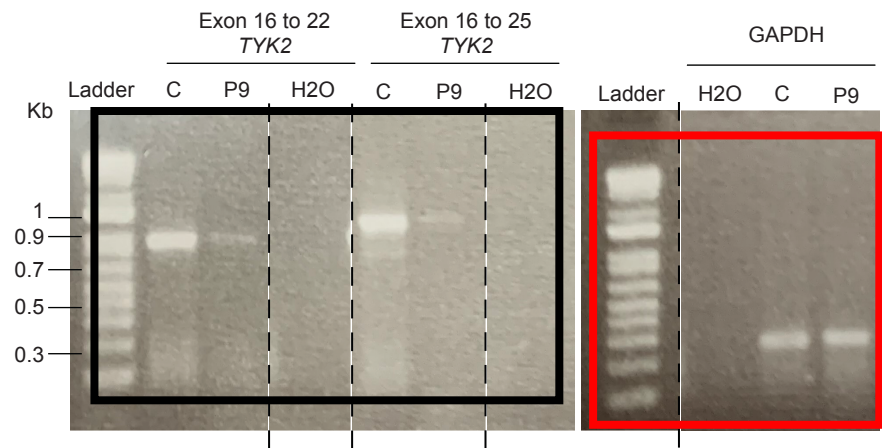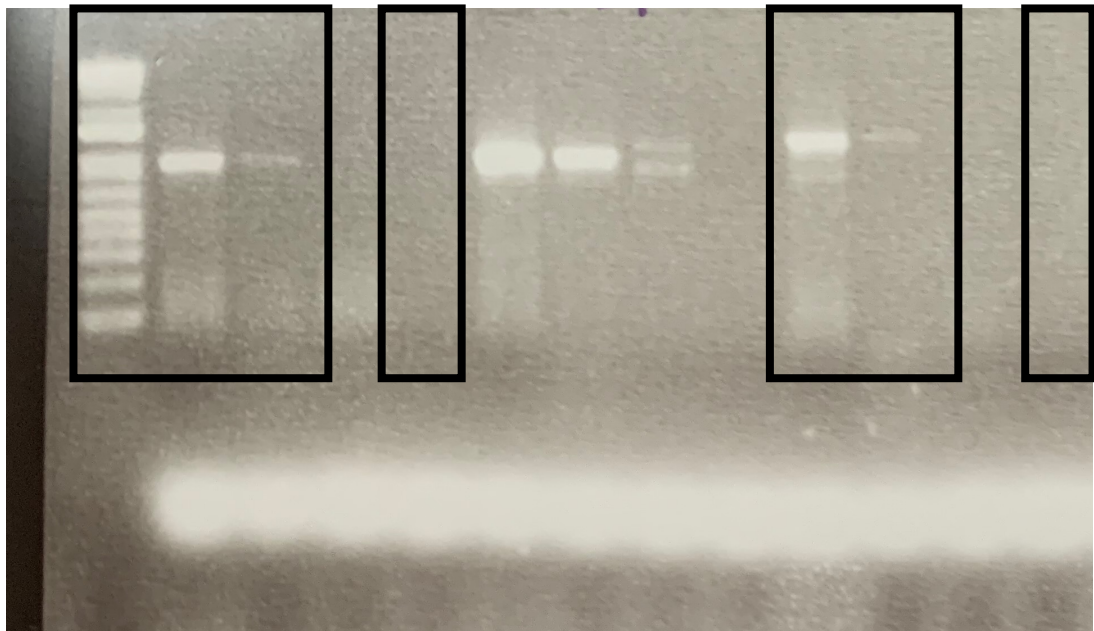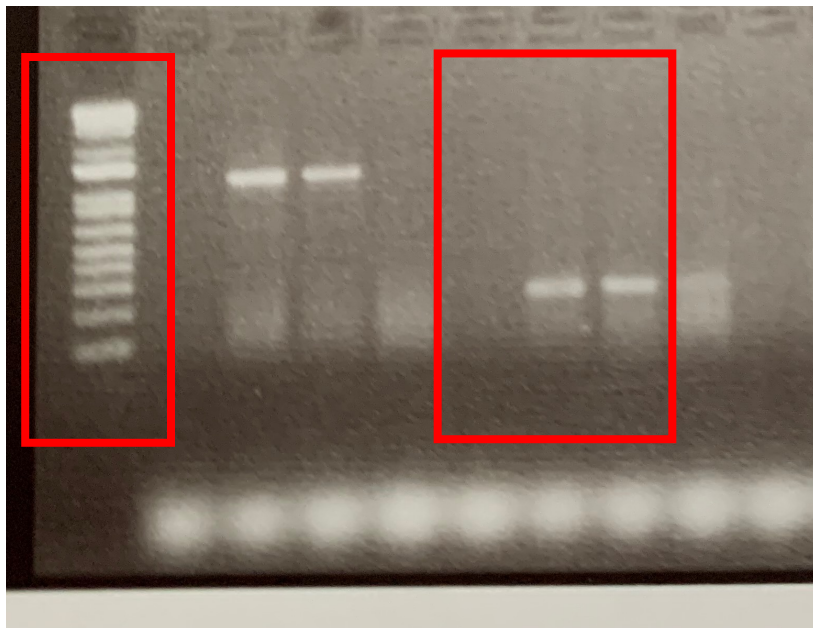

Supplement: SourceData FS2 — contains original blots for Fig. S2. [file JEM_20220094_SourceDataFS2.pdf]
